# SenFlag gene signature identifies senescent cells in mouse and human tissues through a conserved core transcriptional program

**DOI:** 10.1038/s44318-026-00845-6

**Published:** 2026-07-06

**Authors:** Abdullah Altulea, Sebastian Mackedenski, Jamil Nehme, Marco Demaria

**Affiliations:** European Research Institute for the Biology of Ageing (ERIBA), University Medical Center Groningen (UMCG), University of Groningen (RUG), Groningen, The Netherlands

**Keywords:** Cell Cycle, Chromatin, Transcription & Genomics, Molecular Biology of Disease

## Abstract

Identifying senescent cells via single-cell transcriptome profiling data remains challenging due to cellular heterogeneity and overlap with other cellular states. Here, we present SenFlag, a streamlined gene signature for enhanced identification of senescent cells based on integration of core gene expression features. SenFlag was derived through systematic assessment of bulk and single-cell RNA-sequencing datasets across multiple senescence models. It captures a conserved transcriptional program characterized by reduced expression of proliferation-associated genes and chromatin-associated genes (*HMGB1/2, HMGN2*), combined with upregulation of cell-cycle inhibitors (*CDKN1A/CDKN2A*) and of *CCND1*. Additionally, SenFlag incorporates lysosomal features, including increased expression of V-ATPase subunits and cathepsins. SenFlag identifies a rare but progressively accumulating population of senescent cells across tissues in both mice and humans in vivo, with enrichment in epithelial and endothelial compartments. SenFlag-positive cells increase with age and following tissue injury, and are reduced in datasets involving senescence-targeting interventions, supporting its specificity in vivo. Together, SenFlag provides a robust and interpretable signature for identifying senescent cells in single-cell datasets and facilitates the study of senescence across physiological and pathological contexts.

## Synopsis

Senescent cells are highly heterogeneous, making their reliable identification in single-cell RNA-sequencing datasets challenging. This study introduces SenFlag, a novel gene signature, that identifies senescent cells in vivo across ageing by leveraging conserved senescence-associated programs.

- SenFlag integrates expression changes of proliferation and chromatin architecture factors, cell-cycle inhibitors CDKN1A/2A, and lysosomal genes.
- SenFlag distinguishes senescence from quiescence and immune activation via combined CCND1 induction and HMG gene repression.
- SenFlag identifies senescent cells that accumulate with age across mouse and human tissues, particularly within epithelial and endothelial compartments.
- SenFlag detects reductions in senescent-cell abundance following senescence-targeting interventions.

## Abstract (web summary)

A novel gene signature integrates proliferation, chromatin architecture and lysosomal properties to detect accumulating senescent cells in vivo across ageing.

## Graphical abstract

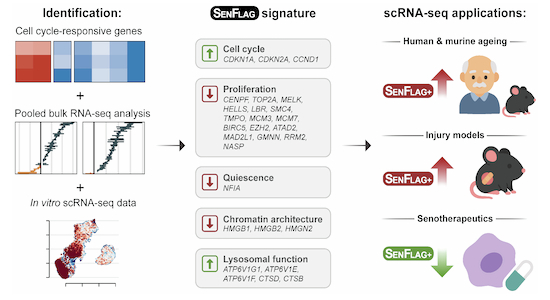

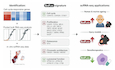

## Introduction

Cellular senescence is a state of stable cell cycle arrest triggered by various stressors, including DNA damage, telomere attrition, and oncogenic signaling (Kumari and Jat, 2021). Although senescent cells no longer proliferate, they remain metabolically active and frequently undergo functional changes that drive their detrimental effects (Coppé et al, 2010; Wiley and Campisi, 2021). Senescence-associated cell cycle arrest is mainly mediated by the activation of tumor suppressor pathways, such as p53/p21 and p16 (Kumari and Jat, 2021). A hallmark of senescent cells is the senescence-associated secretory phenotype (SASP), characterized by the secretion of pro-inflammatory cytokines, chemokines, growth factors, and proteases (Cuollo et al, 2020). In addition to SASP, lysosomal dysfunction is a robust hallmark of senescent cells, characterized by elevated senescence-associated β-galactosidase (SA-β-Gal) activity, impaired autophagic flux, accumulation of undegraded material such as lipofuscin, and compromised lysosomal membrane stability (Lee et al, 2006; Song et al, 2023; Tai et al, 2017; Tan and Finkel, 2023). Senescent cells accumulate progressively with age, contributing to tissue dysfunction and age-related pathologies (McHugh and Gil, 2018). Their presence has been implicated in various chronic diseases, including osteoarthritis, atherosclerosis, pulmonary fibrosis, and neurodegeneration, such as Alzheimer’s disease (Hernandez-Gonzalez et al, 2021; Liu, 2022; Liu et al, 2022; Wu et al, 2020).

Cellular senescence is a heterogeneous phenomenon, reflected in the wide range of markers and phenotypes that senescent cells exhibit (Cohn et al, 2023; Hernandez-Segura et al, 2017). This heterogeneity makes the detection of senescent cells particularly challenging in single-cell RNA-seq (scRNA-seq) data (Gurkar et al, 2023; Kim and Kim, 2021). In vitro senescence is typically validated via biochemical and imaging assays, such as SA-β-Gal staining and EdU incorporation, which cannot be directly translated to transcriptomic signatures (Debacq-Chainiaux et al, 2009; Hernandez-Segura et al, 2018; Valieva et al, 2022). In scRNA-seq, senescence is typically inferred from *CDKN1A* (p21) and *CDKN2A* (p16) expression in combination with SASP markers (Kiss et al, 2020; Wechter et al, 2023), though these markers are not exclusive to senescent cells and could potentially lead to misleading interpretations (Idda et al, 2020; Ogrodnik et al, 2024).

In this study, we aimed to define a core transcriptional phenotype of cellular senescence by focusing on its central hallmark, the stable cell cycle arrest. We analyzed multiple publicly available RNA-seq datasets from senescence-associated conditions, together with scRNA-seq data from cultured senescent cells, to identify genes consistently regulated across different conditions. Among the downregulated genes, *HMGB1, HMGB2, and HMGN2 (HMGB1/2/N2)* were prominent, alongside several cell cycle-related genes, including *MCM7, RRM2, TOP2A, SMC4*, and *CENPF*. Conversely, *CCND1* exhibited a paradoxical upregulation, coinciding with the nuclear localization of the CDK inhibitors p16 and p21. Furthermore, we observed a consistently elevated expression of the V-ATPase subunits *ATP6V1G1, ATP6V1E1*, and *ATP6V1F*, along with the cathepsins *CTSD* and *CTSB*. We identified cells in vivo that closely matched the transcriptional signature of in vitro senescent cells, revealing conserved transcriptional features of cellular senescence.

## Results

### Identification of cell cycle-responsive genes

**Figure 1:**
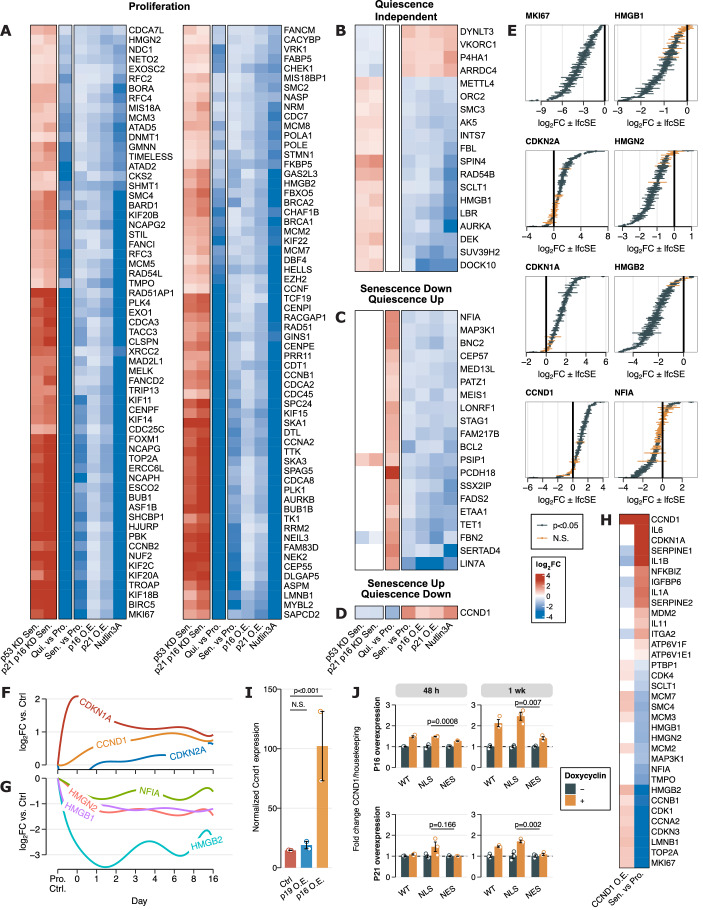
Identification of cell cycle–associated and senescence-related transcriptional features. (**A**–**D**) Heatmaps showing log₂ fold change values for genes across conditions associated with changes in cell-cycle activity. Genes were grouped based on their association with (**A**) proliferation, (**B**) quiescence-independent regulation, (**C**) downregulation in senescence and upregulation in quiescence, and (**D**) upregulation in senescence and downregulation in quiescence. Differential expression was computed using DESeq2 (*p*_adj_ < 0.05). (**E**) Summary of log₂ fold change values (±lfcSE) for selected genes across multiple senescence-associated bulk RNA-seq datasets (*n* = 140), including both mouse and human samples. (**F**,** G**) Temporal log₂ fold change of indicated genes in WI-38 cells following doxorubicin-induced senescence, showing early and sustained transcriptional changes relative to control conditions. (**H**) Heatmap comparing transcriptional changes following *CCND1* overexpression versus senescence induction (irradiation), highlighting differential regulation of proliferation- and senescence-associated genes. (**I**) Log₂ fold change of *Ccnd1* expression following overexpression of *Cdkn2a* isoforms, illustrating distinct responses to *p16INK4A* and *p19ARF*. Adjusted *p* value for p16 O.E. vs. ctrl. is 1e-05. (*n* = 2, biological replicates of T cells) (**J**) RT–qPCR analysis of *CCND1* expression following induction of *p16* and *p21* constructs with nuclear localization (NLS), nuclear export (NES), or wild-type (WT) configurations. Data were shown as mean ± SEM (*n* = 3, biological replicates of BJ fibroblasts). Statistical comparisons were performed using a two-tailed *t*-test. Source data are available online for this figure.

Senescent cells downregulate genes that are associated with proliferation. To get a better overview of what these genes are, we performed an integrated analysis of conditions with changes in cell cycle activity. Additionally, since quiescent cells also downregulate proliferation markers, we aimed to identify markers that distinguish quiescent cells from senescent cells. To represent cell cycle suppression, we included an irradiation-induced senescent cells experiment previously performed in our laboratory (Hernandez-Segura et al, 2017), cells with p16 or p21 overexpression or treated with Nutlin3a to induce p53 (Li et al, 2023; Sturmlechner et al, 2021), and quiescent cells (Lenain et al, 2017). To represent cell cycle activation in the context of cellular senescence, we included senescent cells with p53, p16, or p21 knockdown (Tasdemir et al, 2016). We filtered the genes based on significance and grouped them into four categories: proliferation-associated (Fig. 1A), quiescence-independent (Fig. 1B), senescence down/quiescence up (Fig. 1C), and senescence up/quiescence down (Fig. 1D).

**Figure EV1:**
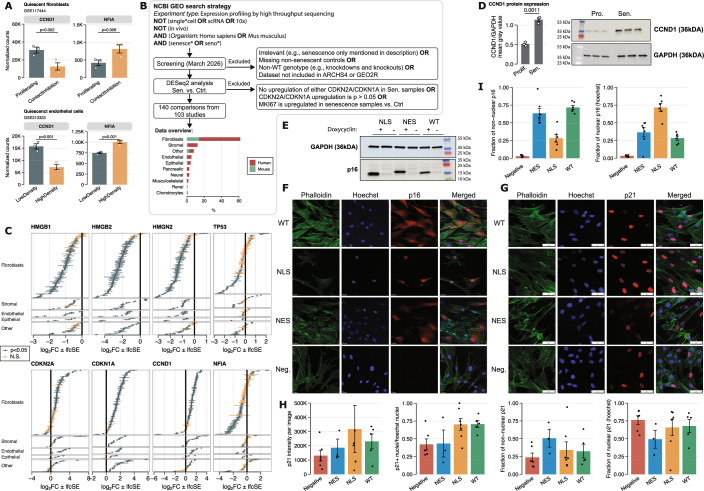
Supporting analyses for identification of cell cycle–associated and quiescence-related transcriptional features. (**A**) Normalized expression levels of *CCND1* and *NFIA* in quiescent endothelial cells and fibroblasts, induced by contact inhibition (*n* = 3 biological replicates per condition). For GSE213323, the adjusted *p* values are 2e-09 for *CCND1* and 0.00269 for *NFIA*. Statistical values were obtained using DESeq2. (**B**) Overview of the search strategy used to identify senescence-associated datasets from NCBI GEO for pooled analysis. (**C**) Log₂ fold change values (±lfcSE) from DESeq2 for indicated genes across datasets identified in (**B**), stratified by cell type. *TP53* results include the murine ortholog *Trp53*. *N* = 139–140 senescent vs. ctrl. comparisons from 103 unique studies. (**D**) Western blot analysis of *CCND1* expression in senescent IMR90 cells treated once with 250 µM doxorubicin and collected after 14 days. Quantification is shown relative to GAPDH (fluorescent) (*n* = 3 biological replicates per condition). Statistical comparisons were performed using a two-tailed *t*-test. Bars represent group means. Error bars indicate ±standard error of the mean. (**E**) Western blot analysis of p16 expression in BJ cells across indicated constructs and doxycycline conditions following 48 h of treatment (doxycycline or DMSO control). (**F**,** G**) Representative immunofluorescence images of BJ cells showing subcellular distribution of p16 (**F**) and p21 (**G**) under the indicated localization constructs. (**H**,** I**) Quantification of immunofluorescence images from panels (**F**) and (**G**), including signal intensity and colocalization with nuclear staining, for p21 (**H**) and p16 (**I**) (*n* = 5 images from independent samples). Bars represent group means. Error bars indicate ±standard error of the mean. Source data are available online for this figure.

**Figure EV2:**
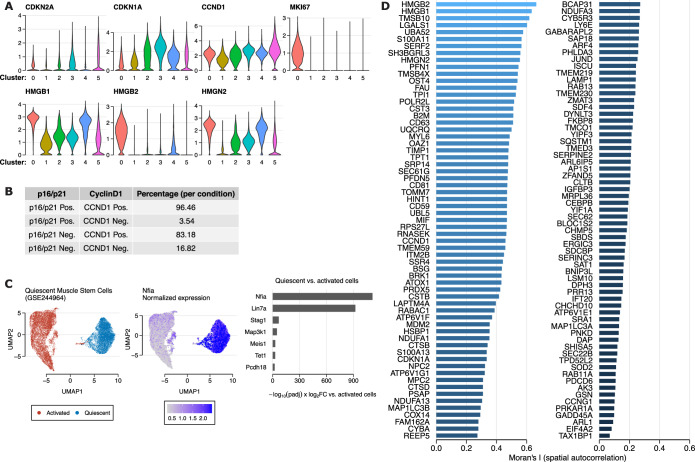
Single-cell characterization of senescence- and quiescence-associated transcriptional features. (**A**) Violin plots showing normalized expression of indicated genes across Louvain clusters from the integrated in vitro senescent cell dataset (Fig. 2C) (*n* = 3k–18k cells/cluster; 68k cells in total). (**B**) Numerical representation corresponding to the river plot shown in Fig. 2K. (**C**) Analysis of quiescent muscle stem cell data showing cell annotations, normalized *Nfia* expression, and candidate marker genes distinguishing quiescent from activated states. Gene selection was based on the results from Fig. 1C. (**D**) Top genes identified from pseudotime analysis based on spatial expression patterns, presented as Moran’s I spatial correlation values.

The analysis identified several genes associated with proliferation. Additionally, we found that *HMGB1* and *HMGB2*, markers previously linked to cellular senescence (Sofiadis et al, 2021; Zirkel et al, 2018), were in this set, along with *HMGN2*. Transcriptionally, *HMGB2* and *HMGN2* correlated with changes in proliferation, whereas *HMGB1* did not. Among the quiescence markers (Fig. 1C), *NFIA* consistently increased during quiescence in scRNA-seq data (further analyzed in Fig. EV2C) and thus served as a quiescence-exclusion marker for our method. Notably, *NFIA* is upregulated following both serum deprivation (Fig. 1C) and contact inhibition (Fig. EV1A), suggesting that its expression is not simply a direct consequence of serum deprivation, but instead reflects a quiescent cellular state. *CCND1* uniquely behaved as a marker up in senescence and down in quiescence and was reduced by p53/p16/p21 knockdown, suggesting an association with cell-cycle arrest in the presence of active cell-cycle signaling. The upregulation of *CCND1* in senescent cells likely reflects a transcriptional attempt to bypass cell cycle arrest.

To validate these results, we performed a systematic analysis of senescence-associated bulk RNA-seq datasets from NCBI GEO (mouse and human; Fig. EV1B). The pooled bulk RNA-seq analysis showed a consistent increase in *CCND1*, while *HMGB1/2/N2* and *NFIA* were downregulated (Fig. 1E). Importantly, stratification by cell type revealed no cell-type–specific differential expression for the indicated genes (Fig. EV1C). Notably, *TP53* transcript levels showed an inconsistent decrease in senescent cells despite established pathway activation in senescence (Fig. EV1C), indicating that *TP53* may not reliably reflect pathway activity.

A time-course dataset of doxorubicin-treated WI-38 cells (Suda et al, 2024) showed that *CCND1* levels increased shortly after senescence induction and remained elevated over 16 days in parallel with *CDKN1A/CDKN2A* (Fig. 1F), whereas *HMGB1/2/N2* and *NFIA* levels decreased within 24 h and remained low (Fig. 1G). *CCND1* protein levels increased correspondingly (Fig. EV1D).

Analyzing *CCND1* overexpression in epithelial cells (Sack et al, 2018) showed that *CCND1* increased the expression of proliferation-associated markers, including *TOP2A, MKI67*, and *MCM7*, but did not induce senescence/SASP genes (Fig. 1H). This indicates that *CCND1*, outside the context of senescence, does not on its own induce a senescence-associated transcriptional program.

We further identified *CCND1* as a readout for *CDKN2A* isoform activity, being increased with overexpression of p16INK4A but not p19ARF (Fig. 1I).

Finally, using Tet-On inducible p16/p21 constructs with nuclear localization or export signals (Fig. EV1E–I), we demonstrated that *CCND1* increased only when inhibitors were localized to the nucleus (Fig. 1J), supporting the interpretation that *CCND1* upregulation reflects a state of cell-cycle arrest occurring in the presence of pro-proliferative signaling. These data support the use of *CCND1* as a context-dependent transcriptional marker of cell-cycle arrest when combined with *CDKN1A* or *CDKN2A* expression. Thus, proliferation markers, *HMGB1/2/N2*, *NFIA*, and *CCND1* (in combination with *CDKN2A* or *CDKN1A*) form the basis of the SenFlag signature.

### In vitro scRNA-seq definition of bulk-derived core senescence gene signature

**Figure 2:**
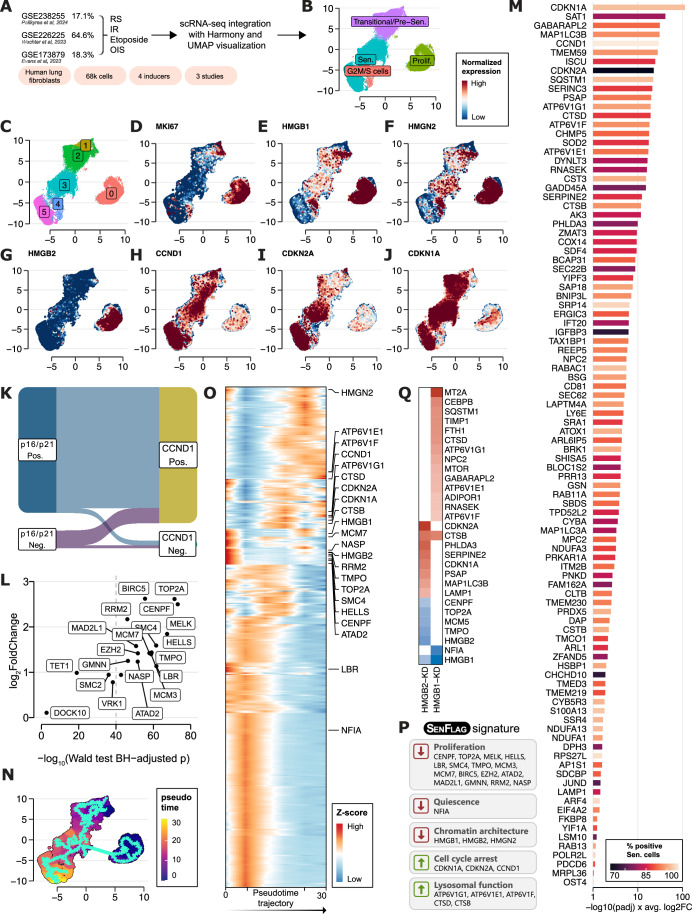
Definition of a core senescence-associated transcriptional program from integrated single-cell data. (**A**) Overview of the integration strategy used to analyze single-cell transcriptomic profiles of in vitro senescent cells across multiple senescence models. (**B**) UMAP visualization of the integrated dataset showing cell populations with annotated clusters. (**C**) UMAP displaying clustering results obtained using the Louvain algorithm. (**D**–**J**) Normalized expression of selected genes projected onto the UMAP embedding, illustrating characteristic transcriptional features associated with proliferating and senescent cell populations. (**K**) River plot showing the distribution of cells based on *CDKN2A*, *CDKN1A*, and *CCND1* expression. (**L**) Candidate proliferation markers, derived from Fig. 1A, were refined based on consistent upregulation in the proliferating cluster. (**M**) Bar plot showing padj-weighted log₂ fold change values for genes consistently upregulated in senescent cells, identified through combined analysis of bulk and scRNA-seq datasets. *P* values were computed using the metafor::rma function. (**N**) Pseudotime trajectory projected onto the UMAP embedding. (**O**) Heatmap summarizing gene expression dynamics along the pseudotime trajectory. (**P**) Schematic representation of the SenFlag criteria, integrating reduced proliferation, reduced HMG gene expression, reduced NFIA expression, and combined expression of *CDKN1A or CDKN2A* with *CCND1* and lysosomal markers. (**Q**) Heatmap showing log₂ fold change values for indicated genes following *HMGB1* or *HMGB2* knockdown (KD) relative to controls (*p*_adj_ < 0.05). Blank tiles indicate non-significant changes (*p*_adj_ ≥  0.05). Statistical significance values were computed using DESeq2.

After defining markers of proliferation and cell cycle arrest, we tested whether these genes were conserved at the single-cell resolution across various senescence models in vitro. We integrated scRNA-seq datasets spanning replicative senescence, DNA damage (irradiation and etoposide), and oncogene-induced senescence (Evans et al, 2023; Palikyras et al, 2024a,b; Wechter et al, 2023) (Fig. 2A). Dimensionality reduction and clustering separated proliferating from senescent populations with a minor G2/M/S fraction and six Louvain sub-clusters (Fig. 2B,C). Senescent clusters consistently showed loss of *HMGB1/2/N2*, and *MKI67*, together with upregulation of *CCND1, CDKN1A, and CDKN2A* (Figs. 2D–J and EV2A). More than 95% of *CDKN1A/CDKN2A*+ cells co-expressed *CCND1* (Figs. 2K and EV2B). To accurately capture proliferation loss in our method, we first identified the top genes within the proliferating cluster and then cross-referenced them with markers from the results shown in Fig. 1A. This approach highlighted *NASP, TMPO, LBR, MCM7, SMC4, CENPF, RRM2, TOP2A, ATAD2*, and *HELLS* as candidate genes for this purpose (Fig. 2L). A similar analysis of ex vivo quiescent muscle stem cells (Girolamo et al, 2024) confirmed that *Nfia* was upregulated during quiescence and downregulated upon activation (Fig. EV2C). We combined data from the pooled bulk analysis with single-cell data and retained significant genes (*p*_adj_ < 0.001) that were upregulated in ≥70% of senescent cells to identify a set of consistently upregulated genes in senescent cells (Fig. 2M). These included senescence and cell-cycle regulators (*CDKN2A, CDKN1A,* and *CCND1*), autophagy machinery (*MAP1LC3B, GABARAPL2,* and *SQSTM1*), lysosomal and vesicular genes (*ATP6V1F, ATP6V1E1, ATP6V1G1, CTSD, CTSB,* and *PSAP*), mitochondrial and oxidative stress regulators (*NDUFA1, NDUFA13, MPC2, PRDX5,* and *CYBA*), vesicle trafficking and endomembrane genes (*RAB13, ARF4, TMED3, AP1S1,*and *CLTB*), and membrane, adhesion, and extracellular remodeling factors (*CD81, BSG,* and *SERPINE2*). Pseudotime analysis using monocle3 (Fig. 2N) tracked the expression of *CDKN2A*, reflecting the senescence-maturation trajectory, along which *CCND1*, cathepsins, and V-ATPase subunits also increased (Figs. 2O and EV2D).

Based on the results obtained so far, we defined a minimal set (henceforth referred to as SenFlag) requiring reduced proliferation markers, reduced *NFIA*, reduced *HMGB1/2/N2*, along with positivity (i.e., at least one transcript) for either *CDKN1A* or *CDKN2A* together with *CCND1*, and any V-ATPase subunit together with any cathepsin (Fig. 2P). Interestingly, analysis of *HMGB1/2* knockdown datasets (Sofiadis et al, 2021; Zirkel et al, 2018) revealed a signature similar to that of SenFlag. Notably, *HMGB2* knockdown itself increased *CDKN1A/CDKN2A* expression and reduced *HMGB1* expression (Fig. 2Q), linking *HMGB1/2* to the transcriptional profile of senescent cells.

These results suggest that the senescence transcriptome contains a conserved core program defined by cell-cycle arrest, complemented by additional modules reflecting changes in markers of chromatin, lysosomes, mitochondria, and protein homeostasis. Importantly, this core program represents a minimal and conserved transcriptional framework that can be applied across heterogeneous datasets, and may be complemented by additional context-dependent features such as SASP-related or lineage-specific programs.

### In vivo identification of senescent cells across aging and DNA damage contexts

**Figure 3:**
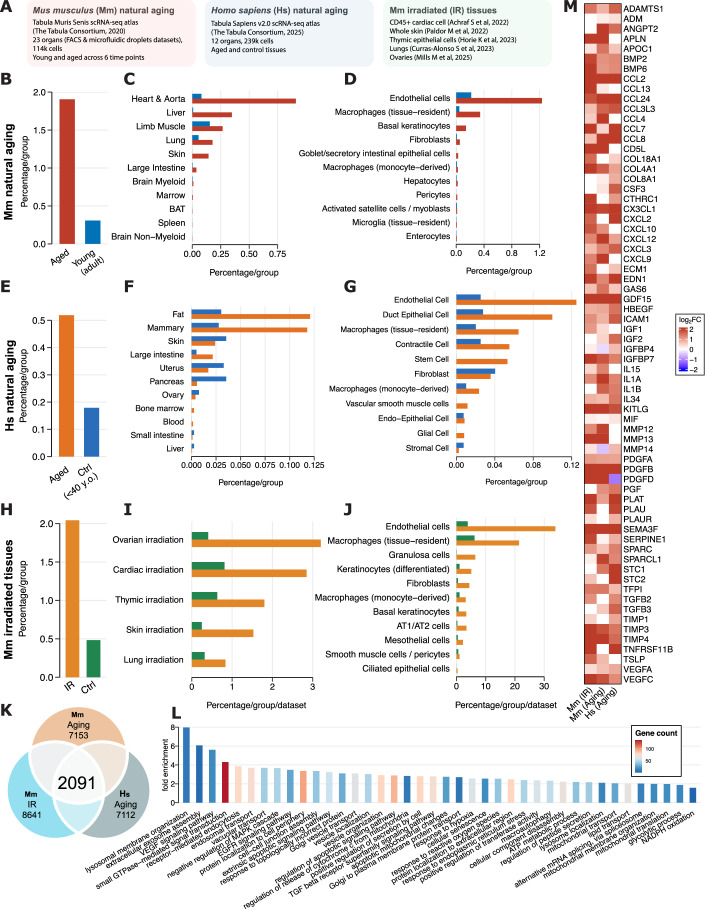
In vivo identification of SenFlag+ cells across aging and DNA damage contexts. (**A**) Overview of the datasets analyzed, including murine and human aging atlases and irradiation models. (**B**) Bar plot showing the abundance of SenFlag+ cells relative to all cells in aged and young samples from the Tabula Muris dataset (murine natural aging). (**C**) Distribution of SenFlag+ cells across murine tissues, stratified by age (Tabula Muris). (**D**) Distribution of SenFlag+ cells across cell types, stratified by age (Tabula Muris). (**E**) Bar plot showing the abundance of SenFlag+ cells relative to all cells in control and aged samples from the Tabula Sapiens dataset (human natural aging). (**F**) Distribution of SenFlag+ cells across human tissues, stratified by age (Tabula Sapiens). (**G**) Distribution of SenFlag+ cells across human cell types, stratified by age (Tabula Sapiens). (**H**) Bar plot showing the average abundance of SenFlag+ cells in irradiated and control samples across datasets. Values are presented as averages across studies rather than cumulative abundance since datasets originate from different sources. (**I**) Distribution of SenFlag+ cells across murine tissues, normalized per dataset and stratified by irradiation status. (**J**) Distribution of SenFlag+ cells across cell types, stratified by irradiation status. Cell types reflect those captured in each dataset. (**K**) Venn diagram showing the overlap of significantly regulated genes in SenFlag+ cells from human and murine tissues compared to condition-matched SenFlag- cells. (**L**) Gene ontology (GO) enrichment analysis showing fold enrichment of significantly enriched terms based on overlapping genes in panel (**K**). (**M**) Heatmap showing log₂ fold change of genes encoding secreted proteins that are consistently upregulated across conditions, illustrating transcriptional features associated with secretory and inflammatory programs in SenFlag+ cells.

We applied the SenFlag signature to multi-tissue and multi-species datasets to determine the abundance, cell-type distribution, and molecular characteristics of senescent cells in vivo. Results from Tabula Muris Senis (Consortium, 2018), Tabula Sapiens 2.0 (Consortium and Quake, 2025), and irradiated murine tissues (Curras-Alonso et al, 2023; Horie et al, 2023; Mills et al, 2025; Paldor et al, 2022; Shamseddine et al, 2023) (Fig. 3A) revealed a rare but measurable abundance of senescent cells (i.e., SenFlag+ cells) that increased with age and after DNA damage. Across species, the fraction of senescent cells increased with age. In mice, senescent cells rose roughly sixfold, reaching just under 2% of total cells, with the cardiovascular system, liver, and kidneys showing the highest abundance among the tested tissues (Fig. 3B, C). Endothelial cells, epithelial cells, fibroblasts, and a subset of macrophages comprised the majority of this population (Fig. 3D). In humans, senescent cells represented ~0.5% of total cells and increased threefold between 26–38 and 45–61 years, with fat and mammary tissues exhibiting the highest abundance among the tested tissues (Fig. 3E,F). As in mice, endothelial, epithelial, and tissue-resident macrophage subsets dominated the senescent pool (Fig. 3G). In irradiated tissues, the fraction of senescent cells increased roughly fourfold, averaging around 2% of total cells, with ovarian, cardiac, and thymic tissues showing the highest abundance among the tested tissues (Fig. 3H,I). Endothelial cells, macrophages, epithelial cells, and fibroblasts constituted the majority of the senescent population, consistent with the patterns observed in aging tissues (Fig. 3J).

**Figure EV3:**
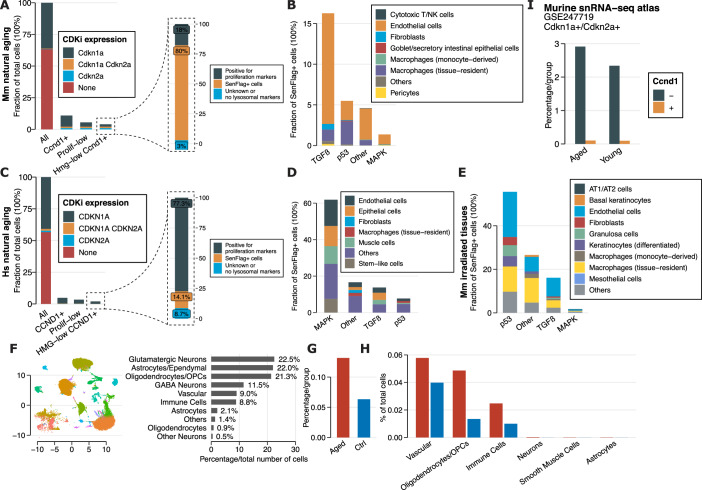
Stepwise application of SenFlag and pathway characterization across in vivo datasets. (**A**) Bar plot showing the cellular distribution of cells at successive steps of SenFlag application in the Tabula Muris dataset (murine aging). (**B**) Fraction of SenFlag+ cells expressing downstream targets of indicated pathways in the Tabula Muris dataset, stratified by cell type. (**C**) Bar plot showing the cellular distribution of cells at successive steps of SenFlag application in the Tabula Sapiens dataset (human aging). (**D**) Fraction of SenFlag+ cells expressing downstream targets of indicated pathways in the Tabula Sapiens dataset, stratified by cell type. (**E**) Fraction of SenFlag+ cells expressing downstream targets of indicated pathways in irradiated murine tissue datasets (see Fig. 3I), stratified by cell type. (**F**) UMAP visualization of integrated murine scRNA-seq datasets from brain and blood–brain barrier tissues, showing cellular composition. (**G**) Abundance of SenFlag+ cells in brain datasets from panel (**F**), stratified by age. (**H**) Abundance of SenFlag+ cells in brain datasets from panel (**F**), stratified by cell type and age. (**I**) Abundance of *Cdkn1a/Cdkn2a* + , *Hmgb1/2/n2*-low cells stratified by *Ccnd1* positivity in single-nucleus (snRNA-seq) data.

Analysis of the cellular pool revealed that *CDKN1A* was broadly expressed in mouse tissue (>30% of cells; Fig. EV3A), whereas *CDKN2A* transcripts were detected at low frequency, consistent with known technical limitations of scRNA-seq in capturing low-abundance transcripts. Importantly, SenFlag+ cells represented only a small subset of *CDKN1A/CDKN2A*+ cells. To identify potential inducers of in vivo senescence, we applied PROGENy (Schubert et al, 2018) to infer pathway activation. The analysis showed TGFβ and p53 bias in murine aging (Fig. EV3B). *CDKN1A* and *CDKN2A* abundance in humans mirrored that of mice (Fig. EV3C). However, PROGENy analysis showed MAPK bias in human aging (Fig. EV3D), and p53 in irradiation (Fig. EV3E).

To derive brain-specific abundance data, we integrated multiple scRNA-seq datasets from aged and young murine brains, collecting over one million cells (Jin et al, 2025; Wu et al, 2025; Ximerakis et al, 2023; Zhao et al, 2020). Senescent cells were rare across these datasets and primarily restricted to vascular cells and oligodendrocyte progenitors (Fig. EV3F–H).

Cross-species differential analysis identified 2091 genes that were consistently altered in senescent cells in vivo (Fig. 3K). These genes are associated with GO terms related to vesicle and lysosomal trafficking, growth factor signaling, apoptosis regulation, oxidative stress responses, mitochondrial and metabolic remodeling, and cellular senescence (Fig. 3L).

While the SenFlag signature does not include SASP factors in its selection criteria, SenFlag+ cells upregulate genes that encode secreted proteins involved in inflammation, growth factor signaling, extracellular matrix remodeling, vascular activation, and stress responses, indicating that SASP-associated transcriptional programs emerge as downstream features of SenFlag+ cells, despite not being part of the signature’s criteria (Fig. 3M).

Notably, *CCND1* transcripts were undetectable in single-nucleus RNA-seq data (Fig. EV3I), highlighting a limitation of applying SenFlag to snRNA-seq datasets.

These results indicate that senescent cells comprise a minor but increasing proportion of total cells with age and are particularly prevalent in endothelial cells, epithelial cells, and tissue-resident macrophages. While they exhibit species-specific inducer biases, they share a conserved core transcriptional profile.

### Distinctive features of injury-associated senescence

**Figure 4:**
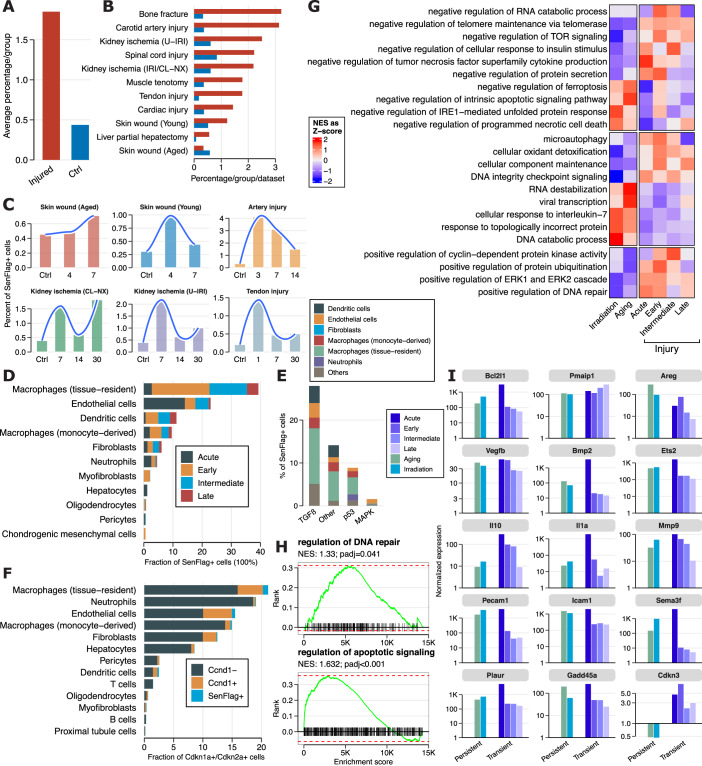
Transcriptional features of injury-associated SenFlag+ cells. (**A**) Bar plot showing the average abundance of SenFlag+ cells across datasets indicated in (**B**) stratified by injury condition. Values are reported as averages across studies rather than cumulative abundance since datasets originate from different sources. (**B**) Bar plot showing the abundance of SenFlag+ cells in each dataset, stratified by injury condition. (**C**) Bar plots showing temporal changes in the abundance of SenFlag+ cells across different injury models. (**D**) Distribution of cell types among SenFlag+ cells across different injury phases. (**E**) Fraction of SenFlag+ cells expressing downstream targets of the indicated pathways, stratified by cell type. (**F**) Distribution of *Cdkn1a/Cdkn2a*+ cells stratified by *Ccnd1* positivity and SenFlag status. (**G**) Heatmap showing z-scores of normalized enrichment scores (NES) from GSEA for the indicated GO terms across injury phases and aging/irradiation conditions. (**H**) Barcode plots showing enrichment of the GO terms GO:0006282 (top) and GO:2001233 (bottom), comparing injury-associated SenFlag+ cells with those identified in aging and irradiation contexts. Adjusted *p*-value for GO:2001233 is 1e-05. Statistical significance was assessed using fgsea. (**I**) Normalized expression values of indicated genes in SenFlag+ cells across injury phases, as well as aging and irradiation conditions. Data were presented in log₁₀ space.

**Figure EV4:**
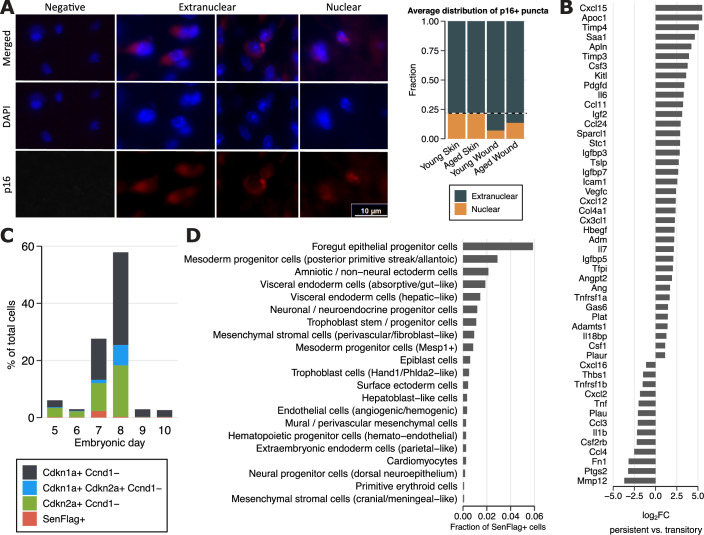
Additional analyses of SenFlag+ cells across injury, aging, and developmental contexts. (**A**) Immunofluorescence staining showing representative images of p16 subcellular distribution (negative, extranuclear, and nuclear) and corresponding quantification in young and aged, wounded and unwounded murine skin. The dashed line indicates the average fraction of p16 distribution in intact skin. (**B**) Log₂ fold change values (*p*_adj_ <  0.05; DESeq2) for indicated inflammation-associated genes comparing SenFlag+ cells from injury-associated datasets with those from aging and irradiation datasets. (**C**) Cellular distribution of *Cdkn1a/Cdkn2a*+ cells across the indicated murine developmental stages (in days), stratified by *Ccnd1* positivity and SenFlag status. (**D**) Cell type distribution of SenFlag+ cells in murine developmental datasets. Source data are available online for this figure.

To determine whether SenFlag distinguishes injury-associated senescence from persistent senescence, we analyzed scRNA-seq datasets from a broad spectrum of injury models. Analysis encompassing skin, muscle, tendon, bone, spinal cord, carotid artery, heart, liver, and kidney (Cortada et al, 2024; He et al, 2022; Huang et al, 2025; King et al, 2025; Modares et al, 2025; Tian et al, 2025; Vu et al, 2022; Warwick et al, 2023; Xu et al, 2022; Zhang et al, 2024a–g) revealed an approximate fourfold increase in SenFlag+ cells following injury (Fig. 4A), with the highest levels observed in bone fracture, carotid artery injury, and kidney ischemia–reperfusion models among the tested datasets (Fig. 4B). Additionally, the SenFlag signature effectively tracked the temporal induction of senescent cells across multiple injury models (Fig. 4C). A substantial fraction of SenFlag+ cells corresponded to tissue-resident macrophage subsets, followed by endothelial cells, other immune cells, and fibroblasts (Fig. 4D), with ~25% of them being TGFβ+ and ~10% p53+ (Fig. 4E). Notably, only a small subset of injury-induced *Cdkn1a/Cdkn2a+* cells co-expressed *Ccnd1* (Fig. 4F), suggesting that *CDKN1A/CDKN2A* expression in these cells may not always correspond to a fully established cell-cycle arrest state. This interpretation is supported by p16 immunostaining of aged and young wounded skin, which revealed mostly cytoplasmic, not nuclear, p16 (Fig. EV4A).

Comparative GO enrichment analysis of SenFlag+ cells in aging, irradiation, and injury data highlighted terms related to stress adaptation, proteostasis, DNA damage responses, and survival signaling (Fig. 4G). Notably, DNA repair and apoptotic signaling were upregulated in transient SenFlag+ cells versus persistent SenFlag+ cells of aging and irradiation (Fig. 4H). At the gene level, sustained *Pmaip1* upregulation is consistent with increased expression of apoptosis-related genes in injury-associated senescence. While acute-phase *Bcl2l1* induction likely provides a temporary survival buffer, the subsequent decline of *Bcl2l1* alongside persistent or increased *Pmaip1* at later stages is suggestive of increased susceptibility to clearance during resolution. The transcriptional profile further includes DNA damage and stress-response genes (*Gadd45a*), pro-inflammatory (*Il1a*) and anti-inflammatory (*Il10*) signaling, transient repair-associated mediators (*Areg*), growth factors (*Bmp2, Ets2,* and *Vegfb*), and extracellular matrix remodeling, adhesion, and migration genes (*Mmp9, Plaur, Sema3f*, *Icam1,* and *Pecam1*). This transcriptional profile is consistent with a regulated, temporally dynamic response associated with tissue injury and resolution, in contrast to the more persistent transcriptional features observed in aging and irradiation contexts (Fig. 4I). Overall, the data suggest that persistent senescent cells are more inflammatory than transient ones (Fig. EV4B). Interestingly, we found the CDK inhibitor *Cdkn3* to be selectively upregulated in injury senescent cells (Fig. 4I).

Analysis of embryonic datasets (E5.5–E10.5) (Azami et al, 2025; Beckröge et al, 2025; Chan et al, 2019; Chen et al, 2024; Cheng et al, 2022; Krup et al, 2023; Zeng et al, 2023) revealed that *Cdkn1a*/*Cdkn2a*+ cells were widespread, but most lacked *Ccnd1* co-expression (Fig. EV4C). In contrast, SenFlag positivity was sparse and inconsistently distributed, with enrichment in epithelial and mesodermal progenitor cells (Fig. EV4D).

Overall, injury-associated senescence represents a temporally regulated and functionally distinct state marked by repair and controlled clearance transcriptional profiles, whereas persistent senescence in aging and DNA damage is associated with a more sustained pro-inflammatory and survival-associated transcriptional profile, thus defining two biologically and transcriptionally distinct modes within a shared framework of senescence. These observations reflect population-level dynamics and do not directly establish the fate or duration of individual senescent cells.

### Activated macrophages and senescent cells exhibit distinct transcriptional phenotypes

Macrophages can express *CDKN1A* and inflammatory genes upon activation, thus overlapping with senescent cells. Conversely, the SenFlag signature identified macrophage populations in aging, DNA damage, and injury scRNA-seq datasets. To distinguish macrophage activation from senescence-associated states, we performed complementary analyses.

**Figure 5:**
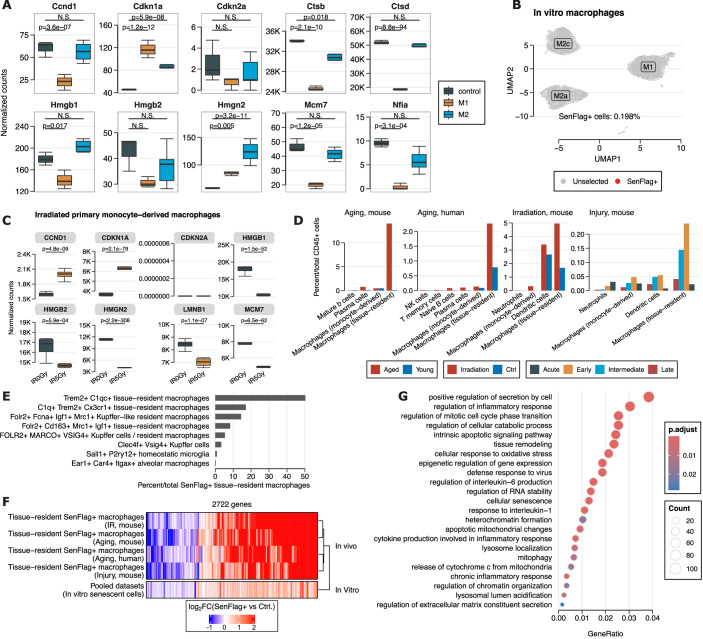
Distinction between macrophage activation and senescence-associated transcriptional states. (**A**) Normalized expression values of indicated genes in in vitro–induced macrophages; control (*n* = 3), M1 (*n* = 3), M2 (*n* = 6). Samples represent murine bone marrow–derived macrophages that were polarized to different activation states. Differential expression and statistical significance were assessed using DESeq2. The center line of the box plot represents the median, the limits indicate the 25th and 75th percentiles, and whiskers represent values within 1.5 × IQR. (**B**) UMAP visualization showing the distribution and proportion of SenFlag+ cells in in vitro-stimulated macrophages. Cells are donor-derived monocytes. (**C**) Normalized expression values of indicated genes in irradiated (IR 5 Gy) and control (IR 0 Gy) macrophages in vitro (*n* = 4). Samples represent primary human monocyte-derived macrophages. *CDKN2A* transcripts were not detected in this dataset. Differential expression and statistical significance were assessed using DESeq2. Box plots are shown in the same format as panel A. (**D**) Distribution of SenFlag+ CD45+ cells across the indicated in vivo conditions. (**E**) Distribution of SenFlag+ tissue-resident macrophage subsets across murine and human aging, irradiation, and injury datasets. (**F**) Heatmap showing transcriptional profiles of SenFlag+ tissue-resident macrophages across in vivo conditions compared to in vitro senescent cells derived from pooled bulk RNA-seq analysis. (**G**) Dot plot showing enriched gene ontology (GO) terms based on genes identified in panel (**F**). Analyses were performed using the clusterProfiler::enrichGO function.

In bulk RNA-seq of monocytes polarized to M1 or M2 states (Chai, 2017), both macrophage populations upregulated *Cdkn1a* after stimulation. However, unlike SenFlag+ cells, they did not induce *Ccnd1* and downregulate *Hmgb1/2/n2*, both of which are core SenFlag features. These results indicate that SenFlag distinguishes senescence-associated states from canonical macrophage activation. Notably, M2 macrophages retained *Nfia* expression, consistent with a quiescent-like state (Fig. 5A).

We next analyzed scRNA-seq of M1- and M2-polarized monocytes (Modak et al, 2022b). Consistent with the bulk data, SenFlag+ cells were nearly absent, comprising only ~0.2% of cells (Fig. 5B). Thus, canonical macrophage activation alone is insufficient to generate a SenFlag+ state.

We then asked whether macrophages driven into senescence acquire a profile resembling other senescent cells. In data of irradiated primary macrophages (Mikhalkevich et al, 2020), we observed a transcriptional profile consistent with the SenFlag signature, including upregulation of *CCND1* and *CDKN1A*, repression of *HMGB1/2/N2*, and reduced expression of proliferation genes such as *MCM7* (Fig. 5C). The results from Fig. 5A,C indicate that activation-associated *Cdkn1a* expression is distinct from bona fide senescence *Cdkn1a* induction.

Finally, we examined which macrophage subtypes SenFlag identifies in vivo. Among CD45+ cells from aging, irradiation, and injury datasets, SenFlag marked distinct tissue-resident macrophage subpopulations (Fig. 5D,E). The SenFlag+ tissue-resident macrophages shared a substantial set of differentially expressed genes with in vitro senescent cells (obtained from the pooled analysis described in Fig. EV1B) relative to matched controls (Fig. 5F). These shared genes were enriched for GO terms related to inflammation, secretion, apoptosis, lysosomal and mitochondrial dysfunction, and tissue remodeling (Fig. 5G).

These results indicate that SenFlag does not broadly capture activated macrophages, but instead identifies distinct macrophage subpopulations with senescence-associated transcriptional features, particularly in contexts associated with increased senescence such as aging, irradiation, and tissue injury.

### Systematic comparison with published senescence signatures

**Figure 6:**
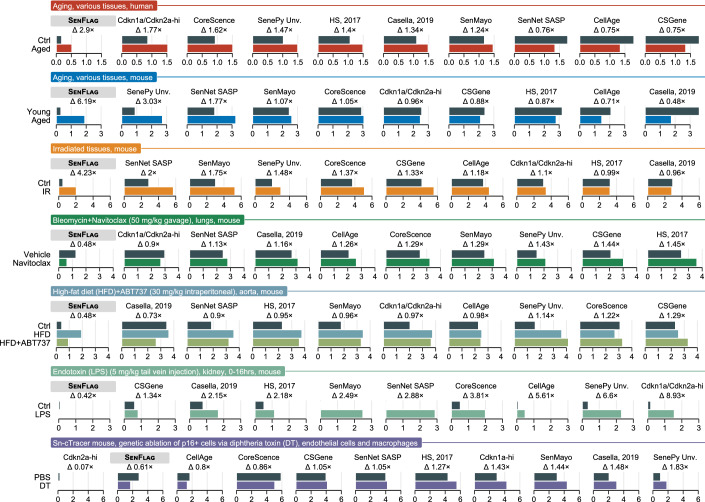
Systematic comparison of SenFlag with published senescence signatures across multiple conditions. Comparison of SenFlag with published senescence-associated gene signatures across aging, DNA damage, senolytic treatment, inflammatory challenge, and genetic ablation datasets. Signatures include SenMayo, CoreScence, SenePy (universal), Hernandez-Segura (2017), Casella (2019), CellAge, CSGene, and a *CDKN1A/CDKN2A*-high classifier. For each condition, the proportion of cells classified as senescent is shown for the indicated signatures. Δ (delta) represents the ratio between the senescence-enriched condition and its corresponding control (e.g., aged versus young, irradiated versus control, or treated versus untreated). Datasets include human and mouse aging atlases, irradiated murine tissues, bleomycin-induced lung injury with Navitoclax treatment, high-fat diet with ABT737 treatment, acute endotoxin (LPS) exposure, and genetic ablation of p16+ cells using diphtheria toxin. Across conditions associated with senescence accumulation (aging and irradiation), SenFlag shows higher condition-to-control ratios compared to most other signatures. In senolytic and genetic ablation datasets, SenFlag+ cells decrease in response to treatments that reduce senescent cell burden. In contrast, several signatures show limited sensitivity to senolysis or increased signal in inflammatory contexts such as acute LPS exposure.

We systematically compared our method against published transcriptomic signatures that have been proposed to identify senescent cells (Fig. 6). These include SenMayo, a cross-tissue panel enriched for inflammatory and extracellular remodeling programs characteristic of the SASP (Saul et al, 2022); CoreScence, a set of genes distilled from recurrent markers across single-cell and spatial studies, capturing mixed secretory and stress-associated features (Qu et al, 2025); and the RNA-seq–derived set of Hernandez-Segura (HS, 2017), built upon bulk RNA-seq data of senescent fibroblasts across diverse triggers and comprising both cell-cycle repression and secretory components (Hernandez-Segura et al, 2017). We further included CSGene and CellAge, two literature-curated resources that aggregate experimentally supported senescence-associated genes and modulators, encompassing heterogeneous mixtures of cell-cycle, stress-response and SASP-related markers (Avelar et al, 2020; Zhao et al, 2016). The Casella (2019) signature, derived from intersecting RNA-seq profiles across fibroblast and endothelial senescence models, was included as a cross-model transcriptomic consensus combining growth-arrest and remodeling programs (Casella et al, 2019). Finally, we evaluated the SenePy universal signature, derived from a large in vivo aging scRNA-seq collection and designed to capture senescence-associated transcriptional shifts recurring across cell types in living tissues (Sanborn et al, 2025).

In addition to these published frameworks, we included two deliberately simplified comparators to model common practice: a *CDKN1A/CDKN2A*-based flagging strategy, simulating the classification of cells as senescent solely on the basis of high *CDKN1A* or *CDKN2A* expression, and a generic SASP panel proposed by the SenNet Consortium (Suryadevara et al, 2024) reflecting canonical inflammatory and matrix-remodeling markers.

Comparisons were performed across datasets shown in the previous figures, including the Tabula Consortium natural aging dataset and multiple irradiated tissues. We also analyzed datasets involving the senolytics Navitoclax (Le Saux et al, 2024) and ABT737 (Mazan-Mamczarz et al, 2025), genetic ablation of p16+ cells (Zhao et al, 2024), and short-term in vivo endotoxin exposure (Janosevic et al, 2021). For scoring-based methods, scores were computed per cell type, and the top 3% of cells per cell type were classified as senescent for each signature.

Across conditions, our method showed robust and reproducible behavior, including natural aging, irradiation-induced senescence, senolytic treatment, and short-term LPS exposure. In contrast, several published signatures were less responsive to Navitoclax- and ABT737-mediated clearance. Moreover, under acute LPS challenge, many approaches classified activated immune populations as senescent-like, consistent with strong reliance on SASP markers, which are not unique to cellular senescence. Although our signature relies heavily on *CDKN1A/CDKN2A*, it provided improved specificity compared to *CDKN1A/CDKN2A*-high selection alone. While it generally identified fewer cells overall than other methods, the relative enrichment between senescence-associated conditions and controls was higher (Δ), indicating improved condition-specific discrimination. This pattern is consistent with preferential detection of more established senescent states rather than transient activation or stress responses. Together, these analyses indicate that SenFlag complements existing signatures by prioritizing core cell-cycle arrest features while reducing sensitivity to inflammatory or transient activation states.

## Discussion

In this study, we introduced a streamlined method for identifying senescent cells in scRNA-seq datasets using cell cycle inhibition as the primary hallmark of senescent cells. Our approach, referred to as SenFlag, was derived from proliferation markers, cell cycle inhibitors, and signatures from bulk RNA-sequencing data of in vitro senescent cells, which include decreased *HMGB1/2/N2* and elevated lysosomal gene expression. We based SenFlag on in vitro senescent cells because they are biochemically validated or induced using defined triggers such as DNA damage, oncogene activation, and replicative exhaustion. This minimizes confounding signals from transient stress and other cellular states that can resemble senescence when signatures are derived directly from in vivo data. By defining a consistent transcriptional signature from a controlled setting, we were able to identify in vivo cells that recapitulate the senescence profile, enabling systematic characterization of senescence across cell types and conditions.

The observed paradoxical upregulation of *CCND1* in multiple senescence-associated datasets is consistent with earlier reports of *CCND1* upregulation in senescent cells (Fukami et al, 1995; Lucibello et al, 1993). This pattern likely reflects the ongoing tension between attempts to re-enter the cell cycle and the active inhibition of the cell cycle by p21 and p16. Notably, *CCND1* expression was associated with conditions of cell-cycle arrest in which *CDKN1A* or *CDKN2A* were active. This supports the interpretation that *CCND1* upregulation reflects a state of cell-cycle arrest occurring in the presence of ongoing cell-cycle signaling. Accordingly, *CCND1* provides a context-dependent marker of cell-cycle arrest when interpreted in combination with *CDKN1A* or *CDKN2A*, rather than as a standalone indicator.

We observed a consistent downregulation of HMG genes, including *HMGB1/2/N2*. While previous studies have reported *HMGB1/2* depletion in senescent cells (Sofiadis et al, 2021; Zirkel et al, 2018), our analysis revealed that the RNA levels of these genes are consistently reduced in senescent cells. These genes are functionally interconnected with other components of our senescence signature. Knockdown experiments demonstrated that the loss of *HMGB1/2* genes induced a signature similar to that of SenFlag, suggesting that *HMGB1/2* downregulation modulates key transcriptomic changes characteristic of senescent cells. Conversely, we acknowledge that alternative chromatin and epigenetic mechanisms, including transposable element derepression or lineage plasticity, may not be fully captured by this framework.

Another notable feature of senescent cells is the upregulation of V-ATPase subunit genes, such as *ATP6V1G1, ATP6V1F*, and *ATP6V1E1*, consistent with prior literature linking senescence to increased lysosomal biogenesis and remodeling (Deng et al, 2024; Kang et al, 2017; Tan and Finkel, 2023). These changes are consistent with impaired lysosomal acidification in senescent cells and may reflect a compensatory mechanism for restoring lysosomal function. Interestingly, we noted that *NFIA* was expressed in multiple quiescent cell populations, including fibroblasts, muscle stem cells, and certain macrophage subtypes. Even though *NFIA* is associated with gliogenesis (Tchieu et al, 2019), our results indicate that its role is not restricted to specific cell types. This broad association suggests that *NFIA* may contribute to the maintenance of a non-proliferative state across diverse tissues, providing a potential marker for distinguishing quiescence from senescence.

Analysis of in vivo senescence revealed several important insights. Senescent cells account for a small fraction of the total cells in aging organisms, generally less than two percent, with endothelial and epithelial cells being the most abundant, consistent with their prevalence in tissues. Fibroblasts, which are frequently used in vitro, display inconsistent patterns. Notably, the triggers of in vivo senescence are not well defined. Most senescent cells appear to lack markers of p53 activation, with TGFβ and MAPK pathways identified as predominant. This observation may reflect the presence of multiple context-dependent induction mechanisms within the senescent cell population that are distinct from canonical DNA damage responses.

Injury-induced senescent cells share canonical features with age-associated senescent cells, including *HMGB1/2/N2* downregulation, *CCND1* upregulation, and *CDKN1A/CDKN2A* positivity, but exhibit variable expression of apoptosis-related genes. Although we cannot directly infer cell fate from scRNA-seq data, the variability in apoptosis-related gene expression reflects differences in persistence and clearance, consistent with the possibility that injury-associated senescent cells are more dynamically regulated compared to senescent cells observed in aging contexts.

Our work addresses the overlap between senescent cells and immune cells. While activated macrophages can upregulate *CDKN1A* and lysosomal markers, this alone does not indicate senescence, as it occurs without *CCND1* induction and downregulation of *HMGB1/2/N2*. Previous studies have shown that macrophages can express canonical senescence-associated markers, such as p16 and SA-β-gal, in non-senescent contexts, and that this expression is reversible and influenced by polarization cues (Hall et al, 2017). Alternatively, p21 itself was shown to be induced downstream of activation cues such as IFNγ and IL-4 (Arpa et al, 2009; Xaus et al, 1999), consistent with our findings (Fig. 5A). Our analyses support a more specific framework for distinguishing senescence-associated states from immune activation requiring reduction of proliferation markers, *CCND1* induction, and *HMGB1/2/N2* downregulation, features already displayed by in vitro senescent macrophages (Fig. 5C). By distinguishing inflammatory activation from senescence, this study helps clarify the distinction between these states and suggests that tissue-resident macrophages may adopt a senescence-associated transcriptional state that could be relevant in the context of aging and senescence-associated chronic diseases. Importantly, SenFlag is designed to capture a minimal and conserved transcriptional framework centered on cell-cycle arrest, and is intended to complement, rather than replace, existing SASP-based or context-specific senescence signatures.

Our approach has limitations: its applicability to snRNA-seq data may be limited, mainly because of the absence of *CCND1* transcripts, which could be a limitation of sequencing depth. In addition, because we strictly filtered out cells with proliferation markers, the analysis primarily captured G1-arrested senescent cells and may have overlooked populations in the S, G2, or M phases. Nevertheless, this simple and effective approach for identifying senescent cells reduces uncertainty about their in vivo profile, supports the presence of conserved core transcriptional features among senescent cells, and enables more accurate senescence-focused studies in disease and aging.

## Methods

**Table:** Reagent and tools table

Reagent/resource	Reference or source	Identifier or catalog number
**Experimental models**		
BJ cells	ATCC	CRL-2522
293FT cells	Invitrogen	R70007
Female C57BL/6 mice		
7 weeks and 88 weeks	C57BL/6	
**Recombinant DNA**		
Codon-optimized human CDKN2A/p16 and CDKN1A/p21 coding region	Integrated DNA Technologies (IDT)	
Tet-on doxycycline-inducible expression lentiviral vector	Addgene	#121919
Third-generation lentivirus packaging plasmids	Addgene	
**Antibodies**		
CCND1 antibody	Cell Signaling Technology	#2978
GAPDH antibody	BioLegend	#607905
p21 antibody for in vitro immunofluorescence	Santa Cruz Biotechnology	sc-6246
p16 antibody for in vitro immunofluorescence	Abcam	ab270058
p16 antibody for in vivo immunofluorescence/FFPE sections	Santa Cruz Biotechnology	sc-1207
HRP-conjugated secondary antibodies	Invitrogen	Various, depending on the target species
ECL Detection kit	Amersham	GERPN2235
**Oligonucleotides and other sequence-based reagents**		
qPCR primer: TUBA1A forward		CTTCGTCTCCGCCATCAG
qPCR primer: TUBA1A reverse		CGTGTTCCAGGCAGTAGAGC
qPCR primer: TMEM199 forward		GCCTTCGTCTGCACTTACCT
qPCR primer: TMEM199 reverse		CCACAGAGGCGACGATCAAT
qPCR primer: CCND1 forward		GACCCCGCAGATTTTCATTG
qPCR primer: CCND1 reverse		ATGGAGGGCGGATTGGAAAT
**Chemicals, enzymes and other reagents**		
Advanced DMEM	Thermo Fisher Scientific/Gibco	#12491015
Fetal bovine serum (FBS)	Thermo Scientific	A4736401
Penicillin-streptomycin	Thermo Fisher Scientific	15140-122
GlutaMAX supplement	Thermo Fisher Scientific/ Gibco	#35050061
Puromycin	Sigma-Aldrich	P8833-10MG
Doxycycline	Sigma-Aldrich	D3447-500MG
PureLink RNA Mini Kit	Invitrogen	#12183018 A
High-Capacity cDNA Reverse Transcription Kit	Applied Biosystems	#4368813
GoTaq qPCR Master Mix / GoTaq reagent used for qPCR	Promega	A6002
RIPA buffer	Abcam	ab156034
Protease and phosphatase inhibitor cocktail	Thermo Fisher Scientific	A32959
BCA protein assay	Thermo Fisher Scientific	23225
Laemmli sample buffer	In-house	
SDS–PAGE gels	Bio-Rad	
Nitrocellulose membranes	Bio-Rad	
TBST	In-house	
Tween	Sigma-Aldrich	P1754-25ML
Formaldehyde	Thermo Fisher Scientific	28908
Hoechst nuclear counterstain	Sigma-Aldrich	B2261-25MG
Phalloidin / F-actin staining reagent	AAT Bioquest	23115
**Software**		
R	R Project; https://www.r-project.org/	v4.4.3
DESeq2	https://pubmed.ncbi.nlm.nih.gov/25516281/; Bioconductor: https://bioconductor.org/packages/DESeq2/	v1.46.0
Seurat	https://pubmed.ncbi.nlm.nih.gov/37231261/; https://satijalab.org/seurat/	v5.3.1.0
Harmony	https://github.com/immunogenomics/harmony	
Kallisto	https://pubmed.ncbi.nlm.nih.gov/27043002/	v0.46.1
clusterProfiler	https://pubmed.ncbi.nlm.nih.gov/34557778/; https://bioconductor.org/packages/clusterProfiler/	v4.14.6
fgsea	https://www.biorxiv.org/content/10.1101/060012v3; https://bioconductor.org/packages/fgsea/	
monocle	https://pubmed.ncbi.nlm.nih.gov/24658644/; https://cole-trapnell-lab.github.io/monocle3/	v3
GEO2R	NCBI GEO; https://www.ncbi.nlm.nih.gov/geo/geo2r/	
PROGENy	https://github.com/saezlab/progeny; https://bioconductor.org/packages/progeny/	
Custom cell type annotation pipeline integrating PanglaoDB with LLM-based literature validation	This study; https://panglaodb.se/	
**Other**		
Sterile 6 mm biopsy punch	KAI medical	
Semi-dry Turbo Transfer system	Bio-Rad	1704271
Amersham imaging system	Cytiva/Amersham	
Ensembl human reference cDNA transcriptome	Ensembl; https://www.ensembl.org/	GRCh38
Ensembl mouse reference cDNA transcriptome	Ensembl; https://www.ensembl.org/	GRCm39
SEPDB secreted protein database	https://pubmed.ncbi.nlm.nih.gov/38345567/	

### Bulk RNA-seq analyses

Bulk RNA-seq datasets were processed using DESeq2 (v1.46.0) (Love et al, 2014) with default parameters. When available, we used processed count matrices from NCBI GEO, either GEO2R or author-supplemented count matrices. For the systematic analysis of bulk RNA-seq data, we relied on uniformly processed matrices from ARCHS4 (Lachmann et al, 2018), which realigned raw FASTQ files using a standardized Kallisto pipeline. For datasets not included in ARCHS4, we either used GEO2R or aligned the raw reads using Kallisto (v0.46.1) (Bray et al, 2016) with default parameters and reference cDNA transcriptomes from *Ensembl* (GRCh38 for humans and GRCm39 for mice).

### Single-cell RNA-seq analyses

Single-cell datasets were analyzed using Seurat (v5.3.1.0) (Hao et al, 2024) using published count matrices from NCBI GEO without reprocessing the raw FASTQ files. Data were processed according to the standard Seurat pipeline, and biological replicates were integrated using Harmony, specifying the sample donor as the layer variable. For cross-condition comparisons, single-cell data were aggregated to pseudobulk profiles using the AggregateExpression function, passing the biological replicate as one of the grouping parameters, and subsequently analyzed using DESeq2.

For analyses involving Tabula data, only tissues with matched young and aged samples were included to ensure balanced representation. From Tabula Muris, we analyzed skin, limb muscle, heart and aorta, spleen, diaphragm, pancreas, thymus, brain (non-myeloid and myeloid), large intestine, brown adipose tissue, mesenteric adipose tissue, subcutaneous adipose tissue, gonadal adipose tissue, lung, marrow, and liver. From Tabula Sapiens, we included blood, bone marrow, fat, large intestine, liver, mammary gland, muscle, ovary, pancreas, skin, small intestine, and uterus.

For PROGENy analysis, we selected non-overlapping senescence-associated pathways from the PROGENy database and chose three unique, highly associated downstream genes per pathway. The selected genes were *GADD45A*, *MDM2*, and *GDF15* for p53; *SPRY2, SPRY4*, and *FOSL1* for MAPK; and *DACT1, SMAD7*, and *PMEPA1* for TGFβ signaling pathway.

For comparative analysis, the Seurat function AddModuleScore was used. For gene sets with separate up- and downregulated lists, these were scored separately and subtracted. For others, a single score was used. The top 3% of cells per cell type with the highest score for each gene set were labeled positive. Scoring was done per cell type to control for baseline differences between the cell types. The abundance of senescent cells was calculated identically across all signatures, including SenFlag.

### Differential expression analyses

Differential expression analyses focused on protein-coding genes and genes with assigned symbols, excluding uncharacterized/predicted loci and genes that showed cell-type specificity as reported by PanglaoDB. The Wald test was used for the DESeq2 pipeline and the Wilcoxon rank-sum test for Seurat. To prioritize biologically relevant results and reduce long gene lists, we relied on padj-weighted log₂ fold change values. For single-cell analyses, we filtered genes based on expression abundance (percentage of cells expressing the gene) to ensure representative results. Significance was defined as *p*_adj_ < 0.05, with stricter thresholds applied when necessary to refine the results. The presented gene symbols follow human nomenclature except when describing mouse data. Differential expression analysis used experiment-specific controls, with no sample sharing across studies. For scRNA-seq secretome analysis (Fig. 3M), we used SEPDB to identify genes that encode secreted proteins (Wang et al, 2024).

### Cell type annotation

We annotated all single-cell data using a custom pipeline that integrates PanglaoDB (Franzén et al, 2019) with an LLM that mines the literature to obtain detailed cluster annotations. First, we identified cluster-specific markers using FindAllMarkers in Seurat. Markers were filtered to retain genes expressed in at least 70% of the cells within each cluster and were statistically significant (*p*_adj_ < 0.0001). Genes were then ranked by both adjusted *p*-value and log₂ fold change, and the top markers for each cluster were used for PanglaoDB annotation. The results from PanglaoDB were then passed to the LLM for validation. Only clusters with unambiguous annotations were used for downstream analyses; clusters showing mixed signals, such as a roughly equal representation of two cell types, or clusters that could not be confidently annotated, were excluded. In some cases, we further distinguished endothelial cells from macrophages, which can have overlapping transcriptomic profiles, using a combination of *PTPRC* (CD45), EDN1, VWF, and CD93 to ensure more accurate classification.

### Functional enrichment analyses

Gene ontology (GO) and pathway enrichment analyses were performed using clusterProfiler (v4.14.6) (Wu et al, 2021). The enrichment results were simplified to remove redundant terms using the simplify function, retaining the most representative biological categories based on semantic similarity and significant enrichment (*p*_ad_ < 0.05). Barcode plots were obtained using the fgsea package.

### Wound-healing experiment

Aged (88 weeks) and young (7 weeks) female C57BL/6 mice were used for the wound-healing experiments in Fig. EV4A. Mice were group-housed in standard cages under controlled environmental conditions with ad libitum access to food and water. Bilateral full-thickness cutaneous wounds were generated on the dorsal skin using a sterile 6 mm biopsy punch under isoflurane anesthesia. Post-surgery, the animals were monitored daily for general health and wound condition. Wound tissues were collected 4 days after injury, fixed, and processed for histological staining. All procedures were performed at the Central Animal Facility of the University Medical Center Groningen following the institutional guidelines for animal welfare. All experimental procedures were approved by the Central Committee for Animal Experiments (CCD:AVD10500202115445) with institutional approval code IVD:2115445-01-001.

### Western blotting

Cell lysates were prepared by sonication in RIPA buffer (Abcam ab156034) supplemented with protease and phosphatase inhibitors (Thermo Fisher Scientific A32959). Lysates were centrifuged at 12,000×*g* for 20 min at 4 °C, and protein concentrations were determined using the BCA assay (Thermo Fisher Scientific 23225). Equal amounts of protein were mixed with Laemmli sample buffer, boiled for 5 min, and resolved on SDS–PAGE gels. Proteins were then transferred onto nitrocellulose membranes using a semi-dry Turbo Transfer system (Bio-Rad 1704271). Membranes were blocked in 5% non-fat dry milk in TBST for 1 h at room temperature and incubated overnight at 4 °C with primary antibodies diluted in blocking buffer (1:1000). After washing, membranes were incubated for 1 h at room temperature with HRP-conjugated secondary antibodies, diluted according to the manufacturer’s instructions. Bands were visualized using enhanced chemiluminescence (ECL) detection (Fisher Scientific RPN2236) and imaged with an Amersham imaging system. For fluorescent Western blotting (GAPDH), Tween was omitted, BSA was used for blocking, and the membrane was directly visualized without secondary antibodies. Antibodies used: CCND1 (Cell Signaling 2978); GAPDH (BioLegend 607905).

### In vitro immunofluorescence staining

Cells were seeded onto glass coverslips and fixed with 4% paraformaldehyde at room temperature. After washing with PBS, cells were permeabilized and blocked in 1–3% BSA in PBS. Coverslips were incubated with primary antibodies diluted in blocking buffer, washed, and then incubated with fluorophore-conjugated secondary antibodies. Nuclei were counterstained with Hoechst, and coverslips were mounted using anti-fade medium. Images were acquired using identical settings for all related conditions. Antibodies used: p21 (1:1000, Santa Cruz Biotech sc-6246); p16 (1:500, Abcam ab270058); Phalloidin (1:1000, AAT Bioquest 23115).

### In vivo immunofluorescence staining

Skin samples were fixed, embedded in paraffin, and sectioned. FFPE sections were deparaffinized, rehydrated, and subjected to antigen retrieval. Sections were incubated with primary p16 antibody sc-1207 (1:150), followed by appropriate secondary antibody and mounting for fluorescence imaging, all prepared according to the manufacturer’s instructions.

### Statistical analysis

Statistical analyses of transcriptomic data were performed using the statistical models implemented in the respective R packages using the default function parameters. Differential expression analysis with DESeq2 was based on negative binomial generalized linear models, with significance assessed using Wald tests. Seurat FindMarkers and FindAllMarkers were run using the default Wilcoxon rank-sum test. Over-representation analysis in clusterProfiler was performed using hypergeometric testing, equivalent to one-sided Fisher’s exact test. Gene set enrichment analysis in clusterProfiler was performed as pre-ranked GSEA using a weighted running-sum enrichment statistic, with the default fgsea backend where applicable. fgseaMultilevel was used for pre-ranked GSEA with a running-sum enrichment statistic and adaptive multilevel split Monte Carlo estimation of *p* values. Pseudotime-associated gene expression was tested using Monocle 3 graph_test, which applies Moran’s I spatial autocorrelation testing on the learned principal graph (Trapnell et al, 2014). Pooled DESeq2 outputs were analyzed using the rma function from the metafor package (Viechtbauer, 2010) with log_2_ fold changes as effect-size estimates and their standard errors as sampling errors. Here, the random-effects models were fitted using restricted maximum likelihood (method = “REML”), with Wald-type z-tests used for model coefficients. All *p* value adjustments were done using the Benjamini–Hochberg method.

For non-sequencing quantitative assays, data were log_2_-transformed prior to analysis. The mean was used as the measure of central tendency where applicable. Group comparisons were assessed using unpaired two-tailed Student’s *t*-tests. All analyses were conducted in R (v4.4.3).

### Senescent cells identification using SenFlag

To identify senescent cells in vivo, we applied the SenFlag framework, a rule-based transcriptional classification implemented on raw count data (Seurat *counts* slot; an example code is provided in Dataset EV1). Cells were classified as SenFlag+ if they satisfied all of the following criteria:

#### Low HMG chromatin-associated gene expression

Cells were required to exhibit low expression of *HMGB2*, *HMGB1*, and *HMGN2* (all AND conditions). For each gene, expression was constrained not to exceed the 75th percentile of its distribution within the analyzed cellular population.

#### Low proliferation marker expression

Cells were required to show low expression of proliferation-associated genes (*NASP*, *TMPO*, *LBR*, *MCM7*, *SMC4*, *CENPF*, *RRM2*, *TOP2A*, *ATAD2*, and *HELLS*; all AND conditions). Additional markers identified in Fig. 1A were included where necessary. For intestinal tissues, *BIRC3*, *MEIS1*, *TIMELESS*, *DNMT1*, *SMC3*, and *SMC2* were additionally incorporated to reduce misclassification of highly proliferative cells.

#### Low quiescence-associated marker expression

To exclude quiescent cells, we required low expression of *NFIA*, using the same percentile-based thresholding strategy.

#### Positive lysosomal/vacuolar gene expression

Cells were required to express at least one V-ATPase subunit (*ATP6V1G1*, *ATP6V1F*, or *ATP6V1E1*) together with at least one cathepsin (*CTSD* or *CTSB*) (i.e., at least one transcript).

#### Cell-cycle inhibitor activation with CCND1 induction

Cells were required to express *CCND1* together with at least one canonical CDK inhibitor (*CDKN1A* or *CDKN2A*). Because *CDKN2A* detection is sequencing-depth dependent, a permissive threshold is recommended (i.e., ≥1 transcript).

This composite signature captures a senescent transcriptional state defined by stable cell-cycle arrest: low proliferation markers, low HMGB1/2/N2 expression, exclusion of quiescence (low *NFIA*), and induction of CDK inhibitors and lysosomal activation.

Senescent cell abundance was quantified as the fraction of SenFlag+ cells relative to the total number of cells within each condition or age group.

### Localization and construct generation with nuclear import/export tags

Human p16 (*CDKN2A*) and p21 (*CDKN1A*) coding regions were codon-optimized using the IDT optimization tool (https://idtdna.com/), tagged with either a C-terminus SV40 monopartite nuclear localization signal or a C-terminus class 1a leucine-rich PKI nuclear export signal, and synthesized by IDT. The synthesized DNA fragments were then cloned into a Tet-on doxycycline-inducible expression lentiviral vector (Addgene: #121919), and particles were produced by co-transfecting the constructs with third-generation lentivirus packaging plasmids in 293FT cells. BJ cells were transduced with the lentiviral particles and selected with puromycin to generate stable cell lines. Cells were maintained in advanced DMEM (#12491015) supplemented with 4% FBS, penicillin-streptomycin, and GlutaMAX (#35050061). The cultures were routinely passaged to prevent overconfluency and were tested to confirm the absence of mycoplasma contamination. Doxycycline was added at a final concentration of 1 µg/mL to induce expression, with the medium refreshed daily in the case of a 1-week treatment.

### RT-qPCR

Cells were lysed directly in culture plates, and total RNA was isolated using the PureLink RNA Mini Kit (#12183018 A). cDNA was synthesized using the cDNA kit from Applied Biosystems (#4368813). qPCR was performed using GoTaq DNA Polymerase (Promega A6002) and gene-specific primers. Relative gene expression was calculated using the ΔΔCt method and normalized to the housekeeping genes (*TMEM199* and *TUBA1A)*. Primer sequences are as follows: *TUBA1A*: Forward primer CTTCGTCTCCGCCATCAG; Reverse primer CGTGTTCCAGGCAGTAGAGC. *TMEM199*: Forward primer GCCTTCGTCTGCACTTACCT; Reverse primer CCACAGAGGCGACGATCAAT. *CCND1*: Forward primer GACCCCGCAGATTTTCATTG; Reverse primer ATGGAGGGCGGATTGGAAAT.

### Data sources

The following datasets were used in this study: Tabula Muris Senis (Consortium, 2018), Tabula Sapiens 2.0 (Consortium and Quake, 2025), Jin K et al (Jin et al, 2025), PRJEB19157 (Hernandez-Segura et al, 2017), FS25533121 (Zhang et al, 2024g), GSE222400 (Suda et al, 2024), GSE117278 (Sturmlechner et al, 2021), GSE75643 (Lenain et al, 2017), GSE117278 (Sturmlechner et al, 2021), GSE179465 (Li et al, 2023), GSE74324 (Tasdemir et al, 2016), GSE109326 (Sack et al, 2018), GSE117444 (Mitra et al, 2018), GSE213323 (Tanke et al, 2024), GSE238255 (Palikyras et al, 2024a), GSE226225 (Wechter et al, 2023), GSE173879 (Evans et al, 2023), GSE244964 (Girolamo et al, 2024), GSE201447 (Paldor et al, 2022), GSE211713 (Curras-Alonso et al, 2023), GSE218449 (Shamseddine et al, 2023), GSE228198 (Horie et al, 2023), GSE255032 (Mills et al, 2025), GSE188432 (Vu et al, 2022), GSE197626 (Xu et al, 2022), GSE201652 (He et al, 2022), GSE217801 (Warwick et al, 2023), GSE227189 (Cortada et al, 2024), GSE232257 (Zhang et al, 2024a), GSE280914 (King et al, 2025), GSE283288 (Modares et al, 2025), GSE285146 (Tian et al, 2025), GSE288443 (Huang et al, 2025), GSE117542 (Chan et al, 2019), GSE155121 (Zeng et al, 2023), GSE205917 (Cheng et al, 2022), GSE208153 (Krup et al, 2023), GSE241463 (Chen et al, 2024), GSE259342 (Azami et al, 2025), GSE272044 (Beckröge et al, 2025), GSE103958 (Das et al, 2018), GSE147693 (Zhao et al, 2020), GSE222510 (Ximerakis et al, 2023), GSE233363 (Wu et al, 2025), GSE290150 (Kerr et al, 2025), GSE247719 (Zhang et al, 2025), GSE244568 (Le Saux et al, 2024), GSE239591 (Mazan-Mamczarz et al, 2025), GSE151658 (Janosevic et al, 2021), GSE199378 (Modak et al, 2022a), GSE145577 (Mikhalkevich et al, 2021), GSE271670 (Zhao et al, 2024), GSE201217 (Kang et al, 2023), GSE219126 (Yun et al, 2023), GSE202032 (Marin et al, 2023), GSE176107 (Morrow et al, 2022), GSE139575 (Sturmlechner et al, 2021), GSE117979 (Sturmlechner et al, 2021), GSE179880 (Wang et al, 2022), GSE169489 (Yagi et al, 2021), GSE247975 (Zhang et al, 2024f), GSE117208 (Guan et al, 2020), GSE233718 (Sinning et al, 2023), GSE210362 (Wang et al, 2022a), GSE229010 (Jachim et al, 2023), GSE278372 (Zhang et al, 2024b), GSE238254 (Palikyras et al, 2024a), GSE238252 (Palikyras et al, 2024a), GSE245147 (Zhang et al, 2024e), GSE222400 (Suda et al, 2024), GSE230358 (Savić et al, 2023), GSE230181 (Savić et al, 2023), GSE224071 (McHugh et al, 2023), GSE160273 (Yu et al, 2023), GSE234417 (Gulen et al, 2023), GSE165406 (Vannier et al, 2021), GSE180406 (Rey-Millet et al, 2023), GSE184892 (Muto et al, 2023), GSE212085 (Marin et al, 2023), GSE198396 (Wang et al, 2022b), GSE118583 (Sturmlechner et al, 2021), GSE176199 (Liu et al, 2021), GSE165532 (Lee et al, 2021a), GSE176324 (Brawerman et al, 2022), GSE171780 (Sofiadis et al, 2021), GSE155371 (Schwartz et al, 2021), GSE168994 (Lee et al, 2021b), GSE166059 (DePianto et al, 2021), GSE166035 (DePianto et al, 2021), GSE144752 (Montes et al, 2021), GSE153921 (Innes et al, 2021), GSE157867 (Zhang et al, 2021), GSE124609 (Sabath et al, 2020), GSE160702 (Deryabin et al, 2021), GSE132370 (Vizioli et al, 2020), GSE130099 (Chan et al, 2020), GSE130306 (Sati et al, 2020), GSE132204 (Frausto et al, 2020), GSE133292 (Zhang et al, 2021), GSE108278 (Lau et al, 2019), GSE105937 (Sen et al, 2019), GSE109700 (De Cecco et al, 2019), GSE101750 (Georgilis et al, 2018), GSE103938 (Aarts et al, 2017), GSE85082 (Muniz et al, 2017), GSE76605 (Lenain et al, 2017), GSE75643 (Lenain et al, 2017), GSE99028 (Dou et al, 2017), GSE84694 (Yang et al, 2017), GSE94280 (Lizardo et al, 2017), GSE94395 (Baar et al, 2017), GSE63577 (Marthandan et al, 2015), GSE61130 (Herranz et al, 2015), GSE59966 (Hänzelmann et al, 2015), GSE60340 (Purcell et al, 2014), GSE58910 (Crowe et al, 2016), GSE53356 (Rai et al, 2014), GSE56293 (Alspach et al, 2014), GSE207816 (Patra et al, 2024), GSE250224 (Numa et al, 2024), GSE275256 (Armanville et al, 2025), GSE287646 (Kondratyeva et al, 2025), GSE294733 (Belakova et al, 2025), GSE268487 (Dalgarno et al, 2025), GSE264316 (Jiang et al, 2025), GSE280050 (Atlante et al, 2025), GSE226005 (Meng et al, 2025), GSE160279 (Yan et al, 2021), GSE218684 (Gallage et al, 2024), GSE218683 (Gallage et al, 2024), GSE247607 (Etoh et al, 2024), GSE182731 (Haj et al, 2025), GSE221104 (Anerillas et al, 2023), GSE155903 (Guerrero et al, 2022), GSE280365 (Kelsey et al, 2025), GSE281894 (Hu et al, 2025), GSE274295 (Yang et al, 2025), GSE295083 (Zhang et al, 2026), GSE251814 (Neherin et al, 2026), GSE246073 (Kim et al, 2024), GSE279349 (Zhang et al, 2024b), GSE269856 (Palikyras et al, 2024b), GSE245045 (Scheibye-Knudsen et al, 2023), GSE125632 (Fernandez-Rebollo et al, 2019), GSE155680 (Katsuumi et al, 2020), GSE236063 (Frazel and Liddelow, 2023), GSE128420 (Sun and Xu, 2019), GSE296209 (Fu, 2025), GSE235768 (Skea et al, 2023), GSE156472 (Sun and Han, 2020b), GSE156184 (Sun and Han, 2020a), GSE156038 (Sun and Zhang, 2020), GSE214409 (Dou et al, 2022), GSE180361 (Olan and Narita, 2021), GSE252675 (Zheng et al, 2024), GSE216842 (Sun, 2022).

## Supplementary information

Peer Review File

Dataset EV1

Source data Fig. 1

Figure EV1 Source Data

Figure EV4 Source Data

Expanded View Figures

## Data Availability

No new sequencing data were generated in this study. The R scripts developed for this work are provided with the manuscript in Dataset EV1, including an example code of how to use SenFlag. Requests for other materials should be directed to the corresponding author. The source data of this paper are collected in the following database record: biostudies:S-SCDT-10_1038-S44318-026-00845-6.
