# Supplementary material for: SenFlag gene signature identifies senescent cells in mouse and human tissues through a conserved core transcriptional program
Source: EMBO J. 2026 Jul 6;45(15):5580–606. doi: 10.1038/s44318-026-00845-6 (PMC13433995; doi:10.1038/s44318-026-00845-6)
Supplement: Supplementary file 1 [file 44318_2026_845_MOESM1_ESM.pdf]

THE EMBO JOURNAL

SenFlag gene signature identifies senescent cells in mouse and human tissues through a conserved core transcriptional program

Abdullah Altulea, Sebastian Mackedenski, Jamil Nehme, and Marco Demaria

Corresponding author: Marco Demaria (m.demaria@umcg.nl)

Review Timeline:

Submission Date:	16th Jan 26
Editorial Decision:	26th Feb 26
Appeal:	7th Apr 26
Editorial Decision:	19th May 26
Revision Received:	27th May 26
Accepted:	16th Jun 26

Editor: Daniel Klimmeck

Transaction Report:

(Note: With the exception of the correction of typographical or spelling errors that could be a source of ambiguity, letters and reports are not edited. Depending on transfer agreements, referee reports obtained elsewhere may or may not be included in this compilation. Referee reports are anonymous unless the Referee chooses to sign their reports.)

Dear Dr Marco Demaria,

Thank you again for transferring your manuscript EMBOJ-2026-123584-T for consideration by the EMBO Journal. Your study was sent to three reviewers with expertise in cellular senescence biology and related biomarkers, and we have now received reports from all of them which I enclose below. In light of the experts' comments, I am afraid we decided that we cannot offer publication in The EMBO Journal.

As you will see the referees appreciate your potential value of the SenFlag signature, which as to their assessment stands out with a number of features over existing senescence identification paradigms. However, they also point to major issues with the analysis that in our view preclude publication of the study at the EMBO Journal. In more detail, referee #1 states substantial concerns regarding potential cell-type specific bias of SenFlag, that dampen his-her enthusiasm (ref#1, pt. 6). Reviewer #2 in turn states important limitations of the analysis including insufficient benchmarking and demonstration of in vivo sensitivity and specificity as well as lack of conclusive cross-integration with existing SASP-based signatures (ref#2, pts. 1-3,5; see also cross-comment Ref#3). There are also issues raised regarding data discrepancies in the injury-stage patterns of SenFlag+ cells.

These are major issues in our view, and in light of these overall critical opinions and the fact that the EMBO Journal can only afford to accept papers which receive enthusiastic support from a majority of referees, and in light of the one major round of revision we consider as a policy for our venue, I am afraid we concluded that we hence cannot reasonably offer to proceed with peer-review here.

Please note that given the interest stated we would be able to consider a substantially bolstered version of the study editorially; however, due to the extensive experimentation required the outcome of such re-evaluation would be entirely open at this stage.

Thank you in any case for the opportunity to consider this manuscript. I am sorry we cannot be more positive on this occasion, but hope nevertheless that you will find the referees' comments helpful.

Best regards,

Daniel Klimmeck

Daniel Klimmeck, PhD
Senior Editor
The EMBO Journal

Referee #1:

This is a simple study with a potential powerful message. However, I am not fully convinced about how general is this signature (SenFlag) to non-fibroblast cells. See point 6 as my main criticism.

1. The first sentence of Results would benefit from rewriting. It took me a while to understand it and still there are conditions I don't understand: the p53, p16, p21 knockdowns are just fibroblasts with these genes knock-down? How was quiescence induced? The concept of quiescence includes sometimes serum starvation, others confluence. These are very different types of arrest. In vivo cells are quiescent probably because of confluence (which is a mechano-neutral situation with YAP off).

2. Fig. 1E contains many data from many different cell types undergoing senescence. I wonder if the patterns are similar when the analyses are done separately for fibroblasts or for epithelial cells.

3. Regarding this sentence: "CCND1 overexpression in epithelial cells [30] increased the expression of TOP2A, MKI67, MCM7, SMC4, MCM3, and HMGB1/2 but did not induce senescence/SASP genes (Fig. 1H)". Then, is the conclusion that high CCND1 is not a causal of senescence for epithelial cells, but a marker? Shouldn't this be highlighted?

4. The data in Fig. 2A is from in vivo and in vitro experiments? It would be worth mentioning this.

5. Regarding this sentence: "These results confirm that the senescence transcriptome can be distilled into a compact core defined by cell cycle arrest and chromatin loss, ... ". Please be more specific when saying "chromatin loss".

6. My main problem with Fig. 3 is the following: a signature of senescence defined using fibroblasts is applied to all kinds of cells. It would be more cautious to say that a "fibroblast-type senescence" was detected in epithelial and endothelial cells in vivo, but not in macrophages. If senescence had been defined using a different cell type, the markers would be different and when the, let´s say "macrophage-type senescence" markers or "astrocyte-type senescence" markers were applied to other cells, perhaps fibroblasts are very low in these other types of senescence. To me this is a major bias of the paper: applying a fibroblast definition to non-fibroblast cells and conclude that those that don't fit the fibroblast-centered definition are not senescent.

7. If I understand correctly, injury induced senescence (SenFlag+) contains reparative programs in the case of injured tissues undergoing repair. In this case, macrophages are the major senescent population (I guess SenFlag+? Please, clarify; and I guess NFIA low, please clarify).

8. Line 213: It is said that the SenFlag+ senescence with regenerative profile is primed to apoptosis (transient), whereas the SenFlag+ senescence of aging is inflammatory. Where has this been described in the paper? Nothing was mentioned before about inflammation.

Referee #2:

This manuscript proposes "SenFlag," a transcriptional gene set intended to identify senescent cells in scRNA-seq datasets based primarily on coordinated cell-cycle arrest features, HMG loss, CCND1 upregulation, and lysosomal gene induction. The effort to define a mechanistically grounded and compact signature is commendable. However, several conceptual and validation issues limit the current strength of the conclusions.

Below I outline my main concerns.

Major Concerns

1. Lack of Benchmarking Against Established Senescence Signatures

A central limitation of the manuscript is the absence of systematic benchmarking of SenFlag against previously established senescence gene sets, particularly SASP-focused signatures such as SenMayo (Saul et al., Nat Commun 2022) and related frameworks.

Specifically, how does SenFlag perform relative to SenMayo in the same datasets? What is the overlap between SenFlag-positive and SenMayo-positive cells? Does SenFlag capture a distinct senescent subpopulation or largely overlap with existing classifiers? What is gained (specificity? mechanistic grounding? lower false positives?) and what is lost (e.g., inflammatory components)?

A direct, side-by-side comparison in the same in vivo scRNA-seq datasets would substantially strengthen the manuscript. Without such benchmarking, it is difficult to assess whether SenFlag represents an advance over existing approaches or simply a differently parameterized classifier.

2. In Vivo Sensitivity and Specificity Remain Insufficiently Demonstrated

SenFlag is derived largely from in vitro datasets and then applied to in vivo aging and injury models. While increased SenFlag-positive cells are observed with age and after injury, this does not sufficiently establish specificity for bona fide senescence versus broader aging-associated transcriptional changes.

Validation in genetic or pharmacological senescent cell clearance models would greatly strengthen the manuscript. Demonstrating that SenFlag detects both accumulation and reduction of senescent cells would provide critical support for its specificity.

3. Regenerative Senescence: Lack of Temporal Resolution

The authors report that regenerative injury models show increased SenFlag-positive cells (~6-fold). However, in classical models (e.g., wound healing), senescence is considered transient. If SenFlag detects functional senescence, one would expect dynamic changes over time. Therefore, I am puzzled why is there no clear difference in senescence load between early and late stages of wound healing (Figure 4C)?

4. CCND1 Upregulation and Nuclear Localization of p16/p21

The reported correlation between CCND1 upregulation and nuclear localization of p16/p21 is conceptually interesting and potentially important. However, nuclear localization cannot be assessed in scRNA-seq datasets. It therefore remains unclear how this mechanistic insight can be operationalized in purely transcriptomic analyses.

If CCND1 is intended to function as a surrogate marker for nuclear CDKi-mediated cell cycle arrest, the authors should clarify what additional information is provided by retaining CDKN1A and CDKN2A within the SenFlag signature. Would a combination

of CCND1, low proliferation markers, and HMG loss be sufficient to define the classifier?
Conversely, if CDKN1A and CDKN2A are required components, the manuscript should more clearly articulate their non-redundant contribution relative to CCND1. This point is particularly important in light of recent work demonstrating substantial heterogeneity and limited overlap between $p21^+$ and $p16^+$ senescent populations in vivo (PMID: 41162753). Given this reported divergence, it is essential to clarify how SenFlag reconciles or integrates distinct CDKi-positive senescent states.

5. Limited Inclusion of SASP Components
This is going back to my first comment about benchmarking, given that inflammatory remodeling is a hallmark of senescence, it is surprising that SenFlag includes very few canonical SASP factors. The rationale for excluding many SASP genes requires clearer justification. While the authors argue for a mechanistic focus on cell-cycle arrest, inflammatory signaling is a defining biological and pathological feature of senescence in aging tissues. Beyond comparing side by side with SenMayo or other signature focusing on SASP factors, I am also wondering would combining SenFlag with a SASP-focused signature (e.g., SenMayo) improve specificity and biological interpretability?

6. The manuscript presents seemingly conflicting interpretations regarding macrophages, at times suggesting they fail SenFlag criteria (Line 160-163) and at other times describing them as a major senescent population in injury (Line 189-190, Figure 4D). This point requires clarification.

7. The manuscript would benefit from more comprehensive citation of prior work. For example, it is well reported the complex or paradoxical role of CCND1 in senescence (PMID: PMID: 8569279, https://www.biorxiv.org/content/10.1101/2025.08.01.668243v1). Besides, previous efforts to derive transcriptomic senescence signatures should be systemically discussed.

Referee #3:

Summary
Altulea and colleagues present SenFlag, a novel gene set designed to identify senescent cells based on downregulation of proliferation, chromatin architecture and quiescence genes and upregulation of cell-cycle inhibitors, CCND1 and lysosomal genes. Using this signature, they show how senescent cells accumulate in vivo in both humans and mice and identify the main cell types involved, notably endothelial and epithelial cells. This represents an important advance, as it at least partially addresses a major limitation of existing senescence gene sets, which often rely heavily on pro-inflammatory genes that can also be expressed in non-senescent contexts and vary depending on the inducer or the tissue involved.

1- Authors should discuss the success of eliminating CDKN2A+ cells in the light of their finding that this gene was rarely expressed in the population of senescent cells identified by SenFlag.
2- Lines 211-214 state that injury-associated senescence is a transient state. However, the manuscript does not provide clear data to support this claim. While the reported correlation with increased expression of apoptotic genes may suggest that these cells eventually die, this is not directly demonstrated. Senescent cells could also be cleared by the immune system, but the senescent state itself might still be long-lasting; the current data do not distinguish between these possibilities.
3- In the discussion, lanes 224-231, the authors should acknowledge early work linking Cyclin D1 to senescence (1, 2).
4- Although the authors identify a clear link to chromatin architecture through downregulation of HMGB1 and HMGN2, it is possible that SenFlag may miss senescent cells in which chromatin is dysregulated through other mechanisms, see, for example, (3) for a recent review. In addition, SenFlag does not explicitly capture transcriptomic consequences of epigenetic dysregulation such as derepression of repetitive elements, endogenous retroviruses, or increased lineage promiscuity.

1. Pagano M, Theodoras AM, Tam SW, Draetta GF. Cyclin D1-mediated inhibition of repair and replicative DNA synthesis in human fibroblasts. Genes Dev. 1994;8:1627-39.
2. Atadja P, Wong H, Veillete C, Riabowol K. Overexpression of cyclin D1 blocks proliferation of normal diploid fibroblasts. Exp Cell Res. 1995;217:205-16.
3. Lopes-Paciencia S, Ferbeyre G. Increased chromatin accessibility underpins senescence. FEBS J. 2025;292:4112-32.

Referee #3, additional cross-comments:

I also agree with my colleagues' comments. My enthusiasm for this paper stems from the fact that it proposes an alternative gene set which, given the substantial heterogeneity we are uncovering in senescence programs, may prove useful in certain contexts. SenFlag appears to emphasize cell-cycle arrest and chromatin, whereas most other signatures are more heavily driven by SASP-related genes.

That said, the authors will need to address all comments convincingly to support the validity and appropriate use of their signature. For me, the most important point is to obtain practical validation in relevant in vivo models-ideally demonstrating that interventions known to improve outcomes by clearing senescent cells also reduce the burden of SenFlag-positive cells.

** As a service to authors, EMBO Press provides authors with the possibility to transfer a manuscript that one journal cannot offer to publish to another EMBO publication or the open access journal Life Science Alliance launched in partnership between EMBO Press, Rockefeller University Press and Cold Spring Harbor Laboratory Press. The full manuscript and if applicable, reviewers' reports, are automatically sent to the receiving journal to allow for fast handling and a prompt decision on your manuscript. For more details of this service, and to transfer your manuscript please click on Link Not Available. **

Please do not share this URL as it will give anyone who clicks it access to your account.

Reviewer #1:

This is a simple study with a potential powerful message. However, I am not fully convinced about how general is this signature (SenFlag) to non-fibroblast cells. See point 6 as my main criticism.

1. The first sentence of Results would benefit from rewriting. It took me a while to understand it and still there are conditions I don't understand: the p53, p16, p21 knockdowns are just fibroblasts with these genes knock-down? How was quiescence induced? The concept of quiescence includes sometimes serum starvation, others confluence. These are very different types of arrest. In vivo cells are quiescent probably because of confluence (which is a mechano-neutral situation with YAP off).

AUTHORS: We thank the reviewer for this important point and agree that the original text lacked sufficient clarity. We have revised the opening paragraph of the Results section to explicitly describe the experimental conditions and improve readability. In particular, the p53, p16, and p21 perturbation experiments refer to fibroblasts in which these genes were first knocked down, followed by induction of senescence through oncogenic HRAS (OIS). Quiescence was induced by serum deprivation, and this is now clearly stated in the revised manuscript.

We also agree with the reviewer that quiescence is a heterogeneous state and can arise through distinct mechanisms, including serum deprivation and contact inhibition. To address this, and to clarify the role of NFIA in distinguishing quiescence from senescence, we performed additional analyses using datasets of contact inhibition in both fibroblasts and endothelial cells (Revised Manuscript Fig. S1A). These analyses show that contact inhibition, similar to serum deprivation, induces significant upregulation of NFIA (padj < 0.05) while CCND1 is downregulated. Together with the *ex vivo* muscle stem cell data (Revised Manuscript Fig. S2C), these results demonstrate that NFIA induction is a consistent feature of quiescence across distinct biological contexts and is not dependent on the specific mode of quiescence induction. This supports its use as a robust quiescence-exclusion marker within the SenFlag signature.

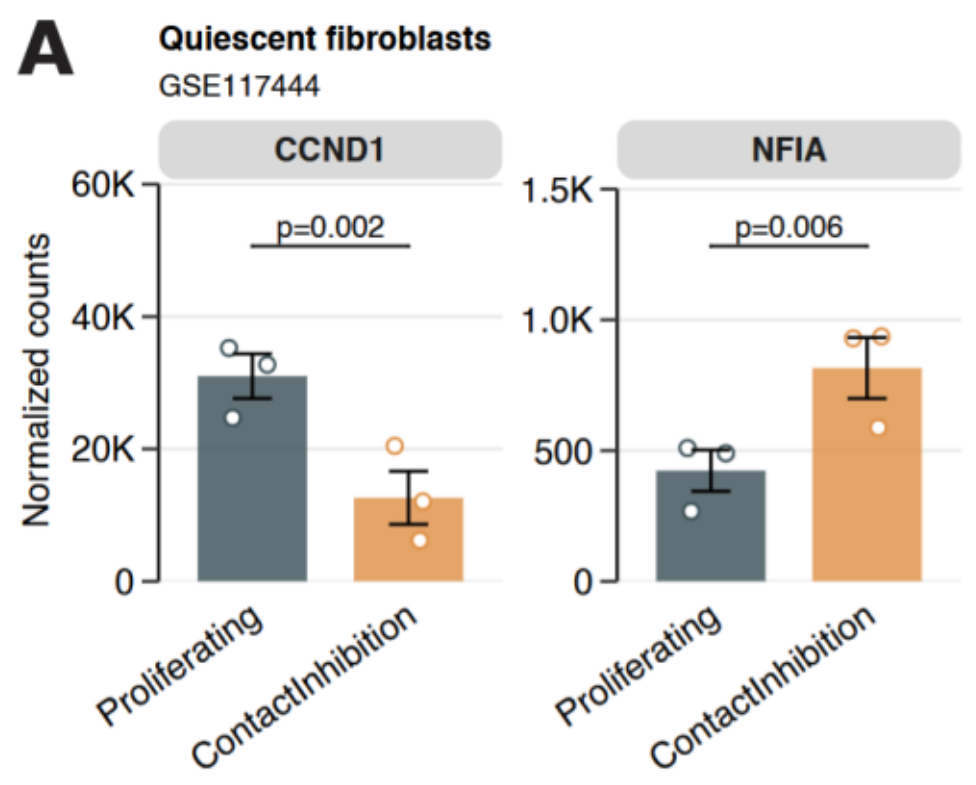

Revised Manuscript Fig. S1A

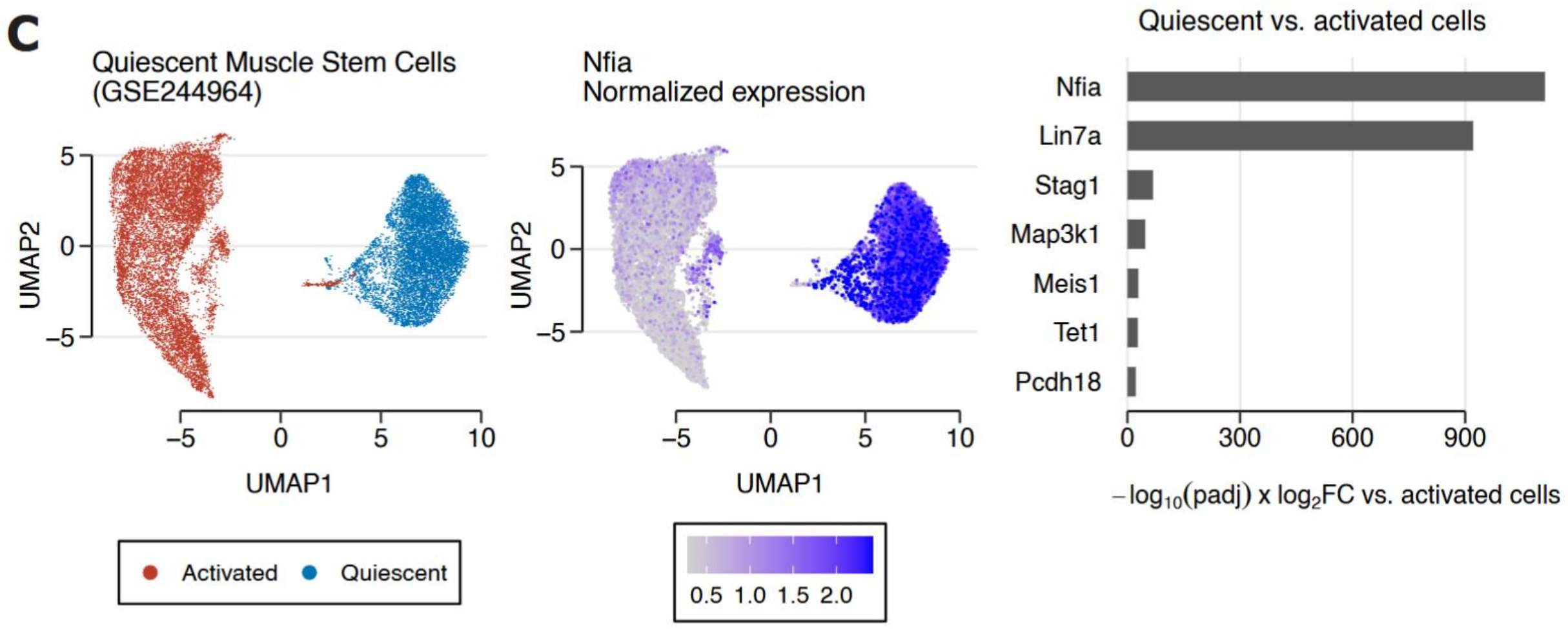

Revised Manuscript Fig. S2C

2. Fig. 1E contains many data from many different cell types undergoing senescence. I wonder if the patterns are similar when the analyses are done separately for fibroblasts or for epithelial cells.

AUTHORS: We agree that the potential cell-type specificity of the SenFlag signature is a key point. To directly address this, we re-analyzed the pooled bulk RNA-seq datasets by assigning cell-type identity to each sample and stratifying the analysis accordingly (Revised Manuscript Fig. S1C). This revealed that the core SenFlag gene expression patterns, including downregulation of *HMGB1/2/N2* and proliferation-associated genes, and upregulation of *CCND1*, are conserved across major cell types, including fibroblasts and epithelial cells. Importantly, to minimize confounding by intrinsic cell-type–specific transcriptional programs, genes with strong cell-type specificity (as defined using PanglaoDB annotations) were excluded at early stages of the analysis. This design ensured that the resulting signature captures shared features of senescence rather than lineage-restricted expression patterns.

Together, these results support the conclusion that SenFlag reflects a conserved transcriptional program of senescence that is largely independent of cell type.

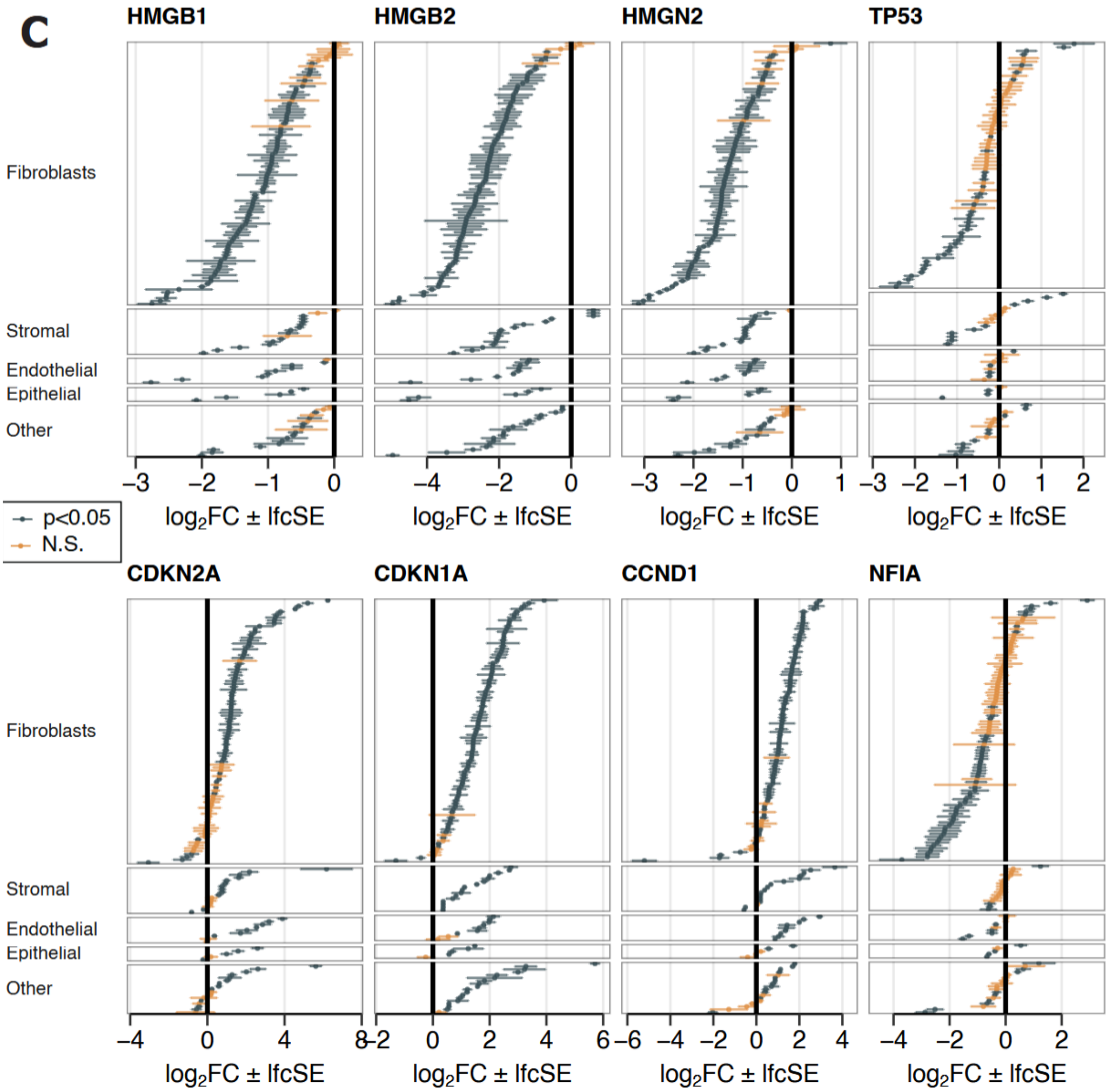

Revised Manuscript Fig. S1C

3. Regarding this sentence: "CCND1 overexpression in epithelial cells [30] increased the expression of TOP2A, MKI67, MCM7, SMC4, MCM3, and HMGB1/2 but did not induce senescence/SASP genes (Fig. 1H)". Then, is the conclusion that high CCND1 is not a causal of senescence for epithelial cells, but a marker? Shouldn't this be highlighted?

AUTHORS: We definitely agree that this distinction should be made more explicit. Our intention was to demonstrate that *CCND1* upregulation is not sufficient to induce a senescence-associated transcriptional program. As shown in Fig. 1H, *CCND1* overexpression in epithelial cells promotes the expression of proliferation-associated genes but does not induce senescence or SASP-related genes, indicating that *CCND1* does not act as a causal driver of senescence in this context. Instead, our data support a model in which *CCND1* functions as a context-dependent marker. In proliferating cells, *CCND1* reflects its canonical role in promoting cell cycle progression. In contrast, in senescent cells, *CCND1* upregulation occurs in conjunction with *CDKN1A* or *CDKN2A* expression and reflects a state

of enforced cell cycle arrest coupled with persistent pro-proliferative signaling. Accordingly, within the SenFlag signature, *CCND1* is not used as a standalone marker but as part of a combinatorial signature requiring concurrent CDK inhibitor activation. We have revised the manuscript text to clarify this point and to explicitly emphasize that *CCND1* is not a causal determinant of senescence, but a contextual readout of cell cycle dynamics under arrest conditions.

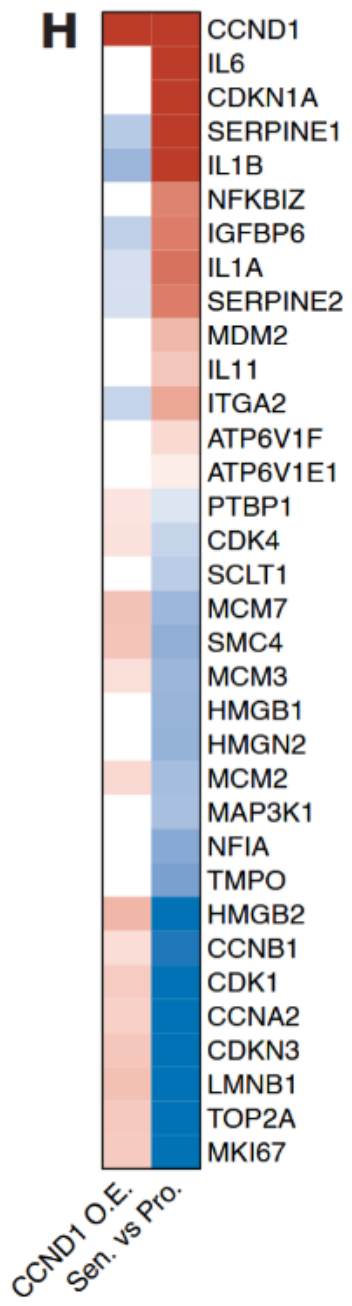

Fig. 1H

4. The data in Fig. 2A is from in vivo and in vitro experiments? It would be worth mentioning this.

AUTHORS: We thank the reviewer for pointing out this lack of clarity. The datasets used in Fig. 2A are derived exclusively from in vitro senescence models. To avoid confusion, we have revised both the main text and the figure legend to explicitly indicate that these analyses are based on in vitro senescent cells.

5. Regarding this sentence: "These results confirm that the senescence transcriptome can be distilled into a compact core defined by cell cycle arrest and chromatin loss, ... ". Please be more specific when saying "chromatin loss".

AUTHORS: We thank the reviewer for highlighting this ambiguity. We agree that the term “chromatin loss” was imprecise in this context. Our intention was to refer specifically to the consistent downregulation of chromatin-associated high mobility group (HMG) proteins, including *HMGB1*, *HMGB2*, and *HMGN2*, observed across senescence models. We have now revised the manuscript to explicitly refer to “loss of HMG chromatin-associated proteins

at the transcript level" rather than "chromatin loss," to more accurately reflect the nature of the data and avoid overinterpretation beyond transcriptomic evidence.

6. My main problem with Fig. 3 is the following: a signature of senescence defined using fibroblasts is applied to all kinds of cells. It would be more cautious to say that a "fibroblast-type senescence" was detected in epithelial and endothelial cells in vivo, but not in macrophages. If senescence had been defined using a different cell type, the markers would be different and when the, let´s say "macrophage-type senescence" markers or "astrocyte-type senescence" markers were applied to other cells, perhaps fibroblasts are very low in these other types of senescence. To me this is a major bias of the paper: applying a fibroblast definition to non-fibroblast cells and conclude that those that don't fit the fibroblast-centered definition are not senescent.

AUTHORS: We thank the reviewer for this important and thoughtful comment. We fully agree that the potential for cell type–specific definitions of senescence is a critical issue, particularly in the context of single-cell analyses. Importantly, SenFlag was not derived from fibroblasts alone, but from a combination of multiple senescence models and datasets, including diverse induction methods and both bulk and single-cell data. The signature was intentionally designed to capture a minimal set of core features of senescence, namely stable cell-cycle arrest (low proliferation markers together with *CDKN1A/CDKN2A* expression), loss of HMG chromatin-associated proteins, and lysosomal activation, rather than cell type–specific transcriptional programs. To directly address the concern of fibroblast bias, we performed additional stratified analyses (Revised Manuscript Fig. S1C), demonstrating that the core SenFlag features are conserved across major cell types, including epithelial and endothelial cells. Furthermore, in vitro analyses across multiple senescence-inducing conditions (Fig. 2) show consistent co-regulation of these features at single-cell resolution, supporting their generality beyond a single lineage. With respect to macrophages, our revised analyses (Revised Manuscript Fig. 5) indicate that SenFlag does not systematically exclude this lineage. Rather, activated macrophages frequently express *CDKN1A* without fulfilling additional SenFlag criteria, such as *CCND1* induction and HMG downregulation. In contrast, macrophages subjected to DNA damage (irradiation) do exhibit a SenFlag profile, indicating that bona fide macrophage senescence aligns with the core transcriptional features identified here. We agree that distinct cell types may exhibit additional, context-specific senescence programs. However, our goal was to define a minimal and conserved transcriptional signature that can be applied across heterogeneous datasets, rather than to capture all lineage-specific manifestations of senescence. We have revised the manuscript to clarify this conceptual distinction and to explicitly state that SenFlag identifies a core senescence state that may be complemented by cell type–specific features.

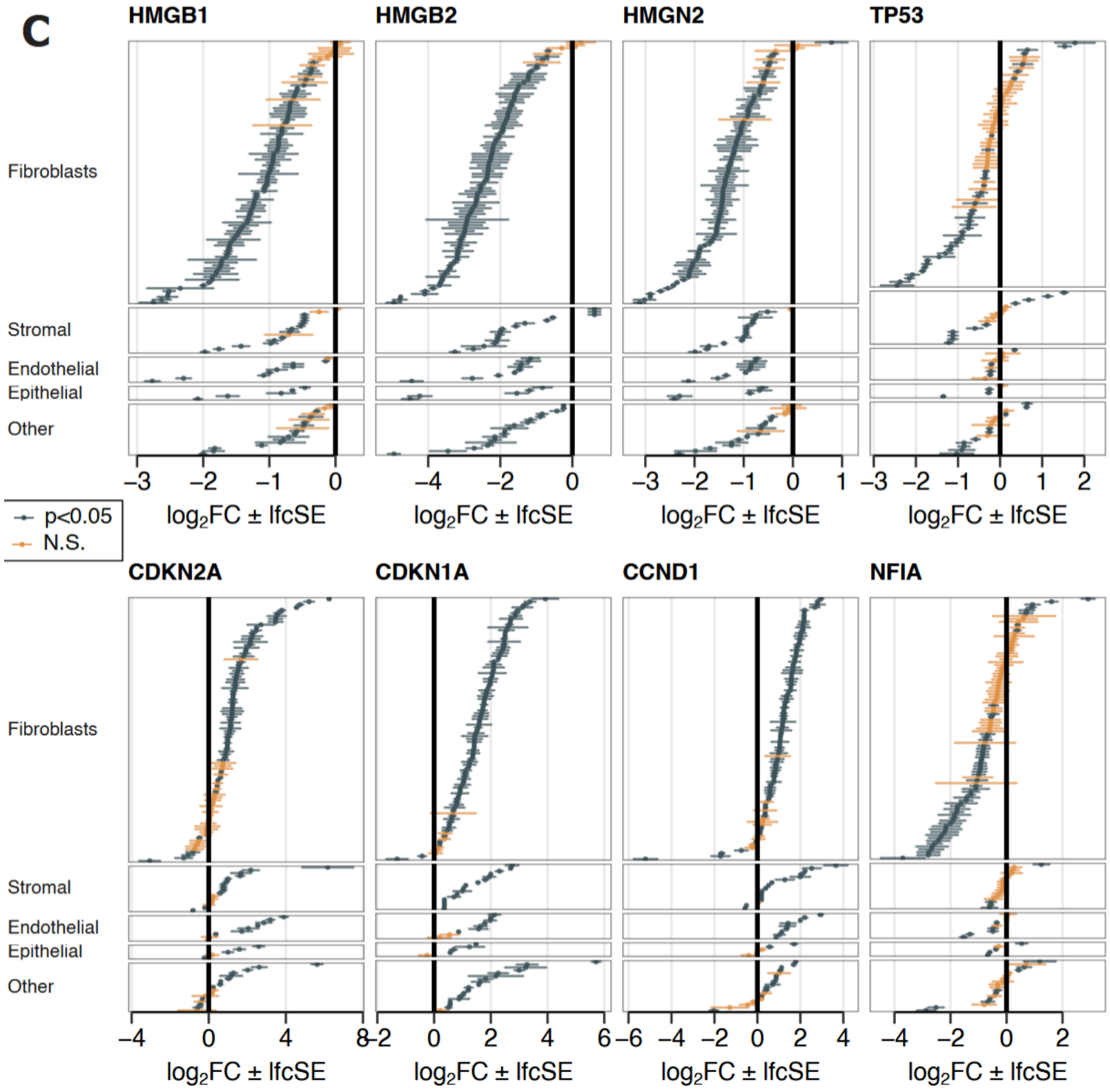

Revised Manuscript Fig. S1C

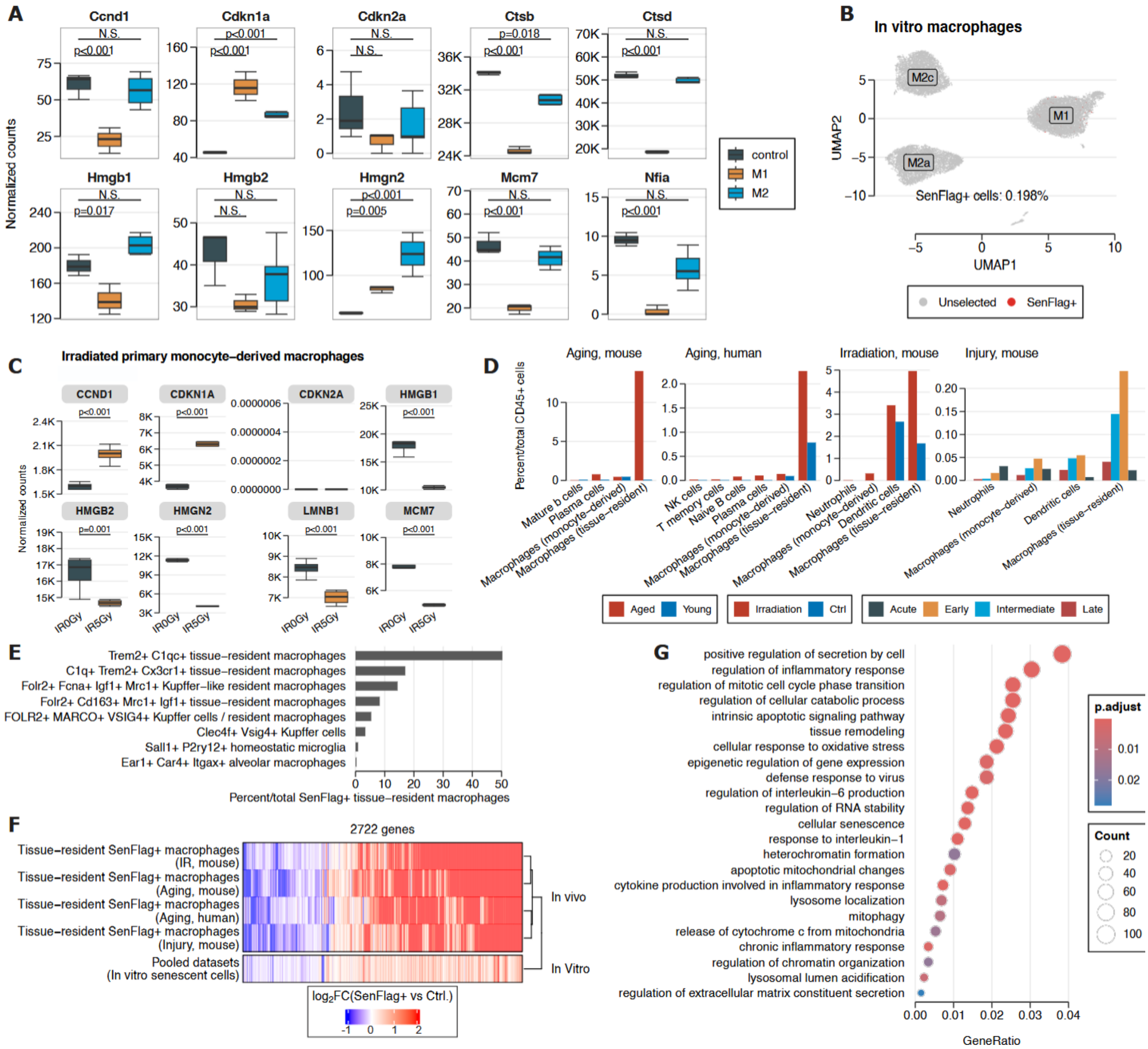

Revised Manuscript Fig. 5

7. If I understand correctly, injury induced senescence (SenFlag+) contains reparative programs in the case of injured tissues undergoing repair. In this case, macrophages are the major senescent population (I guess SenFlag+? Please, clarify; and I guess NFIA low, please clarify).

AUTHORS: We agree that this aspect required clarification. In injury-associated datasets, macrophages indeed represent a major fraction of SenFlag-positive cells. These SenFlag+ macrophages fulfill all core criteria of the signature, including low proliferation markers, low *HMGB1/2/N2*, low *NFIA*, and positive expression of *CCND1* together with *CDKN1A* or *CDKN2A*. We have clarified this explicitly in the revised manuscript. Importantly, our additional analyses (Revised Manuscript Fig. 5, shown above) indicate that SenFlag does not broadly classify all macrophages as senescent. Rather, it distinguishes a specific subset corresponding to tissue-resident macrophages, while excluding activated monocytes, which

typically express *CDKN1A* but lack *CCND1* induction and do not exhibit HMG downregulation.

Notably, SenFlag+ tissue-resident macrophages share extensive transcriptional similarity with in vitro senescent cells (>2,500 overlapping genes; Revised Manuscript Fig. 5F–G), supporting the interpretation that these cells adopt a senescence-like transcriptional state. However, we agree that the functional role of these cells in tissue repair and aging remains to be determined, and we have revised the corresponding statements in the revised manuscript. Finally, we note that the relative abundance of macrophages across datasets may be influenced by differences in tissue pre-processing and selective cell enrichment strategies prior to performing scRNA-seq. We have added a statement in the manuscript to acknowledge this potential source of bias.

8. Line 213: It is said that the SenFlag+ senescence with regenerative profile is primed to apoptosis (transient), whereas the SenFlag+ senescence of aging is inflammatory. Where has this been described in the paper? Nothing was mentioned before about inflammation.

AUTHORS: We thank the reviewer for highlighting this inconsistency. We agree that the original statement was not sufficiently supported in the main text and relied on analyses presented only in supplementary figures. To address this, we have revised both the analysis and the corresponding text. Specifically, we now include a direct comparison of transient (injury-associated) and persistent (age- and irradiation-associated) SenFlag-positive cells in the revised manuscript (Fig. S4B). This analysis shows that persistent senescent cells exhibit a more pronounced inflammatory transcriptional profile, with increased expression of genes such as *Il6*, *Il7*, *Cx3cl1*, *Cxcl12*, and *Icam1*, compared to transient senescent cells. In contrast, transient SenFlag-positive cells display a profile consistent with a more regulated and potentially clearance-prone state. We have revised the text accordingly to ensure that all statements regarding inflammatory and apoptotic features are directly supported by data presented in the main or supplementary figures, and we have moderated the wording to avoid overinterpretation.

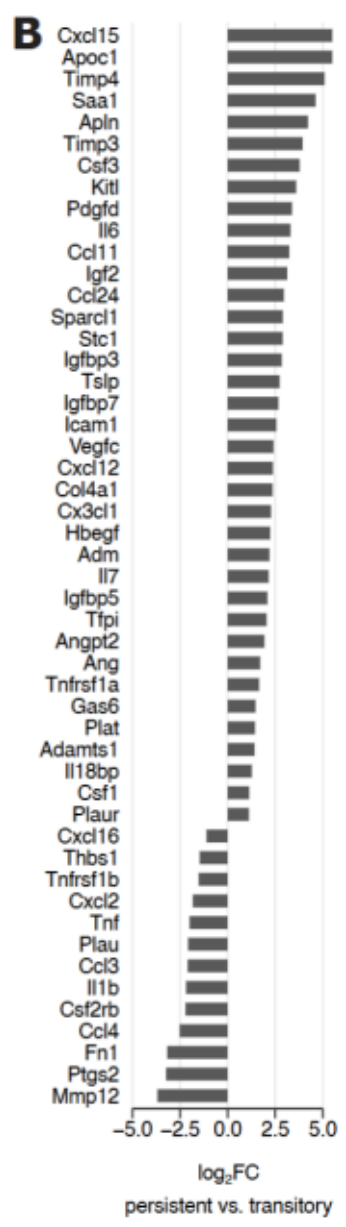

Revised Manuscript Fig. S4B

Reviewer #2:

This manuscript proposes "SenFlag," a transcriptional gene set intended to identify senescent cells in scRNA-seq datasets based primarily on coordinated cell-cycle arrest features, HMG loss, CCND1 upregulation, and lysosomal gene induction. The effort to define a mechanistically grounded and compact signature is commendable. However, several conceptual and validation issues limit the current strength of the conclusions.

Below I outline my main concerns.

Major Concerns

1. Lack of Benchmarking Against Established Senescence Signatures

A central limitation of the manuscript is the absence of systematic benchmarking of SenFlag against previously established senescence gene sets, particularly SASP-focused signatures such as SenMayo (Saul et al., Nat Commun 2022) and related frameworks.

Specifically, how does SenFlag perform relative to SenMayo in the same datasets? What is the overlap between SenFlag-positive and SenMayo-positive cells? Does SenFlag capture a distinct senescent subpopulation or largely overlap with existing classifiers? What is gained (specificity? mechanistic grounding? lower false positives?) and what is lost (e.g., inflammatory components)?

A direct, side-by-side comparison in the same in vivo scRNA-seq datasets would substantially strengthen the manuscript. Without such benchmarking, it is difficult to assess whether SenFlag represents an advance over existing approaches or simply a differently parameterized classifier.

AUTHORS: We thank the reviewer for this important suggestion and fully agree that benchmarking against established senescence signatures is essential. To address this, we performed a systematic comparison between SenFlag and previously published senescence gene sets, including the SASP-focused SenMayo signature (Saul et al., Nat Commun 2022), across the same in vivo scRNA-seq datasets. These analyses are now presented in Revised Manuscript Fig. 6. We find that SenFlag and SenMayo partially overlap but capture distinct cellular populations. Specifically, SenMayo identifies a broader set of cells enriched for inflammatory and SASP-associated transcriptional programs, including populations that are highly responsive to acute inflammatory stimuli (e.g., LPS-treated conditions). In contrast, SenFlag defines a more restricted subset characterized by coordinated cell-cycle arrest (low proliferation markers with *CDKN1A/CDKN2A* expression), HMG downregulation, and *CCND1* induction. Importantly, SenFlag reduces apparent false-positive classification in highly inflammatory but proliferative or transiently activated cell populations, which are frequently detected by SASP-based signatures. Conversely, SenFlag does not capture all SASP-positive cells, particularly those lacking clear evidence of stable cell-cycle arrest, reflecting a trade-off between specificity for senescence-associated cell-cycle arrest and sensitivity to inflammatory components. Thus, rather than replacing existing signatures, SenFlag complements them by providing a mechanistically grounded signature centered on stable cell-cycle arrest, enabling discrimination between senescence-associated and purely inflammatory transcriptional states. We have revised the manuscript to include this analysis

and to explicitly discuss the relationship between SenFlag and existing senescence signatures.

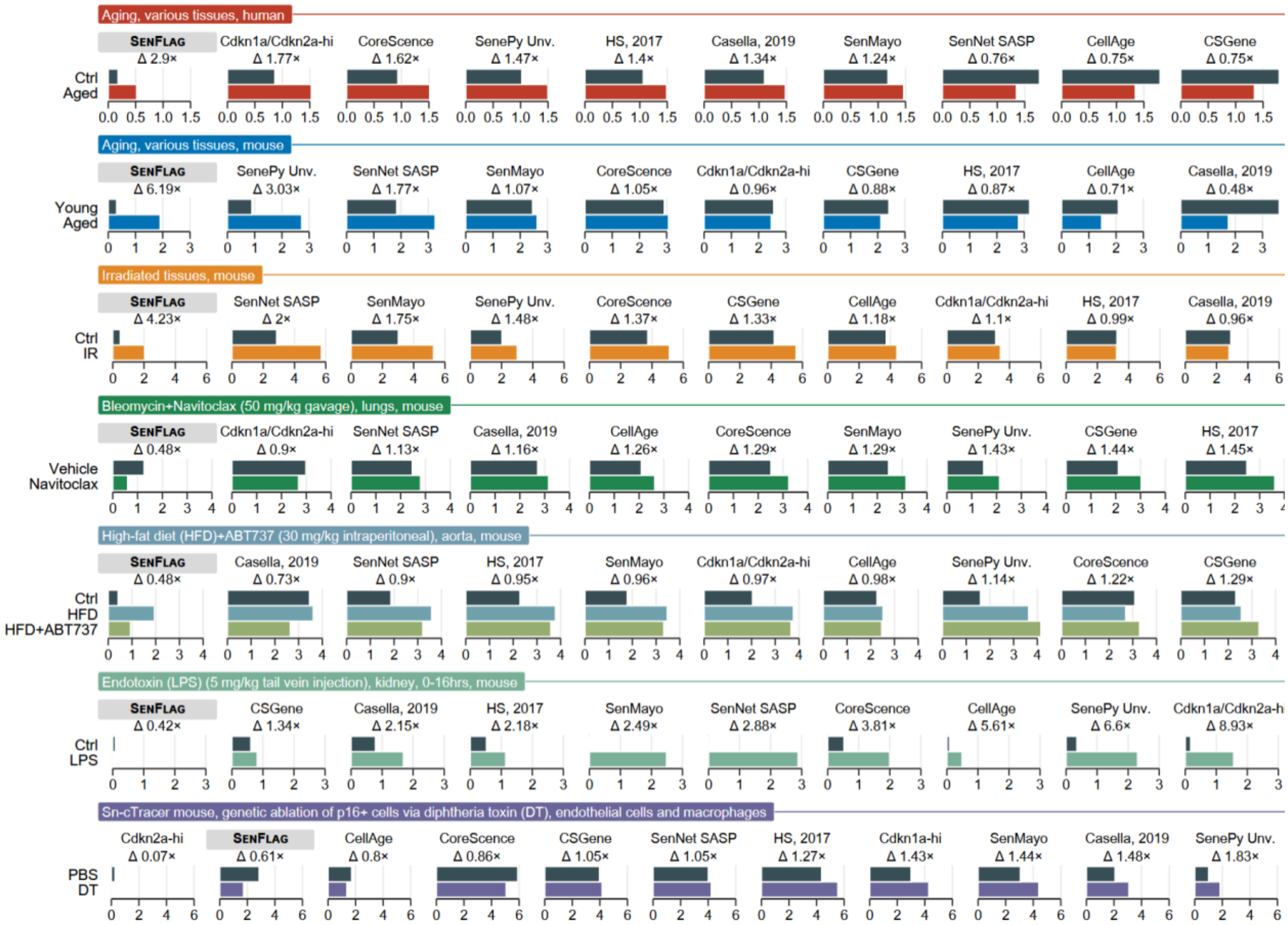

Revised Manuscript Fig. 6

2. In Vivo Sensitivity and Specificity Remain Insufficiently Demonstrated

SenFlag is derived largely from in vitro datasets and then applied to in vivo aging and injury models. While increased SenFlag-positive cells are observed with age and after injury, this does not sufficiently establish specificity for bona fide senescence versus broader aging-associated transcriptional changes.

Validation in genetic or pharmacological senescent cell clearance models would greatly strengthen the manuscript. Demonstrating that SenFlag detects both accumulation and reduction of senescent cells would provide critical support for its specificity.

AUTHORS: We thank the reviewer for this important point and agree that demonstrating sensitivity to senescent cell clearance is critical to support the specificity of SenFlag. To address this, we systematically identified and analyzed publicly available scRNA-seq datasets involving pharmacological or genetic senescent cell clearance. These analyses are now presented in Revised Manuscript Fig. 6 (shown above). Across these datasets, SenFlag-positive cells consistently show the expected directional behavior: they accumulate in conditions associated with increased senescence (e.g., aging or damage) and are reduced following senolytic interventions. This behavior is consistent across multiple models

and supports the ability of SenFlag to track both the presence and clearance of senescent cells *in vivo*. Importantly, when compared to alternative approaches (including SASP-based signatures and CDKN1A/CDKN2A-high selection), SenFlag shows reduced sensitivity to broadly inflammatory or transiently activated cell states, while maintaining responsiveness to senolytic treatment. This suggests that SenFlag preferentially captures a population consistent with bona fide senescent cells rather than general stress or inflammatory responses. We have revised the manuscript to include these analyses and to more clearly state the limitations and strengths of SenFlag in the context of in vivo specificity.

3. Regenerative Senescence: Lack of Temporal Resolution

The authors report that regenerative injury models show increased SenFlag-positive cells (~6-fold). However, in classical models (e.g., wound healing), senescence is considered transient. If SenFlag detects functional senescence, one would expect dynamic changes over time. Therefore, I am puzzled why is there no clear difference in senescence load between early and late stages of wound healing (Figure 4C)?

AUTHORS: We agree that temporal resolution is essential when interpreting regenerative senescence. We also acknowledge that the original figure combined datasets from independent studies, which limited the ability to draw conclusions about temporal dynamics. In particular, differences in sampling strategies (e.g., wound center versus edge) and cell composition across datasets can affect percentage-based estimates, especially for low-frequency populations such as SenFlag-positive cells. To address this concern, we have revised the analysis by focusing exclusively on datasets with multiple timepoints within the same experimental system. These results are now presented in Revised Manuscript Fig. 4C. Importantly, this revised analysis demonstrates clear temporal dynamics, with SenFlag-positive cells increasing during early injury phases and declining at later stages, consistent with the transient nature of regenerative senescence. We have removed the previously included datasets that could not support this type of analysis to avoid potential misinterpretation.

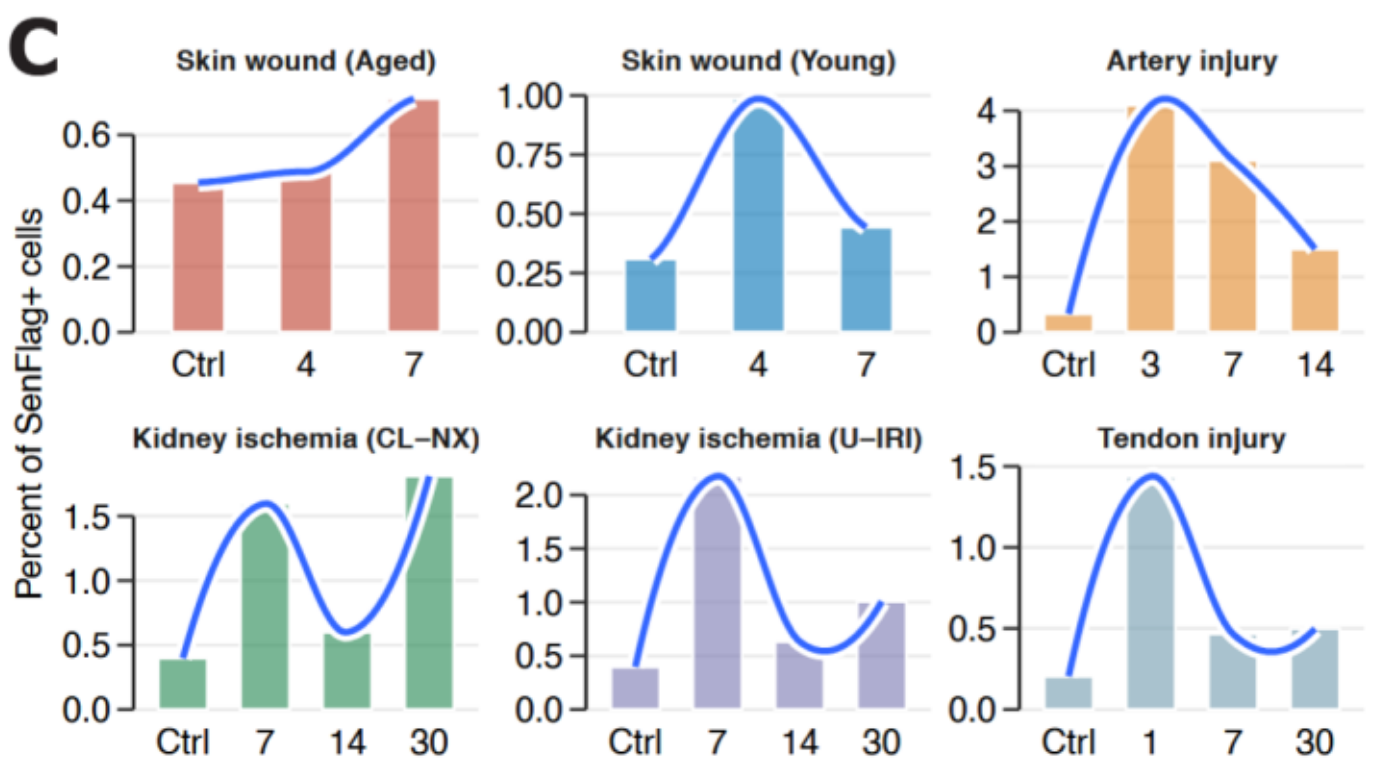

Revised Manuscript Fig. 4C

4. CCND1 Upregulation and Nuclear Localization of p16/p21

The reported correlation between CCND1 upregulation and nuclear localization of p16/p21 is conceptually interesting and potentially important. However, nuclear localization cannot be assessed in scRNA-seq datasets. It therefore remains unclear how this mechanistic insight can be operationalized in purely transcriptomic analyses.

If CCND1 is intended to function as a surrogate marker for nuclear CDKi-mediated cell cycle arrest, the authors should clarify what additional information is provided by retaining CDKN1A and CDKN2A within the SenFlag signature. Would a combination of CCND1, low proliferation markers, and HMG loss be sufficient to define the classifier?

Conversely, if CDKN1A and CDKN2A are required components, the manuscript should more clearly articulate their non-redundant contribution relative to CCND1. This point is particularly important in light of recent work demonstrating substantial heterogeneity and limited overlap between $p21^{+}$ and $p16^{+}$ senescent populations in vivo (PMID: 41162753). Given this reported divergence, it is essential to clarify how SenFlag reconciles or integrates distinct CDKi-positive senescent states.

AUTHORS: We agree that nuclear localization of p16/p21 cannot be directly assessed in scRNA-seq data. Our intent is not to infer localization per se, but to use *CCND1* upregulation as a functional transcriptional correlate of a state in which cell-cycle progression signals are present but effectively inhibited. Our *in vitro* experiments demonstrate that *CCND1* induction occurs when p16/p21 are localized to the nucleus (WT and NLS constructs), but not when they are restricted to the cytoplasm, supporting its use as a proxy for functionally active cell-cycle inhibition. Importantly, *CCND1* alone is not sufficient to define senescence, as it is also expressed in proliferating cells (Revised Manuscript Fig. 2H). Therefore, its inclusion in SenFlag serves to capture a specific context, namely cell-cycle arrest, rather than acting as a standalone marker. In contrast, *CDKN1A* and *CDKN2A* provide essential information on the activation of canonical cell-cycle inhibitory pathways. Their inclusion ensures that the classifier captures bona fide cell-cycle arrest and reduces false-positive identification of proliferating or transiently activated cells. Indeed, removing *CDKN1A/CDKN2A* from the SenFlag criteria increases apparent senescent cell detection in young tissues (Reviewer Figure 1), indicating reduced specificity.

Thus, *CCND1* and *CDKN1A/CDKN2A* contribute non-redundant information: *CCND1* reflects the presence of pro-proliferative signaling under arrest conditions, whereas *CDKN1A/CDKN2A* indicate activation of inhibitory pathways. Together, they enable inference of a functionally arrested state that cannot be captured by either component alone.

Regarding the reported heterogeneity between $p21^{+}$ and $p16^{+}$ populations (PMID: 41162753), SenFlag does not require co-expression of *CDKN1A* and *CDKN2A* within the same cell. Instead, it accommodates this heterogeneity by requiring expression of either marker, while integrating additional features (low proliferation, HMG downregulation, and *CCND1* induction) to identify a shared core senescence state across distinct CDKi-driven contexts. We have revised the manuscript to clarify these conceptual distinctions and the non-redundant contributions of each component of the SenFlag signature.

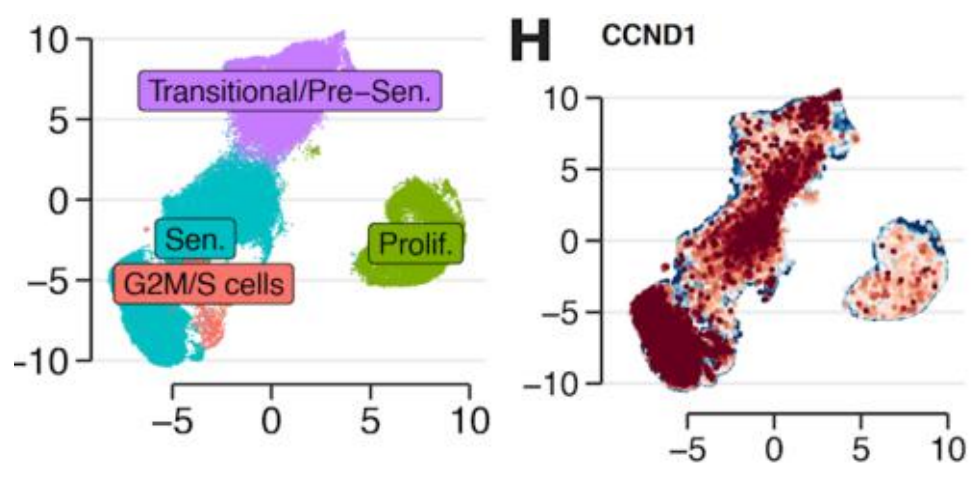

Revised Manuscript Fig. 2H

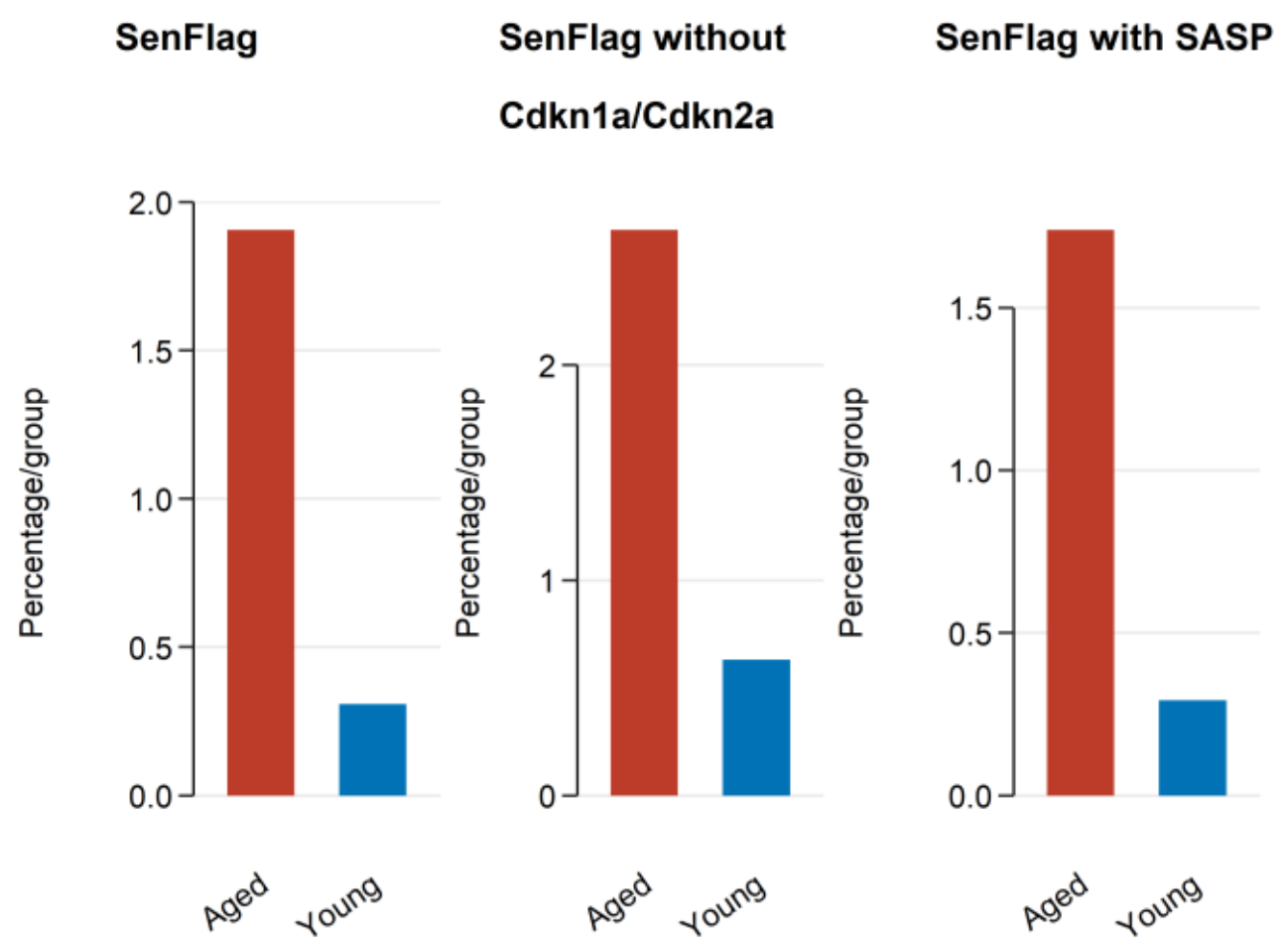

Reviewer Figure 1: Percentages of SenFlag+ cells in Tabula Muris atlas (Murine natural aging) with and without Cdkn1a/Cdkn2a, as well as minimal SASP panel. SASP was defined as at least one transcript of either of the following genes: Gdf15, Mmp1, Stc1, Timp1, Timp2, Mmp12, Mmp3, Il6, Il11, Il33, Ccl2, Ccl5, Cxcl1, Cxcl2, Cxcl3, Cxcr2, Cxcl5, Cxcl10, Cxcl14, Icam1, Col11a1, Col12a1, Thbs4, Mmp7

5. Limited Inclusion of SASP Components

This is going back to my first comment about benchmarking, given that inflammatory remodeling is a hallmark of senescence, it is surprising that SenFlag includes very few canonical SASP factors. The rationale for excluding many SASP genes requires clearer justification. While the authors argue for a mechanistic focus on cell-cycle arrest, inflammatory signaling is a defining biological and pathological feature of senescence in aging tissues. Beyond comparing side by side with SenMayo or other signature focusing on SASP factors, I am also wondering would combining SenFlag with a SASP-focused signature (e.g., SenMayo) improve specificity and biological interpretability?

AUTHORS: We thank the reviewer for this important point and fully agree that inflammatory remodeling and the SASP are central features of senescence biology. Our decision not to include SASP components in the core SenFlag signature reflects a design choice aimed at maximizing robustness across heterogeneous single-cell datasets. In our pooled bulk RNA-seq analyses, SASP-associated genes showed substantial variability depending on the senescence inducer, cell type, and context, and did not consistently distinguish senescent from non-senescent cells across conditions. In contrast, features related to stable cell-cycle arrest, chromatin-associated HMG loss, and lysosomal activity were consistently observed and therefore form the basis of SenFlag. Importantly, although SASP genes are not used as selection criteria, SenFlag-positive cells consistently display enrichment for inflammatory and SASP-associated transcriptional programs across datasets (Revised Manuscript Fig. 3M), indicating that SASP-related features are captured as downstream consequences of the SenFlag-defined state rather than as primary defining features. We agree with the reviewer that combining complementary approaches may enhance biological interpretability. To explore this, we performed a preliminary analysis integrating SenFlag with SASP-focused gene sets (Reviewer Figure 1, shown above). This did not substantially improve discrimination between aged and young tissues, suggesting that inclusion of SASP components does not necessarily enhance specificity in this context, likely due to the presence of SASP-like inflammatory programs in non-senescent cell populations. Thus, we view SenFlag as a minimal, mechanistically grounded signature that can be complemented by SASP-based signatures depending on the biological question. We have revised the manuscript to clarify this conceptual distinction and to explicitly discuss how SenFlag relates to SASP-focused approaches.

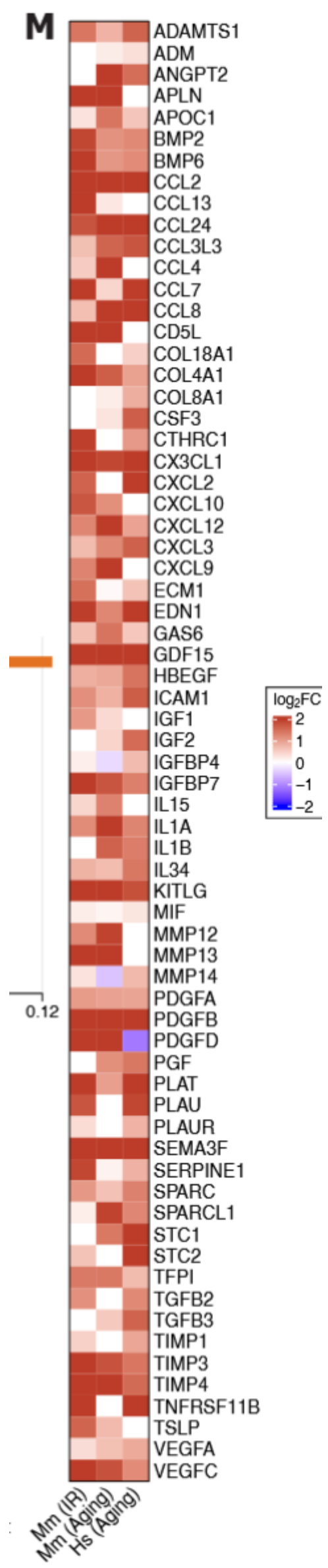

Revised Manuscript Fig. 3M

6. The manuscript presents seemingly conflicting interpretations regarding macrophages, at times suggesting they fail SenFlag criteria (Line 160-163) and at other times describing them as a major senescent population in injury (Line 189-190, Figure 4D). This point requires clarification.

AUTHORS: We agree that the original text could be interpreted as contradictory, and we have revised the manuscript to clarify this point. SenFlag does not uniformly classify all macrophages as senescent; rather, it distinguishes between different macrophage states. Specifically, activated immune cells (e.g., inflammatory or monocyte-derived populations) often express *Cdkn1a* but do not meet the full SenFlag criteria and are therefore excluded. In contrast, a subset of macrophages—most consistent with tissue-resident macrophages—fulfills all SenFlag conditions and is classified as SenFlag-positive. These SenFlag-positive macrophages exhibit transcriptional features consistent with senescence, including reduced proliferation, downregulation of HMG-associated genes, and co-expression of *CCND1* with

CDKN1A or *CDKN2A*. Importantly, this population is not restricted to injury conditions but is observed across multiple datasets, including aging and steady-state tissues. We have revised the relevant sections (Results Section 5 and Fig. 4) to explicitly distinguish between these macrophage subpopulations and to ensure consistent interpretation throughout the manuscript.

7. The manuscript would benefit from more comprehensive citation of prior work. For example, it is well reported the complex or paradoxical role of CCND1 in senescence (PMID: PMID: 8569279, https://www.biorxiv.org/content/10.1101/2025.08.01.668243v1). Besides, previous efforts to derive transcriptomic senescence signatures should be systemically discussed.

AUTHORS: We agree that a more comprehensive integration of prior work strengthens the manuscript. We have expanded the discussion of CCND1 to include key studies describing its complex and context-dependent role in senescence. These additions clarify that CCND1 upregulation in senescence has been previously observed and is consistent with its proposed role as a marker of cell-cycle signaling under arrest conditions. In addition, we have substantially expanded the discussion of previously proposed transcriptomic senescence signatures. In the revised manuscript (Results Section 6 and Discussion), we now provide a more systematic overview of existing approaches, including SASP-focused and cell-cycle–based signatures, and explicitly position SenFlag in relation to these frameworks. These additions aim to better contextualize our findings within the broader literature and clarify the conceptual advances provided by the SenFlag approach.

Reviewer #3:

Summary

Altulea and colleagues present SenFlag, a novel gene set designed to identify senescent cells based on downregulation of proliferation, chromatin architecture and quiescence genes and upregulation of cell-cycle inhibitors, CCND1 and lysosomal genes. Using this signature, they show how senescent cells accumulate in vivo in both humans and mice and identify the main cell types involved, notably endothelial and epithelial cells. This represents an important advance, as it at least partially addresses a major limitation of existing senescence gene sets, which often rely heavily on pro-inflammatory genes that can also be expressed in non-senescent contexts and vary depending on the inducer or the tissue involved.

1- Authors should discuss the success of eliminating CDKN2A+ cells in the light of their finding that this gene was rarely expressed in the population of senescent cells identified by SenFlag.

AUTHORS: We thank the reviewer for the general positive feedback on our work this insightful comment. We agree that the apparent discrepancy between the widespread use of CDKN2A-targeted senescent cell elimination strategies and the relatively low detection of *CDKN2A* transcripts in scRNA-seq datasets requires clarification. Importantly, the low frequency of *CDKN2A*-positive cells observed in our analyses primarily reflects technical limitations of single-cell RNA-seq, particularly sequencing depth and the use of snRNA-seq platforms, which are known to under-detect low-abundance transcripts such as *CDKN2A*. This does not imply that p16 is biologically absent or unimportant in senescent cells. Consistent with its established role, we find that *CDKN2A* exhibits higher specificity to aging even in the absence of additional criteria (Revised Manuscript Fig. S3A, C; Reviewer Fig. 2). Furthermore, analyses of p16-targeted senescent cell ablation (Revised Manuscript Fig. 6) show that SenFlag-positive cells are reduced following CDKN2A-based interventions, supporting the ability of SenFlag to capture p16-dependent senescent populations despite incomplete transcript detection. We have revised the manuscript to clarify this distinction between biological relevance and technical detectability, and to better contextualize SenFlag in relation to CDKN2A-based senescence models.

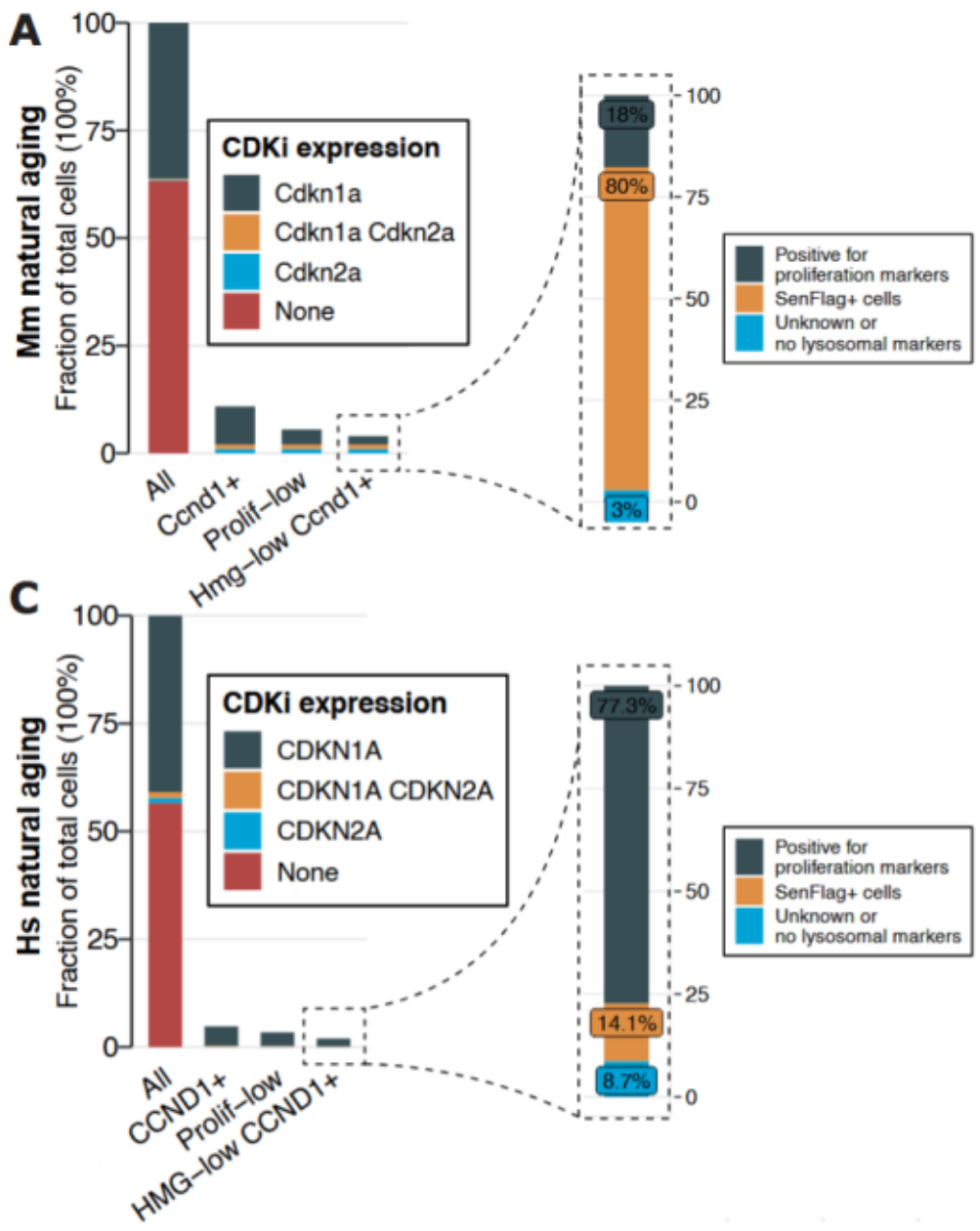

Revised Manuscript Fig. S3A, C

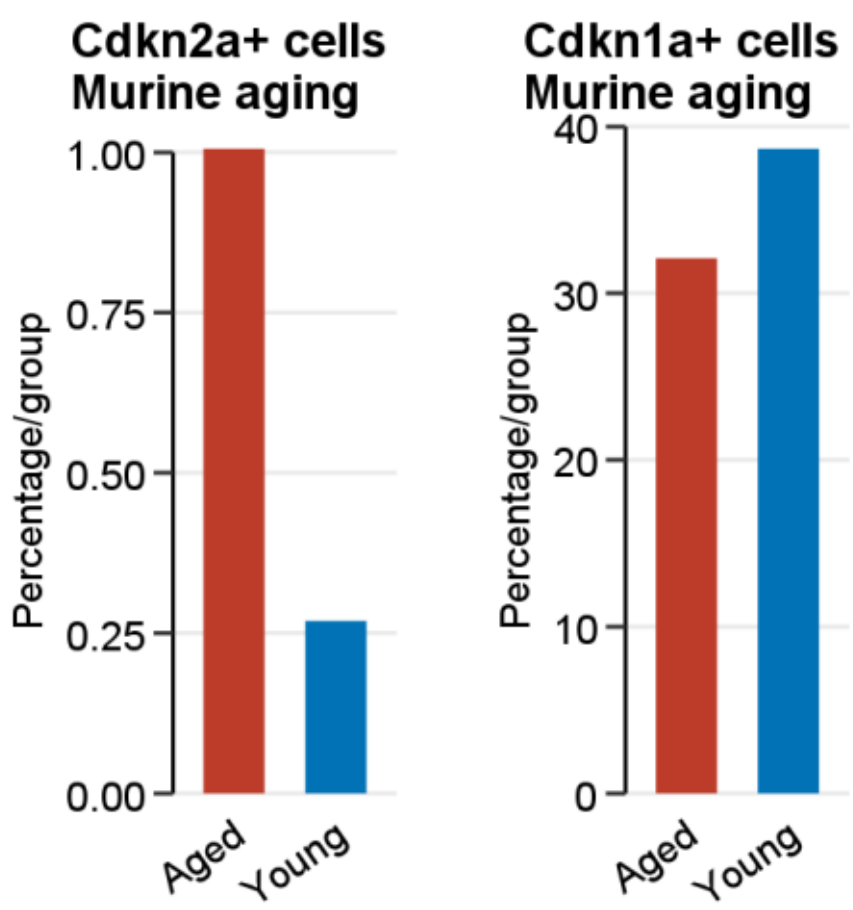

Reviewer Fig. 2

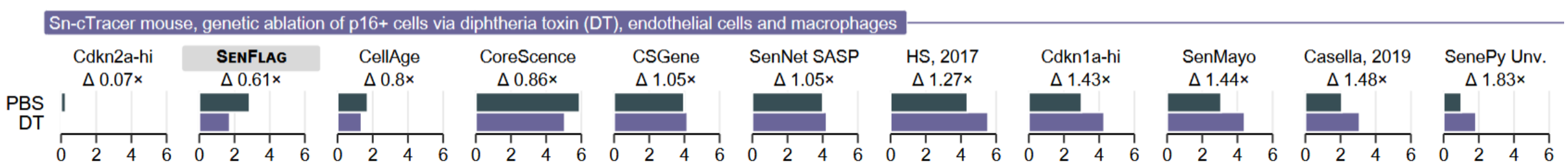

Revised Manuscript Fig. 6

2- Lines 211-214 state that injury-associated senescence is a transient state. However, the manuscript does not provide clear data to support this claim. While the reported correlation with increased expression of apoptotic genes may suggest that these cells eventually die, this is not directly demonstrated. Senescent cells could also be cleared by the immune system, but the senescent state itself might still be long-lasting; the current data do not distinguish between these possibilities.

AUTHORS: We thank the reviewer for this important point and agree that our original wording overstated the conclusions that can be drawn from the data. We acknowledge that single-cell transcriptomic analyses cannot directly determine the fate or duration of individual senescent cells, and therefore cannot distinguish between cell-intrinsic resolution (such as apoptosis) and extrinsic clearance (primarily immune-mediated removal). We have revised the manuscript to reflect this limitation more explicitly.

Our analyses support the presence of temporal dynamics in SenFlag-positive cell abundance across injury models, rather than directly demonstrating that senescent cells are intrinsically transient. In revised Fig. 4C, datasets with multiple timepoints within the same experimental system show an increase in SenFlag-positive cells during early injury phases followed by a decline at later stages, consistent with the resolution of injury-associated responses. While expression of apoptosis-related genes in these populations may suggest increased susceptibility to clearance, we agree that this does not constitute direct evidence of cell death. Accordingly, we have moderated our interpretation and now describe injury-associated senescence as a temporally regulated state, rather than definitively transient at the single-cell level. We have revised the relevant sections of the manuscript to clarify these distinctions and to avoid overinterpretation.

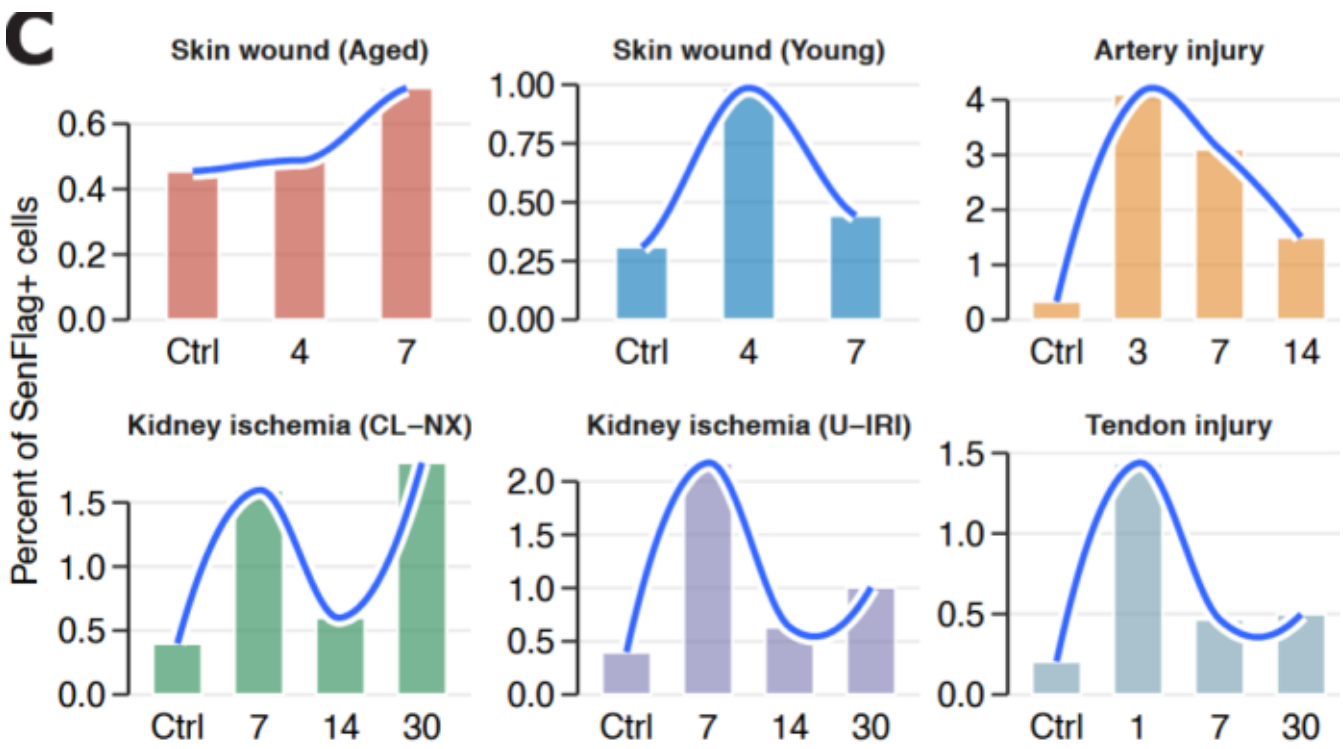

Revised Fig. 4C

3- In the discussion, lanes 224-231, the authors should acknowledge early work linking Cyclin D1 to senescence (1, 2).

AUTHORS: We have revised the Discussion to incorporate and explicitly discuss early studies linking Cyclin D1 (*CCND1*) to senescence. These references have been integrated into the relevant section to provide appropriate historical context and to emphasize that the association between *CCND1* upregulation and senescence has been previously observed. We have also clarified how our findings extend this earlier work by positioning *CCND1* within a combinatorial signature, where it serves as a context-dependent marker of cell-cycle signaling under arrest conditions, rather than as a standalone determinant of senescence.

4- Although the authors identify a clear link to chromatin architecture through downregulation of HMGB1 and HMGN2, it is possible that SenFlag may miss senescent cells in which chromatin is dysregulated through other mechanisms, see, for example, (3) for a recent

review. In addition, SenFlag does not explicitly capture transcriptomic consequences of epigenetic dysregulation such as derepression of repetitive elements, endogenous retroviruses, or increased lineage promiscuity.

AUTHORS: We thank the reviewer for this insightful comment and agree that senescence-associated chromatin dysregulation can arise through multiple mechanisms beyond HMG protein regulation. Our use of *HMGB1/2* and *HMGN2* downregulation reflects a consistent and early transcriptional feature observed across the senescence models analyzed (Revised Manuscript Fig. 1G), rather than a comprehensive representation of all chromatin alterations associated with senescence. We agree that alternative mechanisms, including changes in histone modifications, DNA methylation, nuclear architecture, and derepression of repetitive elements, may also contribute to chromatin remodeling in a context-dependent manner.

With respect to transcriptomic consequences such as transposable element activation, endogenous retrovirus expression, and lineage plasticity, we agree that these phenomena have been reported in specific contexts. However, they are not consistently observed across senescence models and are difficult to robustly quantify in standard scRNA-seq data due to technical limitations, including multi-mapping of repetitive sequences and gene-centric annotation pipelines. To address this point, we performed additional analyses of pathway-level signatures related to chromatin organization and dsRNA/interferon responses (Reviewer Fig. 3). These analyses show trends consistent with chromatin remodeling, although with variability across contexts. We have incorporated these observations into the revised manuscript and clarified that such features likely represent context-dependent downstream manifestations rather than core, universally detectable components of senescence. Accordingly, we have revised the Discussion to explicitly acknowledge that SenFlag captures a minimal and conserved transcriptional framework, and may not detect all senescent states associated with alternative chromatin or epigenetic alterations.

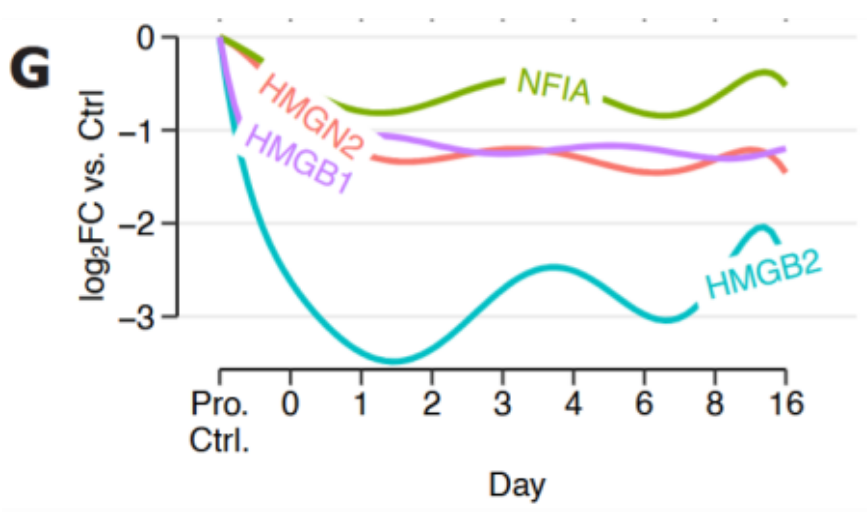

Revised Manuscript Fig. 1G

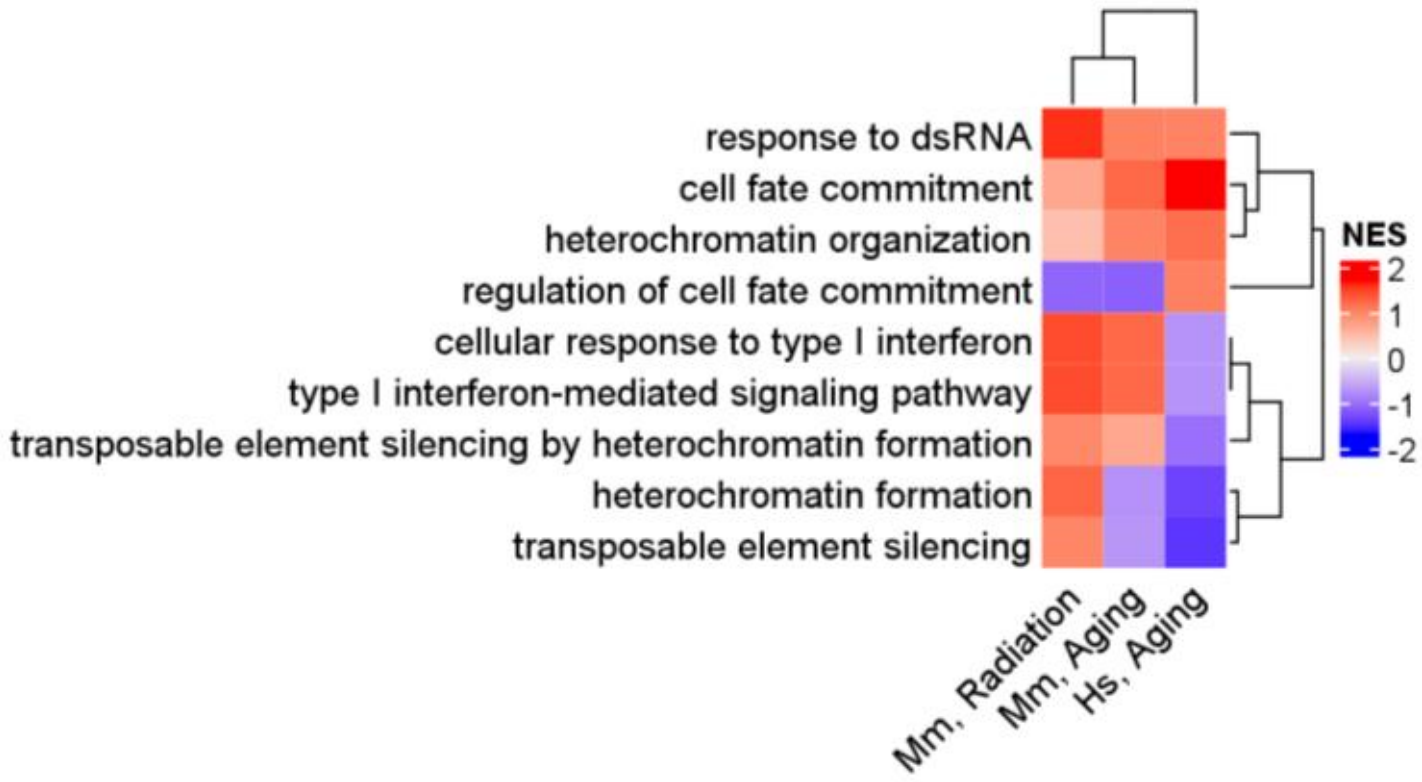

Reviewer Fig. 3: Heatmap showing GO terms analysis of SenFlag+ cells in the indicated conditions vs. controls

Referee #3, additional cross-comments:

I also agree with my colleagues' comments. My enthusiasm for this paper stems from the fact that it proposes an alternative gene set which, given the substantial heterogeneity we are uncovering in senescence programs, may prove useful in certain contexts. SenFlag appears to emphasize cell-cycle arrest and chromatin, whereas most other signatures are more heavily driven by SASP-related genes.

That said, the authors will need to address all comments convincingly to support the validity and appropriate use of their signature. For me, the most important point is to obtain practical validation in relevant in vivo models-ideally demonstrating that interventions known to improve outcomes by clearing senescent cells also reduce the burden of SenFlag-positive cells.

AUTHORS: We thank the reviewer for this encouraging assessment and fully agree that in vivo validation is essential to support the practical utility of SenFlag. To address this key point, we performed a systematic analysis of publicly available scRNA-seq datasets involving senescence-modulating interventions, including pharmacological and genetic approaches associated with senescent cell clearance. These results are now presented in Revised Manuscript Fig. 6. Across these models, SenFlag-positive cells show the expected directional behavior: they accumulate in conditions associated with increased senescence and are reduced following interventions known to improve outcomes through senescent cell

clearance. This provides functional support for the specificity of SenFlag in identifying senescent cell populations in vivo. In addition, comparison with alternative approaches (including SASP-based signatures and CDKN1A/CDKN2A-high selection) indicates that SenFlag is less sensitive to broadly inflammatory or transiently activated states, while remaining responsive to senolytic interventions. We have revised the manuscript to highlight these validation analyses and to clarify the contexts in which SenFlag is most appropriately applied, particularly as a complementary framework to existing signatures.

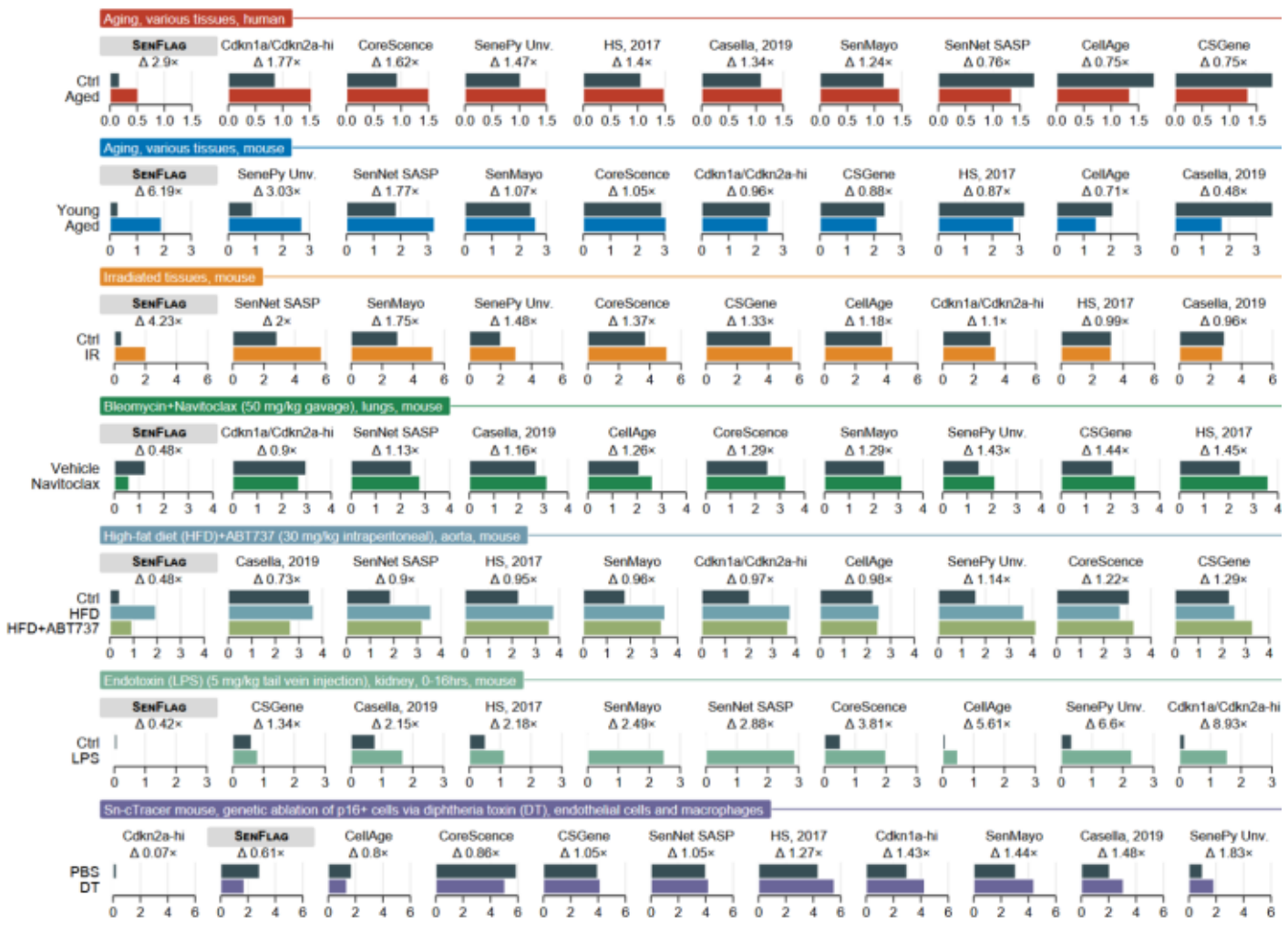

Revised Manuscript Fig. 6

Dear Dr Demaria,

Thank you for your rebuttal and submitting your amended manuscript (EMBOJ-2026-123584R-Q) to The EMBO Journal, together with a point-by-point response to the issues raised during peer-review. As mentioned, based on your revisions, we decided to send the complemented study back to the referees for their scientific reassessment, and we have received detailed re-reports from all of them, which I enclose below. As you will see, the reviewers state that the work has been substantially enhanced by the revision and they are now in favour of publication.

Thus, we are pleased to inform you that your manuscript has been accepted in principle for publication in The EMBO Journal.

We now need you to take care of a number of minor issues related to formatting and data annotation, as detailed below.

Please submit a revised version of the manuscript using the link enclosed below.

As you might remember from previous experience, every paper at the EMBO Journal includes a 'Synopsis', displayed on the html and freely accessible to all readers. The synopsis includes a 'model' figure as well as 2-5 one-short-sentence bullet points that summarize the article. I would appreciate if you could provide this figure and the bullet points.

Thank you again for giving us the chance to consider your manuscript for The EMBO Journal, I look forward to hearing from you and receiving your final revised version of the manuscript.

Best regards,

Daniel Klimmeck

Daniel Klimmeck PhD
Senior Editor
The EMBO Journal

Formatting changes required for the revised version of the manuscript:

>> Author information: author contributions should be marked in our online system; corresponding author needs to be labelled in the manuscript and corresponding author's email added on the title page.

>> Author Contributions: Remove the author contributions information from the manuscript text. Note that CRediT has replaced the traditional author contributions section as of now because it offers a systematic machine-readable author contributions format that allows for more effective research assessment. and use the free text boxes beneath each contributing author's name to add specific details on the author's contribution.

More information is available in our guide to authors.
https://www.embopress.org/page/journal/14602075/authorguide

>> Provide a completed Author Checklist.

>> Limit the abstract to maximally 175 words.

>> Enter a 'Disclosure and Competing Interests Statement' section after the Acknowledgements.

>> Please correct the order and names of the manuscript sections as follows: Title page - Abstract - Introduction - Results - Discussion - Methods - Data Availability - Acknowledgements - Disclosure and Competing Interests Statement - References - Figure Legends - Table(s) - Expanded View Figure Legends.

>> Add a separate 'Statistical Analysis' section to the Methods part, detailing the algorithms and statistical tests applied.

>> References: please adjust reference format to EMBO Journal format, 10 authors et al. DOIs should only be used for preprints and datasets that have not been published yet

>> Please recheck references for the bioRxiv entries listed and update the citation if in the meantime published as regular article.

>> Funding: please enter funding information to the manuscript in the Acknowledgements section.

>> Figure callouts: all callouts should be listed sequentially.

>> Please provide source data for the study as to the earlier separate request e-mail by our office team. Source data should be uploaded as one (zipped) file per figure. Further, provide a high-resolution Appendix File.

>> Add a Reagents and Tools table to the Methods section, as a separate file using the existing template in the Guide For Authors, listing key reagents, experimental models, software and relevant equipment.

>> Consider additional changes and comments from our production team as indicated below:

- Figure Legends (main + EV):
1. Please note that the exact p values are not provided in the legends of 1I; 4H; 5A,C; S1-A

2. Please indicate the statistical test used for data analysis in the legends of figure 2M,Q; 4H; 5A,C,G; S1-A,D; S4-B.

3. Please note that the box plots need to be defined in terms of minima, maxima, centre, bounds of box and whiskers, and percentile in the legends of figures 5A,C; S1-C.

4. Please note that information related to n is missing in the legends of 5A,C; S1-C; S2-A.

5. Although 'n' is provided, please describe the nature of entity for 'n' in the legends of figures 1I,J.

6. Please note that the error bars are not defined in the legends of S1-D,H,I.

Further information is available in our Guide For Authors: https://link.springer.com/journal/44318/submission-guidelines

----

Referee #1:

I thank the authors for considering and addressing my comments. I found specially relevant the addition of Fig. 6. I commend the authors for this nice work.

Referee #2:

Thank you for sharing the rebuttal response; I only looked at the response to my comments and I agree that they are quite thorough in addressing my main concerns. In light of this, I recommend further consideration.

Referee #3:

In my first review, I was highly enthusiastic about this work because multiple senescence signatures are needed to help the field navigate the growing recognition that senescence is highly heterogeneous. SenFlag provides a SASP-independent signature that offers a valuable alternative, much as different algorithms have helped other fields better understand complex biological processes.

My major concern was the lack of validation in practical examples. The authors have now addressed this point convincingly and demonstrate the usefulness of the signature. SenFlag-positive cells show the expected directional behavior: they accumulate in conditions associated with increased senescence and are reduced following interventions known to improve outcomes through senescent cell clearance. This provides functional support for the specificity of SenFlag in identifying senescent cell populations in vivo.

I recommend this paper for publication in EMBO. I have also read the comments from the other reviewers, and I believe the authors have addressed them satisfactorily.

The authors addresses the remaining editorial issues.

Dear Dr Demaria,

Thank you for submitting the revised version of your Resource manuscript. I have now evaluated your amended manuscript and concluded that the remaining minor concerns have been sufficiently addressed.

I am thus pleased to inform you that your manuscript has been accepted for publication in the EMBO Journal.

Your manuscript will be processed for publication by EMBO Press. It will be copy edited and you will receive page proofs prior to publication. Please note that you will be contacted by Springer Nature Author Services to complete licensing and payment information.

Please note that it is The EMBO Journal policy for the transcript of the editorial process (containing referee reports and your response letter) to be published as an online supplement to each paper. If you do NOT want this, you will need to inform the Editorial Office via email immediately. More information is available here: https://www.embopress.org/transparent-process#Review_Process

You may qualify for financial assistance for your publication charges - either via a Springer Nature fully open access agreement or an EMBO initiative. Check your eligibility: https://www.embopress.org/page/journal/14602075/authorguide#chargesguide

Should you be planning a Press Release on your article, please notify the production team via this form in order to coordinate publication and release dates: https://forms.cloud.microsoft/e/eH4A8JVP6g

If you have any questions, please do not hesitate to contact the Editorial Office. Thank you for your contribution to The EMBO Journal.

Thank you again for this contribution to The EMBO Journal and congratulations on a successful publication! Please consider us again in the future for your most exciting work.

Best regards,

Daniel Klimmeck

Daniel Klimmeck, PhD
Senior Editor
The EMBO Journal
EMBO
Postfach 1022-40
Meyerhofstrasse 1
D-69117 Heidelberg
contact@embojournal.org

*****

Please note that it is The EMBO Journal policy for the transcript of the editorial process (containing referee reports and your

response letters) to be published as an online supplement to each paper. If you should prefer removal of any referee-only figures included in the point-by-point response(s), e.g. because they may still be used for future publication or because they have been reproduced from published work by others, please do let us know immediately via response email.
More information is available here: https://link.springer.com/partners/embo-press/editorial-policies#Peer%20review

** Click here to be directed to your login page: https://emboj.msubmit.net

EMBO Press Author Checklist

Corresponding Author Name: Marco Demaria	
Journal Submitted to: The EMBO Journal	
Manuscript Number: EMBOJ-2026-123584R1	

USEFUL LINKS FOR COMPLETING THIS FORM
The EMBO Journal - Author Guidelines
EMBO Reports - Author Guidelines
Molecular Systems Biology - Author Guidelines
EMBO Molecular Medicine - Author Guidelines

Reporting Checklist for Life Science Articles (updated January
This checklist is adapted from Materials Design Analysis Reporting (MDAR) Checklist for Authors. MDAR establishes a minimum set of requirements in transparent reporting in the life sciences (see Statement of Task: 10.31222/osf.io/9sm4x). Please follow the journal's guidelines in preparing your
Please note that a copy of this checklist will be published alongside your article.

Abridged guidelines for figures
1. Data
The data shown in figures should satisfy the following conditions:
- the data were obtained and processed according to the field's best practice and are presented to reflect the results of the experiments in an accurate and unbiased manner.
- ideally, figure panels should include only measurements that are directly comparable to each other and obtained with the same assay.
- plots include clearly labeled error bars for independent experiments and sample sizes. Unless justified, error bars should not be shown for technical
- if n<5, the individual data points from each experiment should be plotted. Any statistical test employed should be justified.
- Source Data should be included to report the data underlying figures according to the guidelines set out in the authorship guidelines on Data

2. Captions
Each figure caption should contain the following information, for each panel where they are relevant:
- a specification of the experimental system investigated (eg cell line, species name).
- the assay(s) and method(s) used to carry out the reported observations and measurements.
- an explicit mention of the biological and chemical entity(ies) that are being measured.
- an explicit mention of the biological and chemical entity(ies) that are altered/varied/perturbed in a controlled manner.
- the exact sample size (n) for each experimental group/condition, given as a number, not a range;
- a description of the sample collection allowing the reader to understand whether the samples represent technical or biological replicates (including how many animals, litters, cultures, etc.).
- a statement of how many times the experiment shown was independently replicated in the laboratory.
- definitions of statistical methods and measures:
  - common tests, such as t-test (please specify whether paired vs. unpaired), simple χ2 tests, Wilcoxon and Mann-Whitney tests, can be unambiguously identified by name only, but more complex techniques should be described in the methods section;
  - are tests one-sided or two-sided?
  - are there adjustments for multiple comparisons?
  - exact statistical test results, e.g., P values = x but not P values < x;
  - definition of 'center values' as median or average;
  - definition of error bars as s.d. or s.e.m.

Please complete ALL of the questions below.
Select "Not Applicable" only when the requested information is not relevant for your study.

Materials

Newly Created Materials	Information included in the manuscript?	In which section is the information available? (Reagents and Tools Table, Materials and Methods, Figures, Data Availability Section)
New materials and reagents need to be available; do any restrictions apply?	Not Applicable	

Antibodies	Information included in the manuscript?	In which section is the information available? (Reagents and Tools Table, Materials and Methods, Figures, Data Availability Section)
For **antibodies** provide the following information:<br>- Commercial antibodies: RRID (if possible) or supplier name, catalogue number and or/clone number<br>- Non-commercial: RRID or citation	Yes	Reagents and Tools Table

DNA and RNA sequences	Information included in the manuscript?	In which section is the information available? (Reagents and Tools Table, Materials and Methods, Figures, Data Availability Section)
**Short novel DNA or RNA including primers, probes**: provide the sequences.	Yes	Reagents and Tools Table; supplied Source Data file

Cell materials	Information included in the manuscript?	In which section is the information available? (Reagents and Tools Table, Materials and Methods, Figures, Data Availability Section)
**Cell lines:** Provide species information, strain. Provide accession number in repository **OR** supplier name, catalog number, clone number, and/**OR** RRID.	Yes	Reagents and Tools Table
**Primary cultures:** Provide species, strain, sex of origin, genetic modification status.	Not Applicable	
Report if the cell lines were recently **authenticated** (e.g., by STR profiling) and tested for mycoplasma contamination.	Yes	Materials and Methods

Experimental animals	Information included in the manuscript?	In which section is the information available? (Reagents and Tools Table, Materials and Methods, Figures, Data Availability Section)
**Laboratory animals or Model organisms:** Provide species, strain, sex, age, genetic modification status. Provide accession number in repository **OR** supplier name, catalog number, clone number, **OR** RRID.	Yes	Supplementary Methods section (mice were used for a supplementary experiment)
**Animal observed in or captured from the field:** Provide species, sex, and age where possible.	Not Applicable	
Please detail **housing and husbandry conditions**.	Yes	Supplementary Methods section

Plants and microbes	Information included in the manuscript?	In which section is the information available? (Reagents and Tools Table, Materials and Methods, Figures, Data Availability Section)
**Plants:** provide species and strain, ecotype and cultivar where relevant, unique accession number if available, and source (including location for collected wild specimens).	Not Applicable	
**Microbes:** provide species and strain, unique accession number if available, and source.	Not Applicable	

Human research participants	Information included in the manuscript?	In which section is the information available? (Reagents and Tools Table, Materials and Methods, Figures, Data Availability Section)
If collected and within the bounds of privacy constraints report on age, sex and gender or ethnicity for all study participants.	Not Applicable	

Core facilities	Information included in the manuscript?	In which section is the information available? (Reagents and Tools Table, Materials and Methods, Figures, Data Availability Section)
If your work benefited from core facilities, was their service mentioned in the acknowledgments section?	Not Applicable	

Design

Study protocol	Information included in the manuscript?	In which section is the information available? (Reagents and Tools Table, Materials and Methods, Figures, Data Availability Section)
If study protocol has been **pre-registered, provide DOI in the manuscript**. For clinical trials, provide the trial registration number **OR** cite DOI.	Not Applicable	
Report the **clinical trial registration number** (at ClinicalTrials.gov or equivalent), where applicable.	Not Applicable	

Laboratory protocol	Information included in the manuscript?	In which section is the information available? (Reagents and Tools Table, Materials and Methods, Figures, Data Availability Section)
Provide DOI OR other citation details if **external detailed step-by-step protocols** are available.	Not Applicable	

Experimental study design and statistics	Information included in the manuscript?	In which section is the information available? (Reagents and Tools Table, Materials and Methods, Figures, Data Availability Section)
Include a statement about **sample size** estimate even if no statistical methods were used.	Not Applicable	
Were any steps taken to minimize the effects of subjective bias when allocating animals/samples to treatment (e.g. **randomization procedure**)? If yes, have they been described?	Not Applicable	
Include a statement about **blinding** even if no blinding was done.	Not Applicable	
Describe **inclusion/exclusion criteria** if samples or animals were excluded from the analysis. Were the criteria pre-established?<br><br>If sample or data points were omitted from analysis, report if this was due to attrition or intentional exclusion and provide justification.	Not Applicable	
For every figure, are **statistical tests** justified as appropriate? Do the data meet the assumptions of the tests (e.g., normal distribution)? Describe any methods used to assess it. Is there an estimate of variation within each group of data? Is the variance similar between the groups that are being statistically compared?	Yes	Materials and Methods section. The analyses were done using standard and validated R packages

Sample definition and in-laboratory replication	Information included in the manuscript?	In which section is the information available? (Reagents and Tools Table, Materials and Methods, Figures, Data Availability Section)
In the figure legends: state number of times the experiment was **replicated** in laboratory.	Yes	Figure captions
In the figure legends: define whether data describe **technical or biological replicates**.	Yes	Figure captions

Ethics

Ethics	Information included in the manuscript?	In which section is the information available? (Reagents and Tools Table, Materials and Methods, Figures, Data Availability Section)
Studies involving **human participants**: State details of **authority granting ethics approval** (IRB or equivalent committee(s), provide reference number for approval.	Not Applicable	
Studies involving **human participants**: Include a statement confirming that **informed consent** was obtained from all subjects and that the experiments conformed to the principles set out in the WMA Declaration of Helsinki and the Department of Health and Human Services Belmont Report.	Not Applicable	
Studies involving **human participants:** For publication of **patient photos**, include a statement confirming that consent to publish was obtained.	Not Applicable	
Studies involving experimental **animals**: State details of **authority granting ethics approval** (IRB or equivalent committee(s), provide reference number for approval. Include a statement of compliance with ethical regulations.	Yes	Supplementary Methods section (mice were used for a supplementary experiment)
Studies involving **specimen and field samples:** State if relevant **permits** obtained, provide details of authority approving study; if none were required, explain why.	Not Applicable	

Dual Use Research of Concern (DURC)	Information included in the manuscript?	In which section is the information available? (Reagents and Tools Table, Materials and Methods, Figures, Data Availability Section)
Could your study fall under dual use research restrictions? Please check biosecurity documents and list of **select agents and toxins** (CDC): https://www.selectagents.gov/sat/list.htm	Not Applicable	
If you used a select agent, is the security level of the lab appropriate and reported in the manuscript?	Not Applicable	
If a study is subject to dual use research of concern regulations, is the name of the **authority granting approval and reference number** for the regulatory approval provided in the manuscript?	Not Applicable	

Reporting

The MDAR framework recommends adoption of discipline-specific guidelines, established and endorsed through community initiatives. Journals have their own policy about requiring specific guidelines and recommendations to complement MDAR.

Adherence to community standards	Information included in the manuscript?	In which section is the information available? (Reagents and Tools Table, Materials and Methods, Figures, Data Availability Section)
State if relevant guidelines or checklists (e.g., **ICMJE, MIBBI, ARRIVE, PRISMA**) have been followed or provided.	Not Applicable	
For **tumor marker prognostic studies,** we recommend that you follow the **REMARK** reporting guidelines (see link list at top right). See author guidelines, under 'Reporting Guidelines'. Please confirm you have followed these guidelines.	Not Applicable	
For **phase II and III randomized controlled trials**, please refer to the **CONSORT** flow diagram (see link list at top right) and submit the CONSORT checklist (see link list at top right) with your submission. See author guidelines, under 'Reporting Guidelines'. Please confirm you have submitted this list.	Not Applicable	

Data Availability

Data availability	Information included in the manuscript?	In which section is the information available? (Reagents and Tools Table, Materials and Methods, Figures, Data Availability Section)
Have **primary datasets** been deposited according to the journal's guidelines (see 'Data Deposition' section) and the respective accession numbers provided in the Data Availability Section?	Not Applicable	
Were **human clinical and genomic datasets** deposited in a public access-controlled repository in accordance to ethical obligations to the patients and to the applicable consent agreement?	Not Applicable	
Are **computational models** that are central and integral to a study available without restrictions in a machine-readable form? Were the relevant accession numbers or links provided?	Yes	Materials and Methods; supplied Source Data
If publicly available data were reused, provide the respective **data citations in the reference list**.	Yes	Materials and Methods
