# Supplementary material for: SenFlag gene signature identifies senescent cells in mouse and human tissues through a conserved core transcriptional program
Source: EMBO J. 2026 Jul 6;45(15):5580–606. doi: 10.1038/s44318-026-00845-6 (PMC13433995; doi:10.1038/s44318-026-00845-6)
Supplement: Supplementary file 2 [file 44318_2026_845_MOESM6_ESM.pdf]

Expanded View Figures

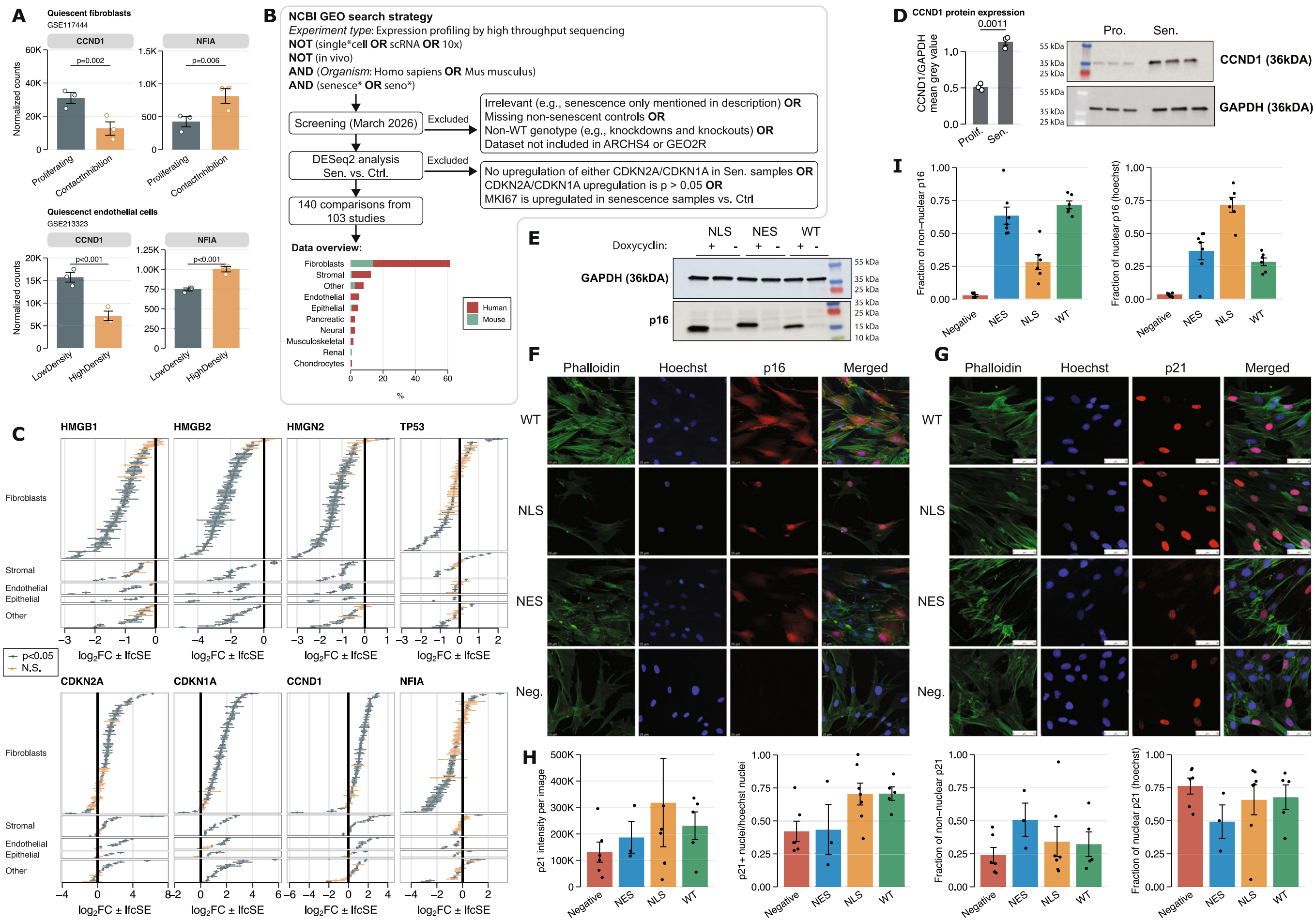

Figure EV1. Supporting analyses for identification of cell cycle-associated and quiescence-related transcriptional features.

(**A**) Normalized expression levels of *CCND1* and *NFIA* in quiescent endothelial cells and fibroblasts, induced by contact inhibition ($n = 3$ biological replicates per condition). For GSE213323, the adjusted p values are 2e-09 for *CCND1* and 0.00269 for *NFIA*. Statistical values were obtained using DESeq2. (**B**) Overview of the search strategy used to identify senescence-associated datasets from NCBI GEO for pooled analysis. (**C**) Log_2 fold change values (±lfcSE) from DESeq2 for indicated genes across datasets identified in (**B**), stratified by cell type. *TP53* results include the murine ortholog *Trp53*. $N = 139$–140 senescent vs. ctrl. comparisons from 103 unique studies. (**D**) Western blot analysis of *CCND1* expression in senescent IMR90 cells treated once with 250 µM doxorubicin and collected after 14 days. Quantification is shown relative to GAPDH (fluorescent) ($n = 3$ biological replicates per condition). Statistical comparisons were performed using a two-tailed t-test. Bars represent group means. Error bars indicate ±standard error of the mean. (**E**) Western blot analysis of p16 expression in BJ cells across indicated constructs and doxycycline conditions following 48 h of treatment (doxycycline or DMSO control). (**F**, **G**) Representative immunofluorescence images of BJ cells showing subcellular distribution of p16 (**F**) and p21 (**G**) under the indicated localization constructs. (**H**, **I**) Quantification of immunofluorescence images from panels (**F**) and (**G**), including signal intensity and colocalization with nuclear staining, for p21 (**H**) and p16 (**I**) ($n = 5$ images from independent samples). Bars represent group means. Error bars indicate ±standard error of the mean. Source data are available online for this figure.

 © The Author(s)

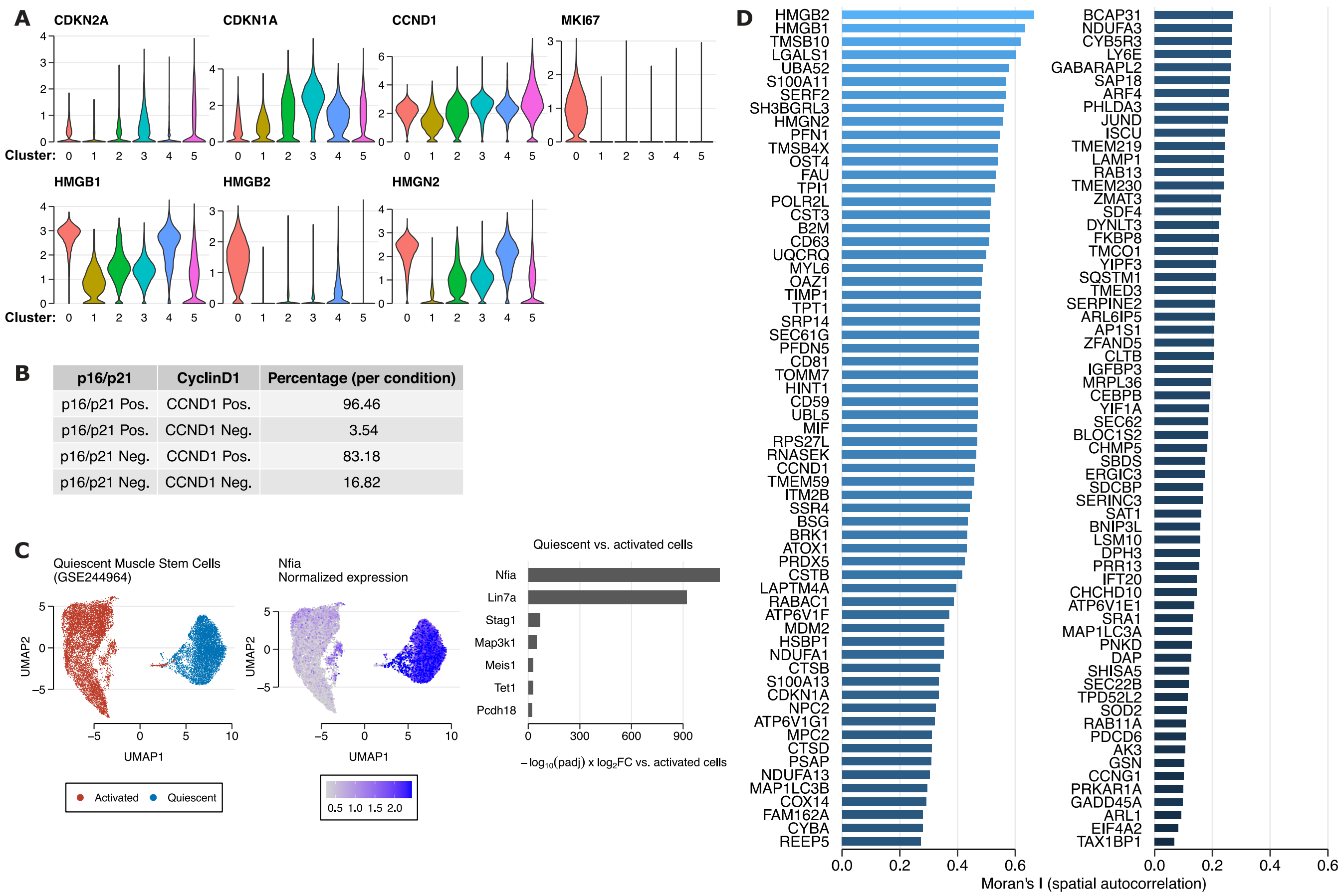

Figure EV2. Single-cell characterization of senescence- and quiescence-associated transcriptional features.

(**A**) Violin plots showing normalized expression of indicated genes across Louvain clusters from the integrated in vitro senescent cell dataset (Fig. 2C) ($n = 3$k–18k cells/cluster; 68k cells in total). (**B**) Numerical representation corresponding to the river plot shown in Fig. 2K. (**C**) Analysis of quiescent muscle stem cell data showing cell annotations, normalized *Nfia* expression, and candidate marker genes distinguishing quiescent from activated states. Gene selection was based on the results from Fig. 1C. (**D**) Top genes identified from pseudotime analysis based on spatial expression patterns, presented as Moran's I spatial correlation values.

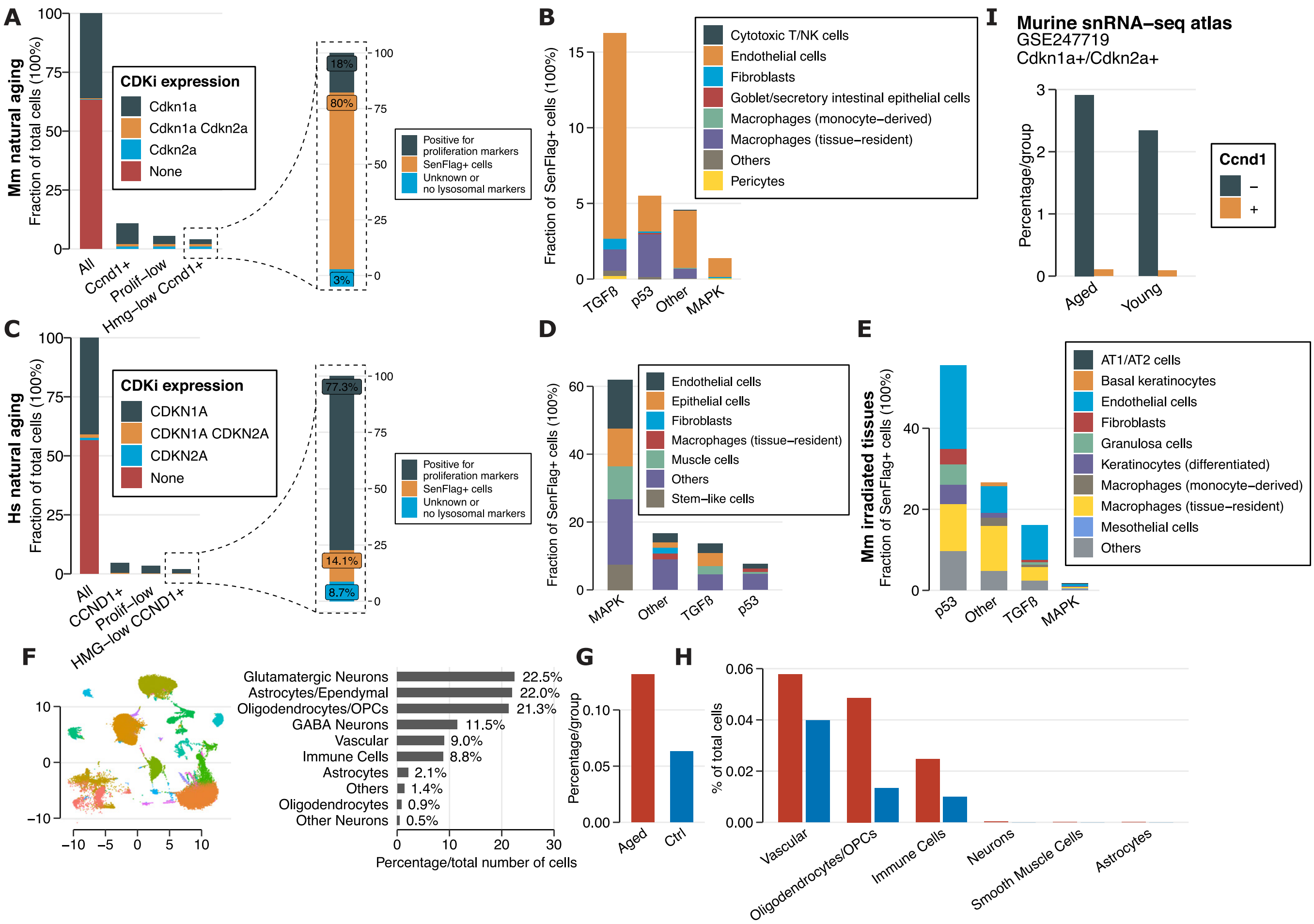

Figure EV3. Stepwise application of SenFlag and pathway characterization across in vivo datasets.

(**A**) Bar plot showing the cellular distribution of cells at successive steps of SenFlag application in the Tabula Muris dataset (murine aging). (**B**) Fraction of SenFlag+ cells expressing downstream targets of indicated pathways in the Tabula Muris dataset, stratified by cell type. (**C**) Bar plot showing the cellular distribution of cells at successive steps of SenFlag application in the Tabula Sapiens dataset (human aging). (**D**) Fraction of SenFlag+ cells expressing downstream targets of indicated pathways in the Tabula Sapiens dataset, stratified by cell type. (**E**) Fraction of SenFlag+ cells expressing downstream targets of indicated pathways in irradiated murine tissue datasets (see Fig. 3I), stratified by cell type. (**F**) UMAP visualization of integrated murine scRNA-seq datasets from brain and blood-brain barrier tissues, showing cellular composition. (**G**) Abundance of SenFlag+ cells in brain datasets from panel (**F**), stratified by age. (**H**) Abundance of SenFlag+ cells in brain datasets from panel (**F**), stratified by cell type and age. (**I**) Abundance of *Cdkn1a/Cdkn2a*+, *Hmgb1/2/n2*-low cells stratified by *Ccnd1* positivity in single-nucleus (snRNA-seq) data.

 © The Author(s)

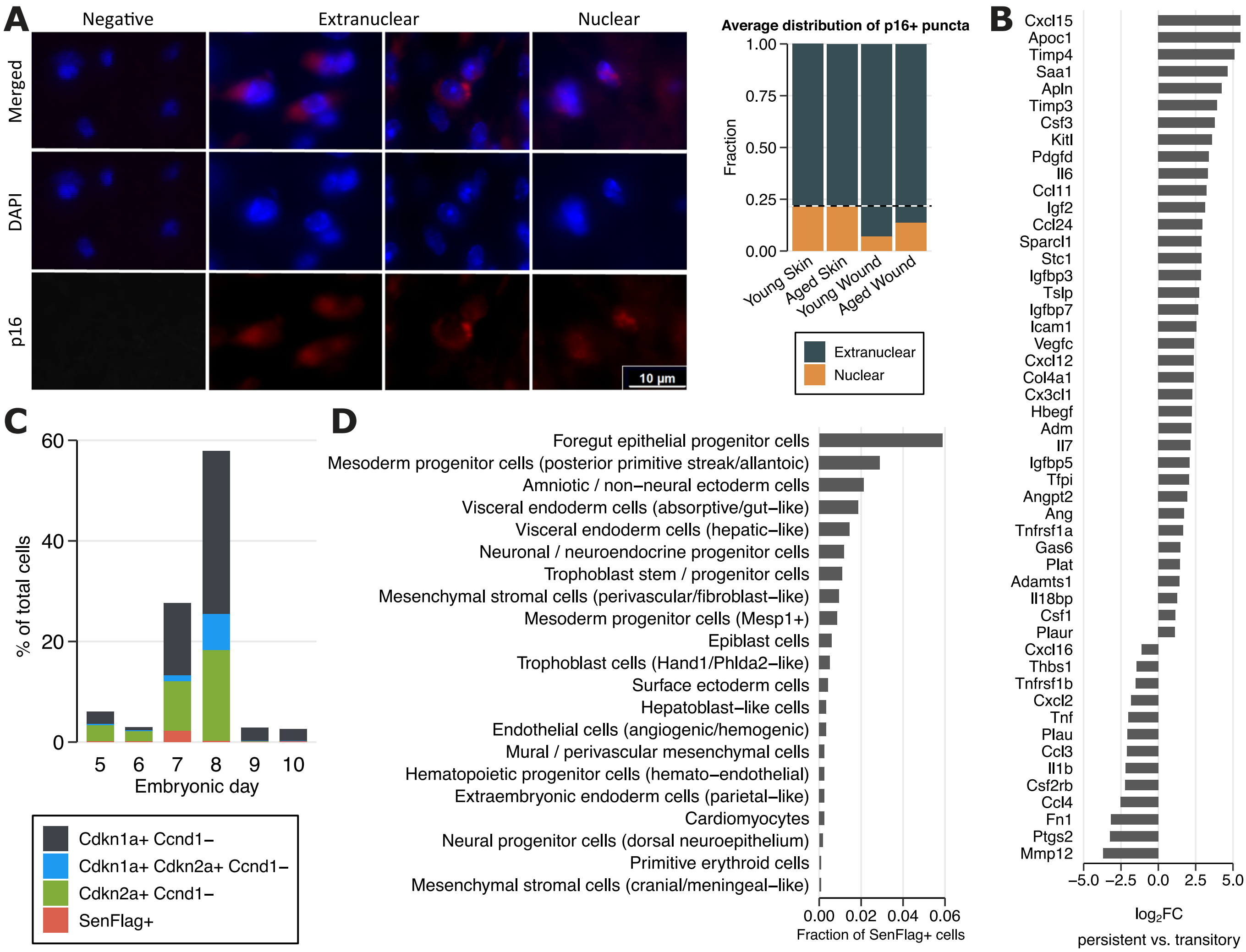

Figure EV4. Additional analyses of SenFlag+ cells across injury, aging, and developmental contexts.

(A) Immunofluorescence staining showing representative images of p16 subcellular distribution (negative, extranuclear, and nuclear) and corresponding quantification in young and aged, wounded and unwounded murine skin. The dashed line indicates the average fraction of p16 distribution in intact skin. **(B)** Log_2 fold change values ($p_{adj} < 0.05$; DESeq2) for indicated inflammation-associated genes comparing SenFlag+ cells from injury-associated datasets with those from aging and irradiation datasets. **(C)** Cellular distribution of *Cdkn1a/Cdkn2a*+ cells across the indicated murine developmental stages (in days), stratified by *Ccnd1* positivity and SenFlag status. **(D)** Cell type distribution of SenFlag+ cells in murine developmental datasets. Source data are available online for this figure.

© The Author(s)
